# Developing the script “degenerate primer 111” to enhance the coverage of universal primers for the small subunit rRNA gene on target microorganisms

**DOI:** 10.3389/fmicb.2024.1394303

**Published:** 2024-09-04

**Authors:** Zhihui Qin, Xin Xu, Fengjun Xu, Yao Zhang, Peng Su, Chaofeng Shen

**Affiliations:** ^1^Department of Environmental Engineering, College of Environmental and Resource Sciences, Zhejiang University, Hangzhou, China; ^2^Zhejiang Provincial Key Laboratory for Water Pollution Control and Environmental Safety, Hangzhou, China

**Keywords:** 16S rRNA, 18S rRNA, bacteria, archaea, eukaryote, high-throughput sequencing, *Dehalococcoides*

## Abstract

Amplifying small subunit (SSU) rRNA genes with universal primers in assessing microbial populations diversity, but target microorganisms are sometimes omitted due to inadequate primer coverage. Adding degenerate bases to primers can help, but existing methods are complex and time-consuming. This study introduces a user-friendly tool called “Degenerate primer 111” for adding degenerate bases to existing universal primers. By aligning one universal primer with one uncovered target microorganism’s SSU rRNA gene, this tool iteratively generates a new primer, maximizing coverage for the target microorganisms. The tool was used to modify eight pairs of universal primers (515F Parada–806R Apprill, S-D-Bact-0341-b-S-17/S-D-Bact-0785-a-A-21, OP_F114-KP_R013, 27F-1492R, 341F-806R, OP_F066-KP_R013, 515F Parada–926R Quince, 616*F-1132R), and generated 29 new universal primers with increased coverage of specific target microorganisms without increasing coverage of non-target microorganisms. To verify the effectiveness of the improved primers, one set of original and improved primers (BA-515F-806R and BA-515F-806R-M1) was used to amplify DNA from the same sample, and high-throughput sequencing of the amplicons confirmed that the improved primers detected more microbial species compared to the original primers. Future researchers can use this tool to develop more personalized primers to meet their diverse microorganism detection needs.

## 1 Introduction

Small subunit (SSU) rRNA, present in bacteria, archaea, and eukaryotes, includes 16S rRNA in bacteria and archaea, and 18S rRNA in eukaryotes, and is extensively used in microbial classification (Gray et al., 1984). Researchers design PCR primers targeting conserved SSU rRNA regions to amplify sequences with variable regions (V) and cluster them into Operational Taxonomic Units (OTUs) based on sequence similarity (Gray et al., 1984; Chaudhary et al., 2015). Some primers, like *Dehalococcoides*-targeted primer (Löffler et al., 2000), is taxon-specific, while others, known as “universal” primers, target a broad range of microorganisms, including bacterial, archaeal, and eukaryotic universal primers (Baker et al., 2003; Takahashi et al., 2014). Some commonly used universal primers with high coverage include: 515F (Parada)–806R (Apprill) for the bacterial and archaeal 16S rRNA gene V4 region, recommended by the Earth Microbiome Project (Apprill et al., 2015; Parada et al., 2016); 515F (Parada)–926R (Quince) for the bacterial, archaeal, and eukaryotic 16/18S rRNA gene V4-V5 region, also recommended by the Earth Microbiome Project (Earth Microbiome Project, n.d.; Quince et al., 2011; Parada et al., 2016); 341F-806R for the bacterial 16S rRNA gene V3-V4 region, recommended by Bio-protocol Protocol Database (Bio-protocol Protocol Database, n.d.; Li et al., 2021); S-D-Bact-0341-b-S-17/S-D-Bact-0785-a-A-21 for the bacterial 16S rRNA gene V3-V4 region, recommended by Illumina, Inc. (Illumina, n.d.; Klindworth et al., 2013); 27F-1492R for the full-length bacterial 16S rRNA gene, recommended by Callahan et al. (2019); OP_F114-KP_R013 for the archaeal 16S rRNA gene V3-V6 region, and OP_F066-KP_R013 for the V5-V6 region, recommended by Regueira-Iglesias et al. (2023); and 616*F-1132R for the eukaryotic 18S rRNA gene, recommended by Kounosu et al. (2019).

Universal primers cannot cover all microorganisms, possibly leading to missed detection. Eloe-Fadrosh et al. found that 9.6% of 16S rRNA genes in metagenomic sequences were not matched with commonly used primers (Eloe-Fadrosh et al., 2016). Regueira-Iglesias et al. showed that none of the 369 universal primers have 100% coverage (where “coverage” refers to the percentage of matches for a given taxonomic group) of 16S rRNA genes in the Silva database (Regueira-Iglesias et al., 2023). For instance, primer 515F-806R covers 83.6% of bacteria and 83.5% of archaea, but misses 62,406 bacterial species and 3,306 archaeal species. If these uncovered microorganisms are the focus of specific research, universal primers are unsuitable. For example, this study evaluated 20 pairs of primers from 55 research papers related to *Dehalococcoides* and found that either their coverage of *Dehalococcoides* was as low as 5.3%, or their coverage of total archaea or bacteria was low, making it difficult to explore coexisting microorganisms, or their coverage of eukaryotes was high, leading to data interference (Supplementary Table S1) (Integrated Microbiome Resource, n.d.). Consequently, these primers are inadequate for studying Dehalococcoides and its coexisting microorganisms, such as methanogens and hydrogen-producing bacteria (Yang and Mccarty, 1998; Yan et al., 2013). Therefore, to ensure the feasibility of universal primers for specific researches, the improvement of primers that match the target microorganisms is accretive.

The mismatch between universal primers and target microorganisms rises from differences in bases between the primers and the SSU rRNA gene of the target. Modifying the differing bases in universal primers to degenerate bases can achieve coverage of the target microorganism. For instance, in 2011, Caporaso et al. designed primers 515F/806R (F: *GTGCCAGCMGCCGCGGTAA*; R: *GGACTACHVGGGTWTCTAAT*) for the bacterial and archaeal V4 region using PrimerProspector software (Walters et al., 2011). Later, Hugerth et al. (2014) increased archaea coverage from 53 to 93% by changing the 4th position of the F primer from *C* to *Y* [*Y* is (*C*/*T*)] using the Degeprime software (Hugerth et al., 2014). Apprill et al. (2015) improved SAR11 bacteria detection by changing the 8th base of the Caporaso-806R primer from *H* [*H* is (*A*/*C*/*T*)] to *N* [*N* is (*A*/*C*/*T*/*G*)], increasing coverage from 2.6 to 96.7%. These two improvements were adopted in Parada’s 2016 paper and became the widely used Earth Microbiome Project recommended primers 515F (Parada)–806R (Apprill) (F: *GTGYCAGCMGCCGCGGTAA*; R: *GGACTACNVGGGTWTCTAAT*) (Apprill et al., 2015; Parada et al., 2016). Additionally, McNichol et al. (2021) designed workflows to assess the alignment of universal primers with metagenomes from the environment, and modified the universal primers to improve the match with metagenomes. In summary, the aforementioned operations involve multiple sequence alignments, reverse complementation of sequences, and base degeneracy but are complex and time-consuming. A user-friendly tool integrating these functions would help researchers customize universal primers according to specific research requirements more efficiently.

This study developed a tool for improving universal primers by progressively adding degenerate bases. The tool was used to personalize eight pairs of classic universal primers targeting different microorganisms to enhance coverage. Subsequently, one set of original primers and the corresponding improved primers were selected to amplify DNA from the same samples, followed by high-throughput sequencing of the amplicon to demonstrate the effect of primer improvement on real sample detection results.

## 2 Materials and methods

### 2.1 *In silico* evaluation of universal primers

The coverage of all primers was assessed using Silva (n.d.). Silva provides regularly updated datasets of aligned small (16S/18S, SSU) and large subunit (23S/28S, LSU) ribosomal RNA (rRNA) sequences for all three domains of life (Klindworth et al., 2013; Quast et al., 2013). The latest update, Silva SSU 138.1, was released in December 2019, increasing the number of available SSU sequences to 9,469,124 (Silva, n.d.).

Silva’s TestPrime page^1^ was accessed, the primer sequence was entered, the SSU R138.1 Database was selected, the maximum number of mismatches was set to 0, and “Run TestPrime” was selected to allow the system to automatically calculate the primer coverage.

### 2.2 Script development

The overall idea of the script is to identify the bases in the primers that do not match the SSU rRNA gene and replace them with degenerate bases. Specifically:

Firstly, the target gene is converted into its reverse complementary sequence, i.e., converting the sense strand to the antisense strand (this step is for the improvement of the reverse primer, while the improvement of the forward primer skips this step). The reverse complementary sequence is achieved through a two-step process: (1) Complementarity: Base pairs are replaced with their corresponding counterparts. A is replaced with *T*, *C* is replaced with *G*. (2) Reverse: The sequence from 5′ to 3′ is rearranged from 3′ to 5′, then treated as a new 5′ to 3′ sequence. For example, the sequence 5’ *AGGTAC* 3′ has a complementary sequence of 5′ *TCCATG* 3′, and the reverse complementary sequence is 5’ *GTACCT* 3′.

Next, locate the corresponding sequences in the gene by searching for bases that match the primer, and identify any mismatched bases. The determination of sequence identity includes exact match, degenerate match, and mismatch. Exact match refers to bases with the same name, such as *A* matches with *A*, *R* matches with *R*, and so on. Degenerate match refers to degenerate bases matching with the included bases, such as *G* matches with degenerate bases containing *G* (e.g., *R*, *K*, *S*, *B*, *V*, *D*, *N*), and so on. Any cases other than exact match and degenerate match are considered as mismatches. When the number of mismatched bases exceeds 5, it is considered invalid. The threshold for mismatched bases is set at <5 for the following reasons: if there are more than 5 differences, the sequence may not be the primer corresponding sequence; even if the sequence is the corresponding sequence, it means no improvement. This is because the minimum product of degeneracy for five bases is 32, adding one more degenerate base exceeds 64, meaning it would generate 64 different primer sequences, while SILVA recommends that degenerate primers correspond to no more than 60 sequences (Quast et al., 2013).

Finally, bases in the primer that are different from the gene sequence are replaced with degenerate bases, preferably with 2-base degenerate bases, if not possible, 3-base degenerate bases are chosen, and if still not possible, 4-base degenerate bases are chosen. The replacement strategy is as follows: *A*/*G* = *R* (meaning if the different bases between the primer and the gene are *A* and *G*, then the primer bases are changed to degenerate base *R*), *C*/*T* = *Y*, *A*/*C* = *M*, *G*/*T* = *K*, *G*/*C*=*S*, *A*/*T* = *W*, *A*/*Y*=*H*, *T*/*M* = *H*, *C*/*W*=*H*, *G*/*Y*=*B*, *T*/*S*=*B*, *C*/*K*=*B*, *G*/*M* = *V*, *A*/*S*=*V*, *C*/*R* = *V*, *G*/*W*=*D*, *A*/*K*=*D*, *T*/*R* = *D*, *A*/*B*=*N*, *T*/*V*=*N*, *C*/*D*=*N*, *G*/*H*=*N*.

### 2.3 Improving universal primers with the script

**Figure 1:**
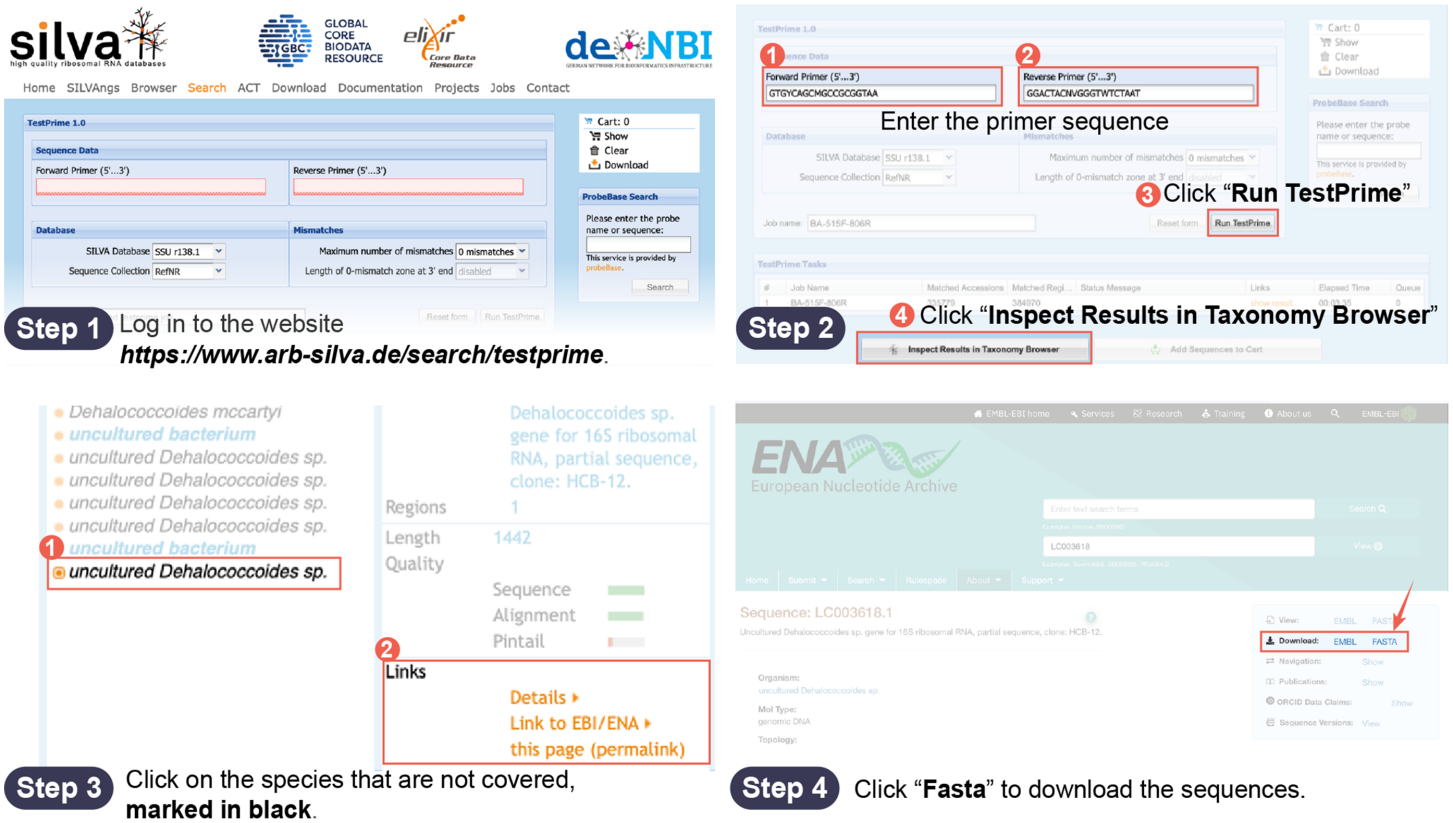
Flowchart for primer coverage evaluation and download of uncovered SSU rRNA genes.

**Figure 2:**
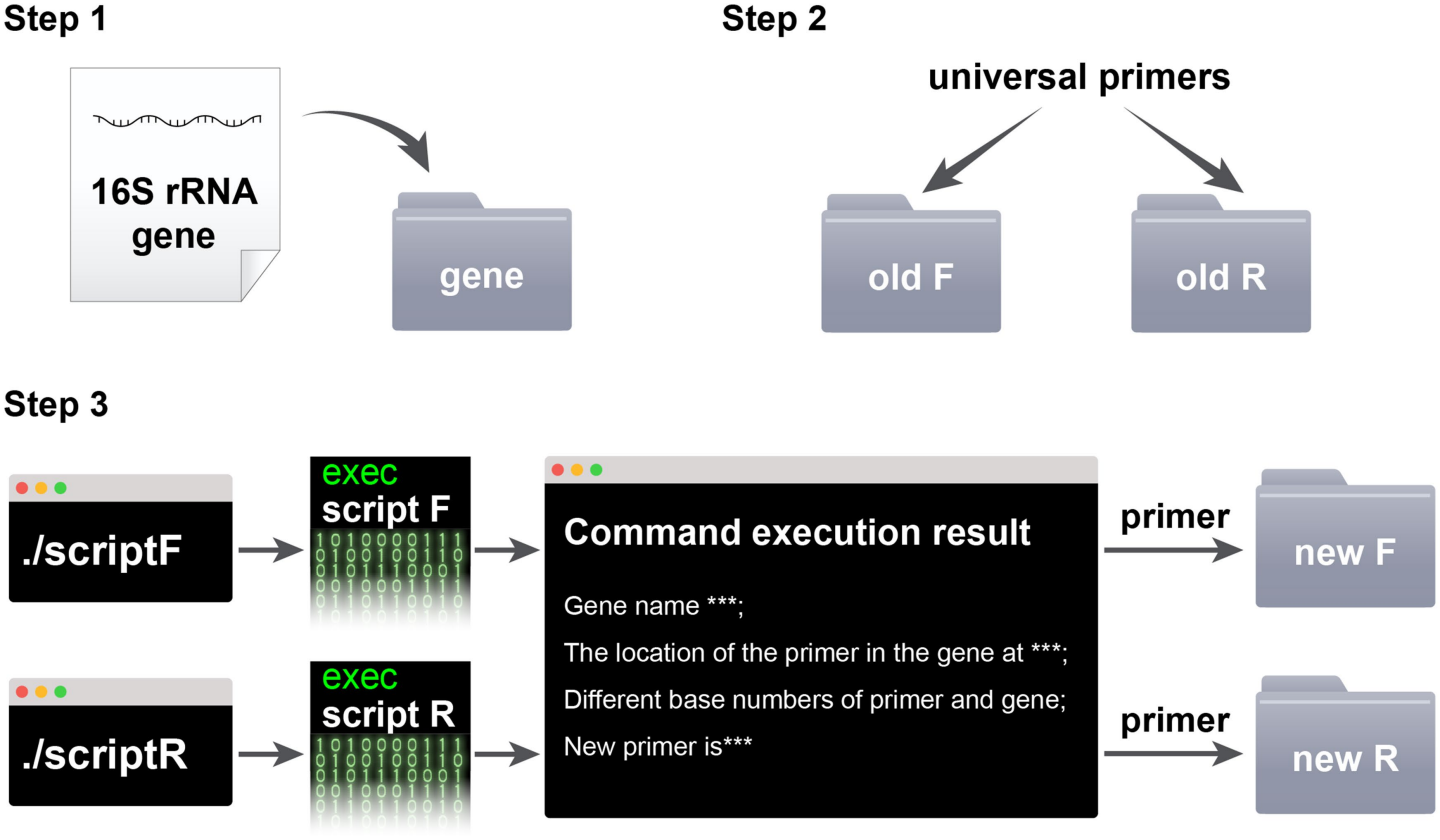
The script’s usage workflow.

The steps for using the script in conjunction with the Silva database are as follows: Step 1: Prepare the SSU rRNA gene. Evaluate the universal primers in the Silva database, and then download a SSU rRNA gene sequence from the target microorganism that is not covered by the universal primers and place it in the “gene” folder. The primer coverage assessment and gene download process are illustrated in Figure 1. Step 2: Prepare the primers. Place the forward primer (F primer) and reverse primer (R primer) of the universal primers to be improved into the “old F” and “old R” folders, respectively. Step 3: Run the commands. Execute the “script F” or “script R” command in the macOS terminal or Linux shell. Step 4: Collect the new primers. The new primers will be displayed in the running interface and in the “new F/R” folder. Replace the old primers with the new ones and repeat steps 1–4 for iterative improvement until Silva indicates that the new primers are invalid due to containing multiple degenerate bases, resulting in more than 60 primer sequences. The last round of effective primers is considered the final improved primers. The script usage process is illustrated in Figure 2.

This study selected 8 pairs of classic primers as examples to demonstrate the personalized improvement using the “Degenerate primer” tool to enhance the coverage of target microorganisms. Microorganisms with coverage below 100% were considered target microorganisms, and this study only selected some of microorganisms with low coverage. For convenience, this study renamed these primers using B, A, and E to represent bacteria, archaea, and eukaryotes, respectively, with names indicating the start position of the F primer and the end position of the R primer on the forward strand of the SSU rRNA gene. These primers and their corresponding microorganisms are as follows:

1. The universal primers 515F (Parada)–806R (Apprill) is renamed BA-515F-806R (F: *GTGYCAGCMGCCGCGGTAA*, R: *GGACTACNVGGGTWTCTAAT*), recommended by the Earth Microbiome Project for amplifying the V4 region of the 16S rRNA gene in bacteria and archaea, were improved for three bacterial taxa (*g_Dehalococcoides*: 5.3%, *p_Calescamantes*: 0%, and *g_Caldisericaceae*: 0%) and two archaeal taxa (*p_Iainarchaeota*: 4.4% and *p_Nanohaloarchaeota*: 0%) (Earth Microbiome Project, n.d.; Apprill et al., 2015; Parada et al., 2016).
2. The universal primers S-D-Bact-0341-b-S-17/S-D-Bact-0785-a-A-21 is renamed B-341F-806R (F: *CCTACGGGNGGCWGCAG*, R: *GACTACHVGGGTATCTAATCC*), recommended by Illumina, Inc. for amplifying the V3-V4 region of the 16S rRNA gene in bacteria, were improved for five bacterial taxa (*p_Aerophobota*: 2.9%, *p_Deferrisomatota*: 0.0%, *p_Fermentibacterota*: 0.0%, *p_Calescamantes*: 0.0%, and *p_PAUC34f*: 1.1%) (Illumina, n.d.; Klindworth et al., 2013).
3. The universal primers OP_F114-KP_R013 is renamed A-341F-1049R (F: *CCTAYGGGRBGCASCAG*, R: *GGCCATGCACCWCCTCTC*), recommended by Alba Regueira-Iglesias et al. for amplifying the V3-V6 region of the 16S rRNA gene in archaea, were improved for four archaeal taxa (*p_Nanohaloarchaeota*: 0%, *p_Korarchaeota*: 0%, *p_Micrarchaeota*: 2.6%, and *p_Altiarchaeota*: 0%) (Regueira-Iglesias et al., 2023).
4. The universal primers 27F-1492R is renamed B-27F-1492R (F: *AGRGTTYGATYMTGGCTCAG*, R: *RGYTACCTTGTTACGACTT*), recommended by Callahan et al. for amplifying the full-length 16S rRNA gene in bacteria, were improved for six bacterial taxa (*p_10bav-F6*: 0%, *p_Apal-E12*: 0%, *p_Fervidibacteria*: 0%, *p_MAT-CR-M4-B07*: 0%, *p_TX1A-33*: 0%, and *p_Fusobacteriota*: 31.3%) (Callahan et al., 2019).
5. The universal primers 341F-806R is renamed BA-341F-806R (F: *CCTAYGGGRBGCASCAG*, R: *GGACTACNNGGGTATCTAAT*), recommended by Bio-protocol Protocol Database (Bio-protocol Protocol Database, n.d.; Li et al., 2021), for amplifying the V3-V4 region of the 16S rRNA gene in bacteria and archaea, were improved for four bacterial taxa (*p_Calescamantes*: 0%, *p_Aerophobota*: 2.9%, *p_Deferrisomatota*: 0%, *p_Fermentibacterota*: 0%) and two archaeal taxa (*p_Asgardarchaeota*: 3.7% and *p_Iainarchaeota*: 7.3%).
6. The universal primers OP_F066-KP_R013 is renamed A-784F-1049R (F: *GGMTTAGATACCC*, R: *GGCCATGCACCWCCTCTC*), recommended by Regueira-Iglesias et al. for amplifying the V5-V6 region of the 16S rRNA gene in archaea, were improved for four archaeal taxa (*p_Nanohaloarchaeota*: 0%, *p_Korarchaeota*: 1.7%, *p_Micrarchaeota*: 1.9%, and *p_Altiarchaeota*: 2.8%) (Regueira-Iglesias et al., 2023).
7. The universal primers 515F (Parada)–926R (Quince) is renamed BAE-515F-926R (F: *GTGYCAGCMGCCGCGGTAA*, R: *CCGYCAATTYMTTTRAGTTT*), recommended by the Earth Microbiome Project for amplifying the V4-V5 region of the 16S/18S rRNA genes in bacteria, archaea, and eukaryotes, were improved for two eukaryotic taxa (*p_Excavata*: 6.0% and *p_Discoba*: 9.1%), two bacterial taxa (*p_Fervidibacteria*: 0% and *p_Poribacteria*: 0%), and two archaeal taxa (*p_Altiarchaeota*: 7.5% and *p_Nanohaloarchaeota*: 0%) (Earth Microbiome Project, n.d.).
8. The universal primers 616*F-1132R is renamed E-616F-1132R (F: *TTAAARVGYTCGTAGTYG*, R: *CCGTCAATTHCTTYAART*), recommended by Asuka Kounosu et al. for amplifying the 18S rRNA gene in eukaryotes, were improved for two eukaryotic taxa (*p_Excavata*: 4.9% and *p_Discoba*: 61.2%) (Kounosu et al., 2019).

### 2.4 Primer evaluation via SSU rRNA gene high-throughput sequencing of environmental samples

Using both the original primers and the improved primers to amplify the microbial SSU rRNA genes of the same sample can be employed to compare the impact of primer coverage on actual detection results. To ensure the presence of the targeted microorganisms in the samples, this study extracted DNA from 8 typical environmental samples (soil, sediment, soil-derived cultures, and sediment-derived cultures) known to contain *Dehalococcoides*. The improved primers specific for *Dehalococcoides* BA-515F-806R-M1 and the original primers BA-515F-806R were used to amplify and sequence the same SSU rRNA gene from the same sample for comparison.

The microbial DNA in the environmental sample test groups was extracted using a PowerSoil^®^ DNA Isolation Kit (Mobio Laboratories, Inc., Carlsbad, CA, United States) according to the manufacturer’s instructions. The SSU rRNA gene was amplified using the two pairs of primer and sequenced by the Illumina MiSeq platform. The Thermal cycling program is set as follows: 95°C for 3 min; 95°C for 30 s, 55°C for 30 s, 72°C for 45 s, a total of 25 cycles; 72°C for 5 min; Hold at 4°C. The sequencing results were processed by the Shanghai Shenggong Biotechnology Co., Ltd. and related software Usearch et al. to obtain the final OTU information (Edgar, 2013, 2016), which was then prepared for the downstream data analysis.

This study compared the differences in the number of detected microbial species between the unimproved primer BA-515F-806R and the improved primer BA-515F-806R-M1 when amplifying the same sample using OTU counts. Additionally, the study investigated the differences in the number of detected species at the genus level for *g_ Dehalococcoides* and nine other genera (*g_Inhella*, *g__Methylomicrobium*, *g_Caminicella*, *g_Myroides*, *g_Dokdonella*, *g_Desulfovibrio*, *g_Pedomicrobium*, *g_Lewinella*, *g__Turicibacter*), which exhibited increased coverage with BA-515F-806R-M1, between amplification with the improved and unimproved primers in the same sample.

Raw data of high throughput sequencing of SSU rRNA gene have been uploaded to the NCBI Sequence Read Archive database under BioProject ID PRJNA1047931.

## 3 Results

### 3.1 The script “degenerate primer 111”

This study developed a script to improve universal primers, named “Degenerate primer 111.” This tool, used in conjunction with the Silva website, aligns “1” universal primer to “1” target microbial SSU rRNA gene not covered by the universal primer, generating “1” new universal primer covering the target gene. Iterative runs with the new universal primer and its uncovered SSU rRNA gene produce a new set of universal primers, ultimately maximizing coverage of the target microorganism by the universal primers. On the author’s Mac M2 computer, the evaluation of primers using the Silva database generally takes around 3 min, searching and downloading genes can be completed within 2 min, and the script execution time is approximately 5 min per run, making the entire process take about 10 min. A screening recording of the script’s operation is placed in the Supplementary material. The script has been uploaded to GitHub and is available at https://github.com/haojunsp/script.git.

### 3.2 Personalized improvement of universal primers to enhance coverage of target microorganisms

All eight universal primers (BA-515F-806R, BA-341F-806R, B-341F-806R, A-341F-1059R, A-784F-1059R, B-27F-1492R, BAE-515F-926R, E-616F-1132R) could be improved for targeting specific microorganisms using the “Degenerate primer 111” script. The study obtained 29 personalized primer pairs, which increased coverage of target microorganisms as well as taxa within the same domain, without affecting unrelated domains. The primer sequences and coverage changes are presented in Table 1 and Supplementary Table S2, and the script run data is available in Supplementary material.

**Table 1:** Improvements to commonly used primers to meet various detection needs.

Primer name	Target microorganism	Forward primer	Reverse primer	Species number	Species coverage				
					Unimproved	Improved	Archaea	Bacteria	Eukaryote
BA-515F-806R	–	*GTGYCAGCMGCCGCGGTAA*	*GGACTACNVGGGTWTCTAAT*	–	–	–	83.5%	83.6%	0.1%
BA-515F-806R-M1	*Dehalococcoides*	*GTGYCAGCMGCCGCGGTAA*	*GGACTACNVG**R**GTWTCTAAT*	38	5.3%	92.1%	83.5%	83.8%	0.1%
BA-515F-806R-M2	*Calescamantes*	*GTGYCAGCMGCCGCGGTAA*	*GGACTACNVGGGT**H**TCTAAT*	3	0.0%	100.0%	84.3%	83.7%	0.1%
BA-515F-806R-M3	*Caldisericaceae*	*GTGYCAGCMGC**Y**GCGGTAA*	*GGACTACNVGGGTWTCTAAT*	33	0.0%	93.9%	83.6%	83.9%	0.1%
BA-515F-806R-M4	*Iainarchaeota*	*GTGYCAGCMGCCGCGGTAA*	*GGACTA**M**NVGGGTWTCTAAT*	45	4.4%	71.1%	83.9%	83.7%	0.1%
BA-515F-806R-M5	*Nanohaloarchaeota*	***S**TGYCAGCMGCCGCGGTAA*	*GGACTACNVGGGTWTCTAAT*	34	0.0%	70.6%	83.7%	83.7%	0.1%
A-341F-1059R	–	*CCTAYGGGRBGCASCAG*	*GGCCATGCACCWCCTCTC*	–	–	–	71.4%	0.0%	0.0%
A-341F-1059R-M1	*Nanohaloarchaeota*	*CCTAYGGGRBGCASCAG*	*GGCCA**Y**GCA**V**CWCCTCTC*	34	0.0%	97.0%	72.4%	0.0%	0.0%
A-341F-1059R-M2	*Korarchaeota*	*CCTA**H**GGGRBGCASCAG*	*GGCCA**Y**GCACCWCC**Y**CTC*	62	0.0%	82.1%	72.6%	0.0%	0.0%
A-341F-1059R-M3	*Micrarchaeota*	*CCTAYGGGRBGCASCAG*	***S**GCCATGCA**VY**WC**Y**TCTC*	79	2.6%	73.7%	78.4%	0.0%	0.0%
A-341F-1059R-M4	*Altiarchaeota*	*CCTAYGGGRBGCA**B**CAG*	*GGCCATGCAC**Y**WCC**Y**CTC*	41	0.0%	32.0%	74.7%	0.0%	0.0%
B-341F-806R	–	*CCTACGGGNGGCWGCAG*	*GACTACHVGGGTATCTAATCC*	–	–	–	0.3%	82.8%	0.0%
B-341F-806R-M1	*Aerophobota*	*CCTA**Y**GGGNGGCWGCAG*	*GACTACHVGGGT**M**TCTAATCC*	70	2.9%	90.0%	0.3%	83.6%	0.0%
	*Deferrisomatota*			26	0.0%	92.3%			
	*Calescamantes*			3	0.0%	100.0%			
B-341F-806R-M2	*PAUC34f*	*CCTACGGGNG**S**CWGCAG*	*GAC**Y**ACHVGGGTATCTAATCC*	93	1.1%	84.9%	0.3%	82.9%	0.0%
B-341F-806R-M3	*Fermentibacterota*	*CCT**H**CGGGNGGCWGCAG*	*GACTACHVGGGTATCTAATCC*	54	0.0%	87.0%	0.3%	83.0%	0.0%
B-27F-1492R	–	*AGRGTTYGATYMTGGCTCAG*	*RGYTACCTTGTTACGACTT*	–	–	–	0.1%	71.6%	0.2%
B-27F-1492R-M1	*Fusobacteriota*	*AGRGTTYGATYMTGGCTCAG*	***DR**Y**KR**CCTTGTTACGACTT*	201	31.3%	33.3%	0.1%	71.6%	0.2%

Modified bases are highlighted in bold.

**Figure 3:**
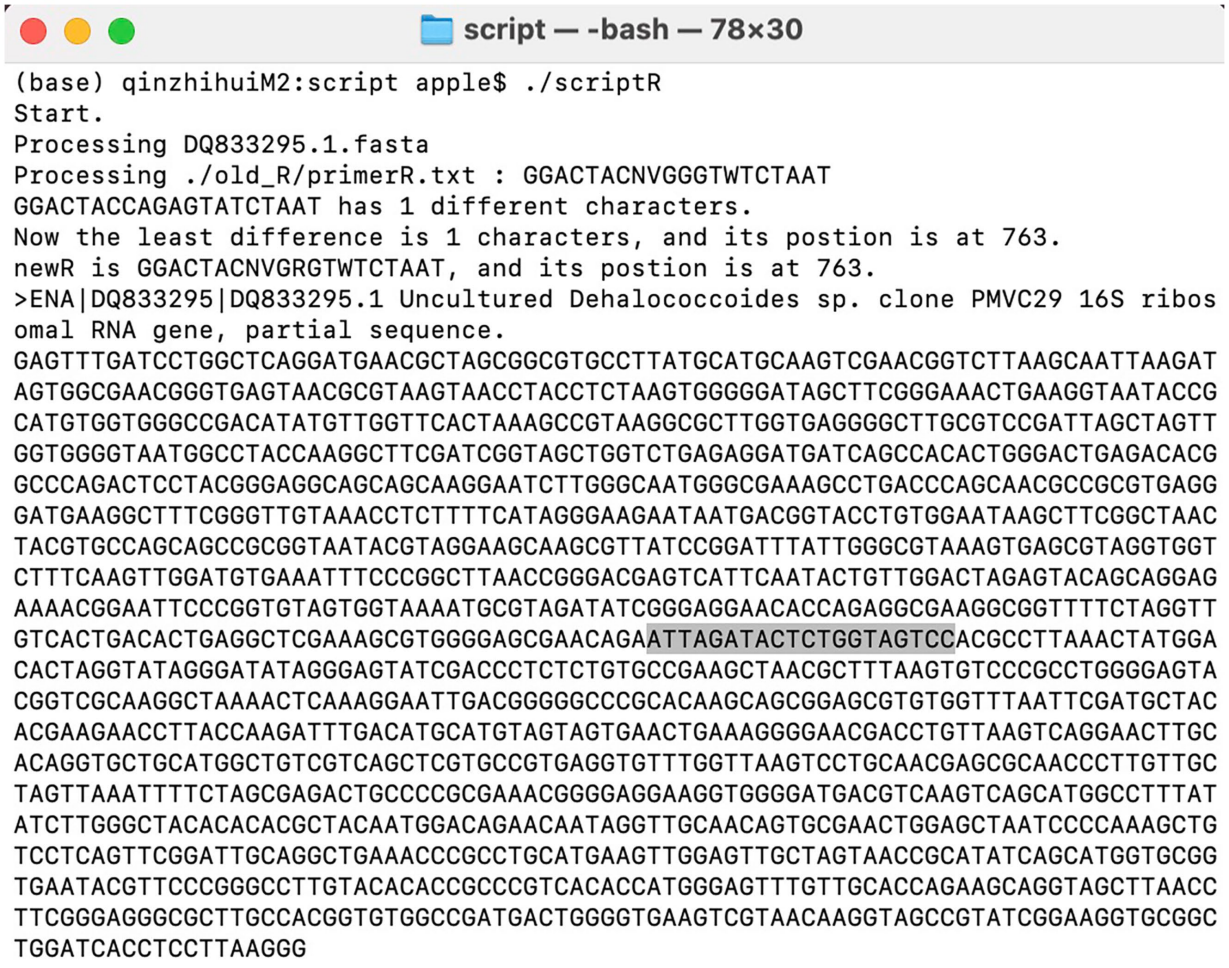
Script execution interface. The position of primers is influenced by sequence integrity and may not necessarily represent true values. Researchers can make judgments based on approximate positions.

**Figure 4:**
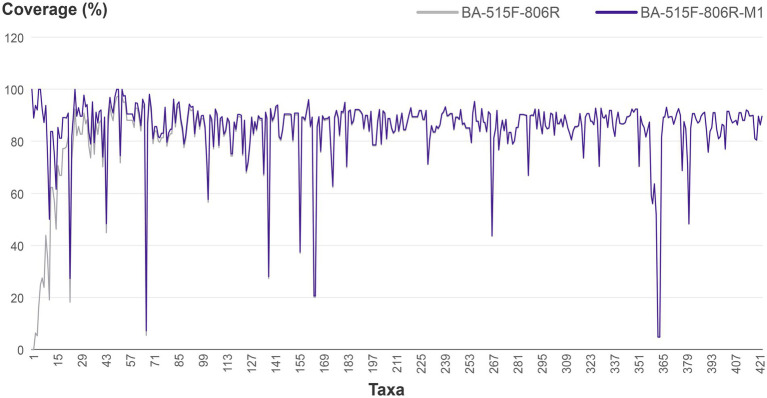
Comparison of the coverage of the original primer BA-515F-806R and the improved primer BA-515F-806R-M1 (taking the first 423 taxa). The vertical axis represents coverage, while the horizontal axis represents microbial taxa that do not distinguish between kingdom, phylum, class, order, family, genus, and species.

In most cases, improved primers were obtained after 1–2 iterations. For instance, improving primer BA-515F-806R targeting *Dehalococcoides*. In the Silva database, there are a total of 38 16S rRNA gene sequences for *Dehalococcoides*, out of which 36 are not covered by the primer 515F-806R. One of the *Dehalococcoides* 16S rRNA genes, which was randomly chosen from those not covered by 515F-806R, was compared to the primer 515F-806R. Forward primer comparison result showed no difference in bases between the target 16S rRNA gene of *Dehalococcoides* and the 515F primer (*GTGYCAGCMGCCGCGGTAA*), while reverse primer comparison results revealed one mismatch between the *Dehalococcoides* 16S rRNA gene (*GGACTACCAGAGTATCTAAT*) and the 806R primer (*GGACTACNVGGGTWTCTAAT*), specifically, a G-to-A mismatch. The degenerate base for this mismatch was assigned as R, resulting in the new reverse primer sequence *GGACTACNVGRGTWTCTAAT*. The script running interface was shown in Figure 3. Subsequent iterations revealed that three *Dehalococcoides* 16S rRNA genes remained uncovered by the new primer. Two of them had two different bases compared to the previous new R primer, and one had more than three differences from the 515F primer, rendering it ineffective to modify them further due to excessive degenerate bases. Consequently, the previous effective sequence was determined as the final improved primer. The new R primer, in combination with the 515F primer, formed the new primer (515F-806R)-M. Evaluation with the Silva database showed an increase in the coverage of *Dehalococcoides* from 5.3 to 92.1%. The coverage of 421 other bacterial taxa also increased to varying degrees, resulting in an overall bacterial coverage increase from 83.6 to 83.8%, as shown in Figure 4. The coverage of Archaea remained unchanged at 83.5%. Meanwhile, the coverage for eukaryotes remained at 0.1%.

There were cases in this study where universal primers could not be improved. When attempting to improve primer B-27F-1492R for *p_10bav-F6*, *p_Apal-E12*, *p_Fervidibacteria*, *p_MAT-CR-M4-B07*, and *p_TX1A-33*, the sequences provided by Silva were shorter than the target primer length, resulting in unsuccessful improvements.

The study also involves modifying the initial primers to adapt to primer improvements. When improving primer E-616F-1132R for *p_Excavata*, it was found that F primer could only accommodate an additional single degenerate base, while the actual situation required two. Luckily, one of the degenerate bases was at the terminal position, so it was removed to ensure that the number of degenerate bases did not exceed the limit allowed by Silva. Researchers could employ other flexible methods, such as reducing the degeneracy of the original primer to make room for introducing new degenerate bases.

### 3.3 Higher coverage for *Dehalococcoides* and other bacterias in real samples with improved primers

**Figure 5:**
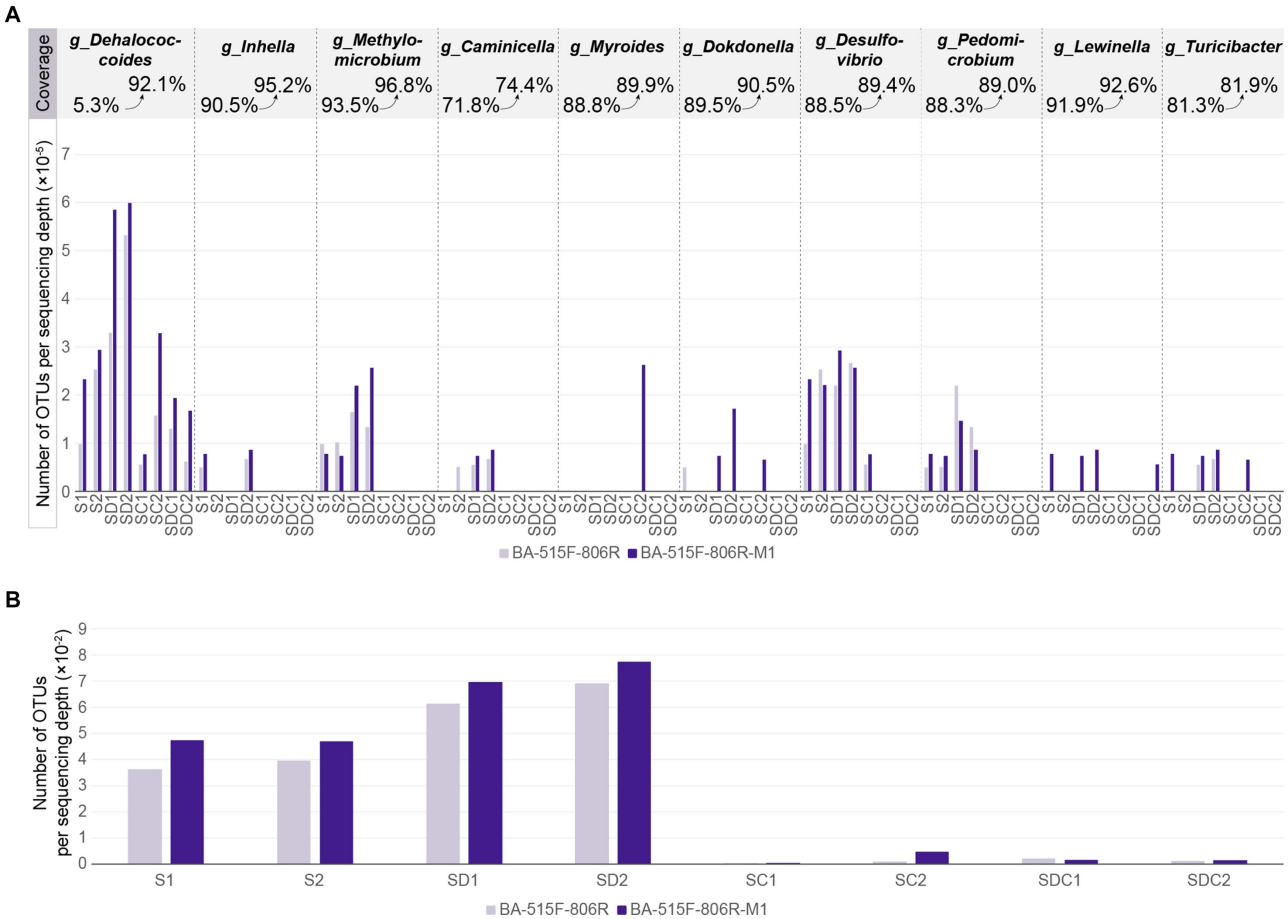
**(A)** Number of OTUs per sequencing classified as *Dehalococcoides* and other nine genera in each sample. **(B)** Number of OTUs per sequencing depth in each sample. The x-axis represents the sample names, where S stands for Soil, SD stands for Sediment, SC stands for Soil-derived Culture, SDC stands for Sediment-derived Culture, and Arabic numerals indicate the serial number of samples within the same category.

In most cases, improved primers can detect a greater variety of bacteria within the same sample. This study compared the difference in the number of microbial species detected using unimproved primers BA-515F-806R and improved primers BA-515F-806R-M1 in eight samples. In seven samples (S1, S2, SD1, SD2, SC1, SC2, SDC2), the improved primers detected more species per sequencing depth than the unimproved ones, with increases of 1.3-fold, 1.2-fold, 1.1-fold, 1.1-fold, 1.2-fold, 4.6-fold, and 1.2-fold, respectively. In one sample (SDC1), the improved primers detected fewer species per sequencing depth compared to the unimproved ones, at 0.7-fold (Figure 5B). This study compared the difference in the number of species detected at the genus level by improved and unimproved primers for 10 taxa with increased coverage by BA-515F-806R-M1 in the same sample. This effectively tested the improvement of primers using 80 actual samples. In total, 41 samples showed no detection with either primer, while 39 samples were detected with at least one primer, with 31 samples showing detection of more species with the improved primer BA-515F-806R-M1 and 8 samples showing detection of fewer species with the improved primer. Specifically, among samples where the coverage of *g_Dehalococcoides* increased from 5 to 92%, all 8 samples showed increased detection. Among samples where the coverage of *g_Inhella* increased from 90.5 to 95.2%, all 2 samples showed increased detection. Among samples where the coverage of *g_Methylomicrobium* increased from 93.5 to 96.8%, 4 samples showed increase, 2 showed decrease. Among samples where the coverage of *g_Caminicella* increased from 71.8 to 74.4%, 3 samples showed increase, 1 showed decrease. Among samples where the coverage of *g_Myroides* increased from 88.8 to 89.9%, all 1 sample showed increased detection. Among samples where the coverage of *g_Dokdonella* increased from 89.5 to 90.5%, 4 samples showed increase, 1 showed decrease. Among samples where the coverage of *g_Desulfovibrio* increased from 88.5 to 89.4%, 5 samples showed increase, 2 showed decrease. Among samples where the coverage of *g_Pedomicrobium* increased from 88.3 to 89.0%, 4 samples showed increase, 2 showed decrease. Among samples where the coverage of *g_Lewinella* increased from 91.9 to 92.6%, all 4 samples showed increased detection. Among samples where the coverage of *g_Turicibacter* increased from 81.3 to 81.9%, all 4 samples showed increased detection (Figure 5A).

## 4 Discussion

Personalized universal primers meet specific research needs better. Numerous studies have focused on identifying the optimal primers for high coverage of the SSU rRNA gene, greatly facilitating research into microbial community structure (Klindworth et al., 2013; Kounosu et al., 2019; Regueira-Iglesias et al., 2023). However, previous studies and this one showed that there is no single universal primer that covers all microorganisms (Regueira-Iglesias et al., 2023). Even though some combinations, such as BA-515F-806R-M5 with BA-515F-806R-M1, BA-515F-806R-M2, or BA-515F-806R-M4, can increase coverage, they still cannot be combined indefinitely to cover all microorganisms. Therefore, improving universal primers based on specific research targets can better meet diverse research needs.

This study developed a user-friendly, rapid-response, quantifiable tool called “Degenerate primer 111” to enhance the coverage of universal primers for target microorganisms. Unlike previous methods like PrimerProspector, DegePrime, and the workflow by Jesse McNichol, which require multiple scripts and high expertise to find conserved regions through multiple sequence alignment and then design degenerate primers, this tool uses a stepwise strategy of adding degenerate bases to existing universal primers (Walters et al., 2011; Hugerth et al., 2014; McNichol et al., 2021). It aligns a universal primer with an uncovered SSU rRNA gene to iteratively generate new primers, reducing the workload and time required for multiple sequence alignments. The tool simplifies the process to a single script, where users only need to drop primer and gene files into folders and execute commands. However, its effectiveness is limited by the potential for improvement in the original primers. If significant differences exist between the target SSU rRNA gene and the original primers, users may need to replace the primers or manually reduce the degeneracy to allow for further improvement. Additionally, it does not consider other primer design factors like GC content and primer dimers, which can be evaluated using other software or experiments (Oligo Analyzer in IDT, n.d.).

This study demonstrated the feasibility of improving universal primers for diverse research using the “Degenerate primer 111” script, and showcased its wide applicability through personalized improvements using several primers. Most improvements in this study achieved high coverage rates after only 1–2 iterations. However, there were due to sequence differences and database completeness. For example, when improving the BA-341F-806R primer for *Asgardarchaeota*, too many sequences required more degenerate bases than allowed, resulting in moderate coverage. Researchers can make more attempts or use different primers. Additionally, the completeness of the Silva database affects results, as seen with the B-27F-1492R primer, where incomplete sequences led to mismatches that did not reflect real-world diversity. This study only demonstrated partial improvement, and there are still many target microorganisms that cannot be covered by universal primers. Researchers can use this method to meet their specific research needs.

This study compared the performance of original and improved primers in actual samples. Microorganisms with increased coverage were detected with more species overall, though some individual samples showed a decrease. Detection was influenced by: (1) Presence of target microorganisms in the sample. If absent, they cannot be detected regardless of primer coverage. In 41 samples, both primers yielded zero detection, likely due to this. (2) The primers failed to cover the microorganisms in the sample. This issue is not limited to the false negatives caused by less than 100% primer coverage, but also includes microorganisms present in the sample that are not listed in the Silva database, even if Silva evaluates their coverage as 100% (Quast et al., 2013). (3) Sequencing errors, particularly when the target microorganism abundance is low (Sims et al., 2014; Schirmer et al., 2015). Despite these factors, improving the coverage of universal primers for target microorganisms based on existing data (e.g., Silva) is a feasible approach to minimize the inevitable limitations of PCR-based detection techniques, thus providing a more accurate reflection of sample diversity (Eckert and Kunkel, 1991; Eloe-Fadrosh et al., 2016).

In summary, before using high-throughput SSU rRNA technology for microbial ecology research, it is essential to evaluate the coverage of universal primers for the target microorganisms. If the primers do not match well with the target microorganisms, they can be modified to increase degeneracy. The “Degenerate Primer 111” tool is very useful for this purpose.

## Data Availability

The datasets presented in this study can be found in online repositories. The names of the repository/repositories and accession number(s) can be found in the article/Supplementary material.

## Footnotes

^1^https://www.arb-silva.de/search/testprime/

## References

[ref1] Apprill A. Mcnally S. Parsons R. Weber L. (2015). Minor revision to V4 region SSU rRNA 806R gene primer greatly increases detection of SAR11 bacterioplankton. Aquat. Microb. Ecol. 75, 129–137. doi: 10.3354/ame01753

[ref2] Baker G. C. Smith J. J. Cowan D. A. (2003). Review and re-analysis of domain-specific 16S primers. J. Microbiol. Methods 55, 541–555. doi: 10.1016/j.mimet.2003.08.009, PMID: 14607398

[ref3] Bio-protocol Protocol Database (n.d.). Available at: https://bio-protocol.org/exchange/minidetail?id=9320620&type=30 (Accessed May 20, 2024).

[ref4] Callahan B. J. Wong J. Heiner C. Oh S. Theriot C. M. Gulati A. S. . (2019). High-throughput amplicon sequencing of the full-length 16S rRNA gene with single-nucleotide resolution. Nucleic Acids Res. 47:E103. doi: 10.1093/NAR/GKZ569, PMID: 31269198 PMC6765137

[ref5] Chaudhary N. Sharma A. K. Agarwal P. Gupta A. Sharma V. K. (2015). 16S classifier: a tool for fast and accurate taxonomic classification of 16S rRNA hypervariable regions in metagenomic datasets. PLoS One 10:e0116106. doi: 10.1371/journal.pone.0116106, PMID: 25646627 PMC4315456

[ref6] Earth Microbiome Project (n.d.). Available at: https://earthmicrobiome.org/protocols-and-standards/16s/ (Accessed July 15, 2023).

[ref7] Eckert K. A. Kunkel T. A. (1991). DNA polymerase Fidelity and the polymerase chain reaction. PCR Methods Appl. 1, 17–24. doi: 10.1101/gr.1.1.17 1842916

[ref8] Edgar R. C. (2013). UPARSE: highly accurate OTU sequences from microbial amplicon reads. Nat. Methods 10, 996–998. doi: 10.1038/nmeth.2604, PMID: 23955772

[ref9] Edgar R. C. (2016). SINTAX: a simple non-Bayesian taxonomy classifier for 16S and ITS sequences. bioRxiv. doi: 10.1101/074161

[ref10] Eloe-Fadrosh E. A. Ivanova N. N. Woyke T. Kyrpides N. C. (2016). Metagenomics uncovers gaps in amplicon-based detection of microbial diversity. Nat. Microbiol. 1:15032. doi: 10.1038/NMICROBIOL.2015.32, PMID: 27572438

[ref11] Gray M. W. Sankoff D. Cedergren R. J. (1984). On the evolutionary descent of organisms and organdies: a global phytogeny based on a highly conserved structural core in small sabunit ribosomal RNA. Nucleic Acids Res. 12, 5837–5852. doi: 10.1093/nar/12.14.5837, PMID: 6462918 PMC320035

[ref12] Hugerth L. W. Wefer H. A. Lundin S. Jakobsson H. E. Lindberg M. Rodin S. . (2014). DegePrime, a program for degenerate primer Design for Broad- Taxonomic-Range PCR in microbial ecology studies. Appl. Environ. Microbiol. 80, 5116–5123. doi: 10.1128/AEM.01403-14, PMID: 24928874 PMC4135748

[ref13] Illumina (n.d.). Illumina 16S metagenomics sequencing workflow. Available at: https://support.illumina.com/content/dam/illumina-marketing/documents/products/other/16s-metagenomics-faq-1270-2014-003.pdf (Accessed July 15, 2023).

[ref14] Integrated Microbiome Resource (n.d.). Available at: https://imr.bio/protocols.html (Accessed July 15, 2023).

[ref15] Klindworth A. Pruesse E. Schweer T. Peplies J. Quast C. Horn M. . (2013). Evaluation of general 16S ribosomal RNA gene PCR primers for classical and next-generation sequencing-based diversity studies. Nucleic Acids Res. 41, e1–e11. doi: 10.1093/nar/gks808, PMID: 22933715 PMC3592464

[ref16] Kounosu A. Murase K. Yoshida A. Maruyama H. Kikuchi T. (2019). Improved 18S and 28S rDNA primer sets for NGS-based parasite detection. Sci. Rep. 9:15789. doi: 10.1038/s41598-019-52422-z, PMID: 31673037 PMC6823512

[ref17] Li J. Dong L. Liu Y. Gao J. (2021). Stimulation of *codonopsis pilosula* polysaccharide on bifidobacterium of human gut bacteria *in vitro*. Evid. Based Complement. Alternat. Med. 2021:9524913. doi: 10.1155/2021/9524913, PMID: 33859715 PMC8024065

[ref18] Löffler F. E. Sun Q. Li J. Tiedje J. M. (2000). 16S rRNA gene-based detection of Tetrachloroethene-dechlorinating *Desulfuromonas* and *Dehalococcoides* species. Appl. Environ. Microbiol. 66, 1369–1374. doi: 10.1128/AEM.66.4.1369-1374.2000, PMID: 10742213 PMC91994

[ref19] McNichol J. Berube P. M. Biller S. J. Fuhrman J. A. (2021). Evaluating and improving small subunit rRNA PCR primer coverage for Bacteria, Archaea, and eukaryotes using metagenomes from Global Ocean surveys. mSystems 6:e0056521. doi: 10.1128/msystems.00565-21 34060911 PMC8269242

[ref20] Oligo Analyzer in IDT (n.d.). Available at: https://eu.idtdna.com/calc/analyzer (Accessed July 15, 2024).

[ref21] Parada A. E. Needham D. M. Fuhrman J. A. (2016). Every base matters: assessing small subunit rRNA primers for marine microbiomes with mock communities, time series and global field samples. Environ. Microbiol. 18, 1403–1414. doi: 10.1111/1462-2920.13023, PMID: 26271760

[ref22] Quast C. Pruesse E. Yilmaz P. Gerken J. Schweer T. Yarza P. . (2013). The SILVA ribosomal RNA gene database project: improved data processing and web-based tools. Nucleic Acids Res. 41, D590–D596. doi: 10.1093/nar/gks1219, PMID: 23193283 PMC3531112

[ref23] Quince C. Lanzen A. Davenport R. J. Turnbaugh P. J. (2011). Removing noise from Pyrosequenced amplicons. BMC Bioinformatics 12:38. doi: 10.1186/1471-2105-12-38, PMID: 21276213 PMC3045300

[ref24] Regueira-Iglesias A. Vázquez-González L. Balsa-Castro C. Vila-Blanco N. Blanco-Pintos T. Tamames J. . (2023). *In silico* evaluation and selection of the best 16S rRNA gene primers for use in next-generation sequencing to detect oral bacteria and archaea. Microbiome 11:58. doi: 10.1186/s40168-023-01481-6, PMID: 36949474 PMC10035280

[ref25] Schirmer M. Ijaz U. Z. D’Amore R. Hall N. Sloan W. T. Quince C. (2015). Insight into biases and sequencing errors for amplicon sequencing with the Illumina MiSeq platform. Nucleic Acids Res. 43:e37. doi: 10.1093/nar/gku1341, PMID: 25586220 PMC4381044

[ref26] Silva (n.d.). Available at: https://www.arb-silva.de (Accessed July 15, 2023).

[ref27] Sims D. Sudbery I. Ilott N. E. Heger A. Ponting C. P. (2014). Sequencing depth and coverage: key considerations in genomic analyses. Nat. Rev. Genet. 15, 121–132. doi: 10.1038/nrg3642 24434847

[ref28] Takahashi S. Tomita J. Nishioka K. Hisada T. Nishijima M. (2014). Development of a prokaryotic universal primer for simultaneous analysis of Bacteria and Archaea using next-generation sequencing. PLoS One 9:e105592. doi: 10.1371/journal.pone.0105592, PMID: 25144201 PMC4140814

[ref29] Walters W. A. Caporaso J. G. Lauber C. L. Berg-Lyons D. Fierer N. Knight R. (2011). PrimerProspector: De novo design and taxonomic analysis of barcoded polymerase chain reaction primers. Bioinformatics 27, 1159–1161. doi: 10.1093/bioinformatics/btr087, PMID: 21349862 PMC3072552

[ref1000] Yan J. Im J. Yang Y. Löffler F. E. (2013). Guided cobalamin biosynthesis supports dehalococcoides mccartyi reductive dechlorination activity. Philosophical Transactions of the Royal Society B: Biological Sciences 150. doi: 10.1098/rstb.2012.0320, PMID: 23479750 PMC3638461

[ref30] Yang Y. Mccarty P. L. (1998). Competition for hydrogen within a chlorinated solvent Dehalogenating anaerobic mixed culture. Environ. Sci. Technol. 32, 3591–3597. doi: 10.1021/es980363n

