# Supplementary material for: Developing the script “degenerate primer 111” to enhance the coverage of universal primers for the small subunit rRNA gene on target microorganisms
Source: Front Microbiol. 2024 Sep 4;15:1394303. doi: 10.3389/fmicb.2024.1394303 (PMC11409422; doi:10.3389/fmicb.2024.1394303)
Supplement: Supplementary file 1 [file Data_Sheet_1.zip › supporting/Supplementary Materials Revised-20240524.docx]

**Supplementary Materials for**

**“Developing the script "Degenerate primer 111" to enhance the coverage of universal primers for the small subunit rRNA gene on target microorganisms”**

**Contents:**

**Table S1** Primer coverage.

**Table S2** Improvements to commonly used primers to meet various detection needs.

**Table S1**

Primer coverage. “(V4)-1” refers to “515F-806R” in the manuscript, from the Earth Microbiome Project; “(V3-V4)-1” refers to “341F-785R” in the manuscript, from Illumina Inc.

| Primer | Sequence | Archaea | Bacteria | Eukaryota | Dehalococcoides |
| --- | --- | --- | --- | --- | --- |
| (V1-V3)-1 | 27F: AGAGTTTGATCCTGGCTCAG; | 0.00% | 57.20% | 0.00% | 95.80% |
|  | 534R：ATTACCGCGGCTGCTGG; |  |  |  |  |
| (V1-V3)-2 | 27F: AGAGTTTGATCMTGGCTCAG; | 0.00% | 71.80% | 0.00% | 95.80% |
|  | 519R: GWATTACCGCGGCKGCTG |  |  |  |  |
| (V1-V3)-3 | 27F: AGAGTTTGATCMTGGCTCAG; | 0.00% | 71.70% | 0.00% | 95.80% |
|  | 534R: ATTACCGCGGCTGCTGGC |  |  |  |  |
| (V3-V4)-1 | 341F: CCTACGGGNGGCWGCAG; | 0.30% | 81.20% | 0.00% | 5.30% |
|  | 785R: GACTACHVGGGTATCTAATCC |  |  |  |  |
| (V3-V4)-2 | 338f：ACTCCTACGGGAGGCAGCA; | 0.00% | 78.60% | 0.00% | 5.30% |
|  | 806r：GGACTACHVGGGTWTCTAAT |  |  |  |  |
| (V3-V4)-3 | 341F：CCTACGGGNBGCASCAG; | 67.80% | 81.00% | 0.00% | 5.30% |
|  | 805R：GACTACNVGGGTATCTAATCC; |  |  |  |  |
| (V3-V4)-4 | 341F：CCTAYGGGRBGCASCAG; | 79.10% | 79.80% | 0.00% | 5.30% |
|  | 805R：GGACTACNNGGGTATCTAAT; |  |  |  |  |
| (V3-V4)-5 | 341F：CCTAYGGGRBGCASCAG; | 67.20% | 79.70% | 0.00% | 5.30% |
|  | 806R: GGACTACNNGGGTATCTAAT; |  |  |  |  |
| (V3-V4)-6 | 341F：CCTACGGGRBGCASCAG; | 0.30% | 84.40% | 0.00% | 5.30% |
|  | 806R：GGACTACNNGGGTATCTAAT; |  |  |  |  |
| (V3-V4)-7 | 341F：CCTACGGGNGGCWGCAG; | 0.30% | 84.40% | 0.00% | 5.30% |
|  | 802R: TACNVGGGTATCTAATCC; |  |  |  |  |
| (V3-V4)-8 | 341F: CCTACGGGRSGCAGCAG; | 52.20% | 77.60% | 0.00% | 5.30% |
|  | 806R: GGACTACVVGGGTATCTAATC; |  |  |  |  |
| (V4)-1 | 515F: GTGYCAGCMGCCGCGGTAA; | 83.50% | 83.60% | 0.10% | 5.30% |
|  | 806R: GGACTACNVGGGTWTCTAAT; |  |  |  |  |
| (V4)-2 | 515F：GTGCCAGCMGCCGCGGTAA; | 49.80% | 8.50% | 0.00% | 0.00% |
|  | 806R：GGACTACVSGGGTATCTAAT; |  |  |  |  |
| (V4)-3 | 515F：GTGCCAGCMGCCGCGGTAA; | 51.40% | 82.90% | 0.06% | 5.30% |
|  | 806R：GGACTACHVGGGTWTCTAAT; |  |  |  |  |
| (V4)-4 | 515F：TGCCAGCMGCNGCGG; | 53.60% | 86.30% | 0.90% | 5.30% |
|  | 802R：TACNVGGGTATCTAATCC; |  |  |  |  |
| (V4-V5)-1 | 515F: GTGYCAGCMGCCGCGGTAA; | 81.00% | 84.50% | 80.80% | 92.10% |
|  | 926R: CCGYCAATTYMTTTRAGTTT; |  |  |  |  |
| (V4-V5)-2 | F: RGGATTAGATACCC; | 4.10% | 83.90% | 0.10% | 5.30% |
|  | R: CGACRRCCATGCANCACCT; |  |  |  |  |
| (V4-V5)-3 | 515F: GTGCCAGCMGCCGCGGTAA; | 47.80% | 81.10% | 0.10% | 92.10% |
|  | 909R: CCCCGYCAATTCMTTTRAGT; |  |  |  |  |
| (V4-V5)-4 | 515F: GTGCCAGCMGCCGCGG; | 0.60% | 82.60% | 69.60% | 92.10% |
|  | 907R：CCGTCAATTCMTTTRAGTTT; |  |  |  |  |
| (V9) | 926F: AAACTYAAAKGAATTGACGG; | 0.90% | 74.70% | 71.10% | 94.60% |
|  | 1392R: ACGGGCGGTGTGTRC; |  |  |  |  |

| Primer name | Reference |
| --- | --- |
| (V1-V3) -1 | (Matturro et al., 2020, 2021) |
| (V1-V3) -2 | (Lim et al., 2018) |
| (V1-V3) -3 | (Kao et al., 2016) |
| (V3-V4) -1 | (Quero et al., 2015; Yang et al., 2017; Xu et al., 2018; Blazquez-Palli et al., 2019; Luo et al., 2019; Chen et al., 2020; Grunert et al., 2020; Jing and Kjellerup, 2020; Lo et al., 2020; Tran et al., 2020) |
| (V3-V4) -2 | (Song et al., 2016, 2017; Liu et al., 2017; Wang et al., 2019; Jin et al., 2020; Guo et al., 2021) |
| (V3-V4) -3 | (Raju and Bidlan, 2017; Saghee and Bidlan, 2018; Laskar et al., 2019) |
| (V3-V4) -4 | (Feld et al., 2016; Holmsgaard et al., 2017; Wen et al., 2020) |
| (V3-V4) -5 | (Zhang et al., 2015; Yang et al., 2020) |
| (V3-V4) -6 | (Jiang et al., 2017) |
| (V3-V4) -7 | (Jiang et al., 2017) |
| (V3-V4) -8 | (Bao et al., 2020; Feng et al., 2021) |
| (V4) -1 | (Liang et al., 2017; Muturi et al., 2017; Brucha et al., 2021; Siggins et al., 2021) |
| (V4) -2 | (Liu et al., 2015, 2018; Chen et al., 2018b; Mattes et al., 2018) |
| (V4) -3 | (Chen et al., 2018a; Jiang et al., 2018; Li et al., 2018; Kaya et al., 2019; Ghezzi et al., 2021) |
| (V4) -4 | (Semerad et al., 2021) |
| (V4-V5) -1 | (Li et al., 2021) |
| (V4-V5) -2 | (Hellal et al., 2021) |
| (V4-V5) -3 | (Liang et al., 2017; Hermon et al., 2019; Mao et al., 2019; Lu et al., 2020) |
| (V4-V5) -4 | (Mirza et al., 2017; Jin et al., 2020; Xu et al., 2020) |
| (V9) | (Kocur et al., 2016; Jacome and Edwards, 2017; Qiao et al., 2018) |

**Table S2:** Improvements to commonly used primers to meet various detection needs. Modified bases are highlighted in bold.

| Primer Name | Target Microorganism | Forward Primer | Reverse Primer | Species Number | Species Coverage | | | | |
| --- | --- | --- | --- | --- | --- | --- | --- | --- | --- |
|  |  |  |  |  | Unimproved | Improved | Archaea | Bacteria | Eukaryote |
| BA-341F-806R |  | *CCTAYGGGRBGCASCAG* | *GGACTACNNGGGTATCTAAT* |  |  |  | 79.1% | 79.8% | 0.0% |
| BA-341F-806R-M1 | *Calescamantes* | *CCTAYGGGRBGCASCAG* | *GGACTACNNGGGT****M****TCTAAT* | 3 | 0.0% | 100.0% | 79.4% | 80.2% | 0.0% |
|  | *Aerophobota* |  |  | 70 | 2.9% | 88.6% |  |  |  |
|  | *Deferrisomatota* |  |  | 26 | 0.0% | 88.5% |  |  |  |
| BA-341F-806R-M2 | *Fermentibacterota* | *CCT****W****YGGGRBGCASCAG* | *GGACTA****Y****NNGGGTATCTAAT* | 54 | 0.0% | 81.5% | 79.4% | 79.9% | 0.0% |
| BA-341F-806R-M3 | *Asgardarchaeota* | *C****Y****TAYGGGRBGCASCAG* | *GGACTACNNGGGTATCTAAT* | 670 | 3.7% | 4.3% | 79.4% | 80.0% | 0.0% |
| BA-341F-806R-M4 | *Iainarchaeota* | *CCTAYGGGRBGCASCAG* | *GGACTA****M****NNGGGTATCTAAT* | 46 | 7.3% | 85.4% | 79.4% | 79.9% | 0.0% |
| A-784F-1059R |  | *GGMTTAGATACCC* | *GGCCATGCACCWCCTCTC* |  |  |  | 76.2% | 0.0% | 0.0% |
| A-784F-1059R-M1 | *Nanohaloarchaeota* | *G****R****MTTAGAT****W****CCC* | *GGCCA****Y****GCA****V****CWCCTCTC* | 34 | 0.0% | 100.0% | 77.5% | 0.0% | 0.0% |
| A-784F-1059R-M2 | *Korarchaeota* | *GGMTTAGATACCC* | *GGCCA****Y****GCACCWCC****Y****CTC* | 58 | 1.7% | 87.9% | 77.1% | 0.0% | 0.0% |
| A-784F-1059R-M3 | *Micrarchaeota* | ***R****G****VKY****AGATACCC* | ***S****GCCATGCA****VY****WC****Y****TCTC* | 54 | 1.9% | 96.3% | 85.1% | 0.0% | 0.0% |
| A-784F-1059R-M4 | *Altiarchaeota* | *GGMT****Y****AGATA****Y****CC* | *GGCCATGCAC****Y****WCC****Y****CTC* | 36 | 2.8% | 88.9% | 80.8% | 0.0% | 0.0% |
| BAE-515F-926R |  | *GTGYCAGCMGCCGCGGTAA* | *CCGYCAATTYMTTTRAGTTT* |  |  |  | 81.0% | 84.5% | 80.8% |
| BAE-515F-926R-M1 | *Excavata* | *GTGYCAGCMGC****Y****GCGGTAA* | *CCGYCAATTYMTT****Y****RAGTTT* | 383 | 6.0% | 90.1% | 81.3% | 84.7% | 82.9% |
| BAE-515F-926R-M2 | *Discoba* | *GTGYCAGCM****S****C****Y****GC****S****GTAA* | *CCGYCAATTYMTTTRAGTTT* | 979 | 9.1% | 85.6% | 81.1% | 84.8% | 83.8% |
| BAE-515F-926R-M3 | *Fervidibacteria* | *GTGYCAGCMGCCGCGGTAA* | *CCGYCAATT****BV****TTTRAGTTT* | 4 | 0.0% | 100.0% | 81.0% | 84.7% | 80.8% |
| BAE-515F-926R-M4 | *Poribacteria* | *GTGYCAGCMGC****Y****GCGGTAA* | *CCGYCAATT****H****M****Y****TTRAGTTT* | 52 | 0.0% | 76.9% | 81.7% | 84.9% | 81.9% |
| BAE-515F-926R-M5 | *Nanohaloarchaeota* | ***S****TGYCAGCMGCCGCGGTAA* | *CCGYCAATT****Y****M****T****TTRAGTTY* | 34 | 0.0% | 91.2% | 81.8% | 84.8% | 81.7% |
| BAE-515F-926R-M6 | *Altiarchaeota* | *GTGYCAGCM****K****CCGCGGTAA* | *CCGYCAATTYMTTTRAGTT****Y*** | 40 | 7.5% | 87.5% | 81.7% | 84.7% | 81.8% |
| E-616F-1132R | _ | *TTAAARVGYTCGTAGTYG* | *CCGTCAATTHCTTYAART* | _ | _ | _ | 80.7% | 0.0% | 0.0% |
| E-616F-1132R-M1 | Discoba | *TTAAARVGYTCGTAGTY****R*** | *CCGTCAATTHCTTYAART* | 1017 | 61.2% | 61.9% | 81.0% | 0.0% | 0.0% |
| E-616F-1132R-M2 | Excavata | *TTAAARVGY****Y****CGTAGTY* | *CCGTCAATTHCTTYAART* | 389 | 4.9% | 85.1% | 83.4% | 0.0% | 0.0% |

Bao, J., Wang, X., Gu, J., Dai, X., Zhang, K., Wang, Q., et al. (2020). Effects of macroporous adsorption resin on antibiotic resistance genes and the bacterial community during composting. *Bioresour Technol* 295, 121997. doi: 10.1016/j.biortech.2019.121997

Blazquez-Palli, N., Rosell, M., Varias, J., Bosch, M., Soler, A., Vicent, T., et al. (2019). Integrative isotopic and molecular approach for the diagnosis and implementation of an efficient in-situ enhanced biological reductive dechlorination of chlorinated ethenes. *Water Res* 167. doi: 10.1016/j.watres.2019.115106

Brucha, G., Aldas-Vargas, A., Ross, Z., Peng, P., Atashgahi, S., Smidt, H., et al. (2021). 2,4-Dichlorophenoxyacetic acid degradation in methanogenic mixed cultures obtained from Brazilian Amazonian soil samples. *Biodegradation* 32, 419–433. doi: 10.1007/s10532-021-09940-3

Chen, J., Wang, C., Pan, Y., Farzana, S. S., and Tam, N. F. Y. (2018a). Biochar accelerates microbial reductive debromination of 2,2″,4,4″-tetrabromodiphenyl ether (BDE-47) in anaerobic mangrove sediments. *J Hazard Mater* 341, 177–186. doi: 10.1016/j.jhazmat.2017.07.063

Chen, M., Tong, H., Li, F., Liu, C., Lan, Q., and Liu, C. (2018b). The effect of electron donors on the dechlorination of pentachlorophenol (PCP) and prokaryotic diversity in paddy soil. *Eur J Soil Biol* 86, 8–15. doi: 10.1016/j.ejsobi.2018.01.008

Chen, W. Y., Wu, J. H., and Chu, S. C. (2020). Deciphering microbiomes in anaerobic reactors with superior trichloroethylene dechlorination performance at low pH conditions. *Environmental Pollution* 257, 113567. doi: 10.1016/j.envpol.2019.113567

Feld, L., Nielsen, T. K., Hansen, L. H., Aamand, J., and Albers, C. N. (2016). Establishment of Bacterial Herbicide Degraders in a Rapid Sand Filter for Bioremediation of Phenoxypropionate-Polluted Groundwater. *Applied and Environmental Microbiology* 82, 878–887. doi: 10.1128/AEM.02600-15

Feng, Y., Duan, J. L., Sun, X. D., Ma, J. Y., Wang, Q., Li, X. Y., et al. (2021). Insights on the inhibition of anaerobic digestion performances under short-term exposure of metal-doped nanoplastics via Methanosarcina acetivorans. *Environmental Pollution* 275, 115755. doi: 10.1016/j.envpol.2020.115755

Ghezzi, D., Filippini, M., Cappelletti, M., Firrincieli, A., Zannoni, D., Gargini, A., et al. (2021). Molecular characterization of microbial communities in a peat-rich aquifer system contaminated with chlorinated aliphatic compounds. *Environmental Science and Pollution Research* 28, 23017–23035. doi: 10.1007/s11356-020-12236-3

Grunert, O., Hernandez-Sanabria, E., Buysens, S., De Neve, S., Van Labeke, M. C., Reheul, D., et al. (2020). In-Depth Observation on the Microbial and Fungal Community Structure of Four Contrasting Tomato Cultivation Systems in Soil Based and Soilless Culture Systems. *Front Plant Sci* 11, 1–22. doi: 10.3389/fpls.2020.520834

Guo, L., Wang, X., Lin, Y., Yang, X., Ni, K., and Yang, F. (2021). Microorganisms that are critical for the fermentation quality of paper mulberry silage. *Food Energy Secur*, 1–14. doi: 10.1002/fes3.304

Hellal, J., Joulian, C., Urien, C., Ferreira, S., Denonfoux, J., Hermon, L., et al. (2021). Chlorinated ethene biodegradation and associated bacterial taxa in multi-polluted groundwater: Insights from biomolecular markers and stable isotope analysis. *Science of the Total Environment* 763. doi: 10.1016/j.scitotenv.2020.142950

Hermon, L., Hellal, J., Denonfoux, J., Vuilleumier, S., Imfeld, G., Urien, C., et al. (2019). Functional genes and bacterial communities during organohalide respiration of chloroethenes in microcosms of multi-contaminated groundwater. *Front Microbiol* 10, 1–16. doi: 10.3389/fmicb.2019.00089

Holmsgaard, P. N., Dealtry, S., Dunon, V., Heuer, H., Hansen, L. H., Springael, D., et al. (2017). Response of the bacterial community in an on-farm biopurification system, to which diverse pesticides are introduced over an agricultural season. *Environ Pollut* 229, 854–862. doi: 10.1016/j.envpol.2017.07.026

Jacome, L. A. P., and Edwards, E. A. (2017). A switch of chlorinated substrate causes emergence of a previously undetected native Dehalobacter population in an established Dehalococcoides-dominated chloroethene-dechlorinating enrichment culture. *FEMS Microbiol Ecol* 93. doi: 10.1093/femsec/fix141

Jiang, L., Cheng, Z., Zhang, D., Song, M., Wang, Y., Luo, C., et al. (2017). The influence of e-waste recycling on the molecular ecological network of soil microbial communities in Pakistan and China. *Environ Pollut* 231, 173–181. doi: 10.1016/j.envpol.2017.08.003

Jiang, L., Luo, C., Zhang, D., Song, M., Sun, Y., and Zhang, G. (2018). Biphenyl-Metabolizing Microbial Community and a Functional Operon Revealed in E-Waste-Contaminated Soil. *Environ Sci Technol* 52, 8558–8567. doi: 10.1021/acs.est.7b06647

Jin, D., Zhang, F., Shi, Y., Kong, X., Xie, Y., Du, X., et al. (2020). Diversity of bacteria and archaea in the groundwater contaminated by chlorinated solvents undergoing natural attenuation. *Environ Res* 185, 109457. doi: 10.1016/j.envres.2020.109457

Jing, R., and Kjellerup, B. V. (2020). Predicting the potential for organohalide respiration in wastewater: Comparison of intestinal and wastewater microbiomes. *Science of the Total Environment* 705, 135833. doi: 10.1016/j.scitotenv.2019.135833

Kao, C. M., Liao, H. Y., Chien, C. C., Tseng, Y. K., Tang, P., Lin, C. E., et al. (2016). The change of microbial community from chlorinated solvent-contaminated groundwater after biostimulation using the metagenome analysis. *J Hazard Mater* 302, 144–150. doi: 10.1016/j.jhazmat.2015.09.047

Kaya, D., Kjellerup, B. V, Chourey, K., Hettich, R. L., Taggart, D. M., and Loffler, F. E. (2019). Impact of Fixed Nitrogen Availability on Dehalococcoides mccartyi Reductive Dechlorination Activity. *Environ Sci Technol* 53, 14548–14558. doi: 10.1021/acs.est.9b04463

Kocur, C. M. D., Lomheim, L., Molenda, O., Weber, K. P., Austrins, L. M., Sleep, B. E., et al. (2016). Long-Term Field Study of Microbial Community and Dechlorinating Activity Following Carboxymethyl Cellulose-Stabilized Nanoscale Zero-Valent Iron Injection. *Environ Sci Technol* 50, 7658–7670. doi: 10.1021/acs.est.6b01745

Laskar, M., Awata, T., Kasai, T., and Katayama, A. (2019). Anaerobic dechlorination by a humin-dependent pentachlorophenol-dechlorinating consortium under autotrophic conditions induced by homoacetogenesis. *Int J Environ Res Public Health* 16. doi: 10.3390/ijerph16162873

Li, J., Hu, A., Bai, S., Yang, X., Sun, Q., Liao, X., et al. (2021). Characterization and Performance of Lactate-Feeding Consortia for Reductive Dechlorination of Trichloroethene. *Microorganisms* 9. doi: 10.3390/microorganisms9040751

Li, Y., Li, X., Sun, Y., Zhao, X., and Li, Y. (2018). Cathodic microbial community adaptation to the removal of chlorinated herbicide in soil microbial fuel cells. *Environmental Science and Pollution Research* 25, 16900–16912. doi: 10.1007/s11356-018-1871-z

Liang, Y., Cook, L. J., and Mattes, T. E. (2017). Temporal abundance and activity trends of vinyl chloride (VC)-degrading bacteria in a dilute VC plume at Naval Air Station Oceana. *Environmental Science and Pollution Research* 24, 13760–13774. doi: 10.1007/s11356-017-8948-y

Lim, M. L., Brooks, M. D., Boothe, M. A., and Krzmarzick, M. J. (2018). Novel bacterial diversity is enriched with chloroperoxidase-reacted organic matter under anaerobic conditions. *FEMS Microbiol Ecol* 94. doi: 10.1093/femsec/fiy050

Liu, J., He, X., Lin, X., Chen, W., Zhou, Q., Shu, W., et al. (2015). Ecological Effects of Combined Pollution Associated with E-Waste Recycling on the Composition and Diversity of Soil Microbial Communities. *Environ Sci Technol* 49, 6438–6447. doi: 10.1021/es5049804

Liu, N., Li, H., Li, M., Ding, L., Weng, C. H., and Dong, C. Di (2017). Oxygen exposure effects on the dechlorinating activities of a trichloroethene-dechlorination microbial consortium. *Bioresour Technol* 240, 98–105. doi: 10.1016/j.biortech.2017.02.112

Liu, X., Wu, Y., Wilson, F. P., Yu, K., Lintner, C., Cupples, A. M., et al. (2018). Integrated methodological approach reveals microbial diversity and functions in aerobic groundwater microcosms adapted to vinyl chloride. *FEMS Microbiol Ecol* 94. doi: 10.1093/femsec/fiy124

Lo, K. H., Lu, C. W., Lin, W. H., Chien, C. C., Chen, S. C., and Kao, C. M. (2020). Enhanced reductive dechlorination of trichloroethene with immobilized Clostridium butyricum in silica gel. *Chemosphere* 238, 124596. doi: 10.1016/j.chemosphere.2019.124596

Lu, Q., Zou, X., Liu, J., Liang, Z., Shim, H., Qiu, R., et al. (2020). Inhibitory effects of metal ions on reductive dechlorination of polychlorinated biphenyls and perchloroethene in distinct organohalide-respiring bacteria. *Environ Int* 135, 105373. doi: 10.1016/j.envint.2019.105373

Luo, S. G., Chen, S. C., Cao, W. Z., Lin, W. H., Sheu, Y. T., and Kao, C. M. (2019). Application of Γ-PGA as the primary carbon source to bioremediate a TCE-polluted aquifer: A pilot-scale study. *Chemosphere* 237, 124449. doi: 10.1016/j.chemosphere.2019.124449

Mao, X., Stenuit, B., Tremblay, J., Yu, K., Tringe, S. G., and Alvarez-Cohen, L. (2019). Structural dynamics and transcriptomic analysis of Dehalococcoides mccartyi within a TCE-Dechlorinating community in a completely mixed flow reactor. *Water Res* 158, 146–156. doi: 10.1016/j.watres.2019.04.038

Mattes, T. E., Ewald, J. M., Liang, Y., Martinez, A., Awad, A., Richards, P., et al. (2018). PCB dechlorination hotspots and reductive dehalogenase genes in sediments from a contaminated wastewater lagoon. *Environmental Science and Pollution Research* 25, 16376–16388. doi: 10.1007/s11356-017-9872-x

Matturro, B., Majone, M., Aulenta, F., and Rossetti, S. (2021). Correlations between maximum reductive dechlorination rates and specific biomass parameters in Dehalococcoides mccartyi consortia enriched on chloroethenes PCE, TCE and cis-1,2-DCE. *FEMS Microbiol Ecol* 97. doi: 10.1093/femsec/fiab064

Matturro, B., Mascolo, G., and Rossetti, S. (2020). Microbiome changes and oxidative capability of an anaerobic PCB dechlorinating enrichment culture after oxygen exposure. *N Biotechnol* 56, 96–102. doi: 10.1016/j.nbt.2019.12.004

Mirza, B. S., Sorensen, D. L., McGlinn, D. J., Dupont, R. R., and McLean, J. E. (2017). Dehalococcoides and general bacterial ecology of differentially trichloroethene dechlorinating flow-through columns. *Appl Microbiol Biotechnol* 101, 4799–4813. doi: 10.1007/s00253-017-8180-1

Muturi, E. J., Donthu, R. K., Fields, C. J., Moise, I. K., and Kim, C.-H. (2017). Effect of pesticides on microbial communities in container aquatic habitats. *Sci Rep* 7. doi: 10.1038/srep44565

Qiao, W., Luo, F., Lomheim, L., Mack, E. E., Ye, S., Wu, J., et al. (2018). A Dehalogenimonas Population Respires 1,2,4-Trichlorobenzene and Dichlorobenzenes. *Environ Sci Technol* 52, 13391–13398. doi: 10.1021/acs.est.8b04239

Quero, G. M., Cassin, D., Botter, M., Perini, L., and Luna, G. M. (2015). Patterns of benthic bacterial diversity in coastal areas contaminated by heavy metals, polycyclic aromatic hydrocarbons (PAHs) and polychlorinated biphenyls (PCBs). *Front Microbiol* 6, 1053. doi: 10.3389/fmicb.2015.01053

Raju, S. M., and Bidlan, R. (2017). 16S metagenomic analysis and taxonomic distribution of enriched microbial consortia capable of simultaneous biodegradation of organochlorines by illumina platform. *Biosci Biotechnol Res Commun* 10, 697–703. doi: 10.21786/bbrc/10.4/13

Saghee, M. R., and Bidlan, R. (2018). Simultaneous degradation of organochlorine pesticides by microbial consortium. *Biosci Biotechnol Res Commun* 11, 49–54. doi: 10.21786/bbrc/11.1/7

Semerad, J., Sevcu, A., Nguyen, N. H. A., Hrabak, P., Spanek, R., Bobcikova, K., et al. (2021). Discovering the potential of an nZVI-biochar composite as a material for the nanobioremediation of chlorinated solvents in groundwater: Degradation efficiency and effect on resident microorganisms. *Chemosphere* 281. doi: 10.1016/j.chemosphere.2021.130915

Siggins, A., Thorn, C., Healy, M. G., and Abram, F. (2021). Simultaneous adsorption and biodegradation of trichloroethylene occurs in a biochar packed column treating contaminated landfill leachate. *J Hazard Mater* 403, 123676. doi: 10.1016/j.jhazmat.2020.123676

Song, Y., Bian, Y., Wang, F., Herzberger, A., Yang, X., Gu, C., et al. (2017). Effects of biochar on dechlorination of hexachlorobenzene and the bacterial community in paddy soil. *Chemosphere* 186, 116–123. doi: 10.1016/j.chemosphere.2017.07.139

Song, Y., Li, Y., Zhang, W., Wang, F., Bian, Y., Boughner, L. A., et al. (2016). Novel Biochar-Plant Tandem Approach for Remediating Hexachlorobenzene Contaminated Soils: Proof-of-Concept and New Insight into the Rhizosphere. *J Agric Food Chem* 64, 5464–5471. doi: 10.1021/acs.jafc.6b01035

Tran, H. T., Lin, C., Hoang, H. G., Nguyen, M. T., Kaewlaoyoong, A., Cheruiyot, N. K., et al. (2020). Biodegradation of dioxin-contaminated soil via composting: Identification and phylogenetic relationship of bacterial communities. *Environ Technol Innov* 19, 101023. doi: 10.1016/j.eti.2020.101023

Wang, Q., Cao, Z., Liu, Q., Zhang, J., Hu, Y., Zhang, J., et al. (2019). Enhancement of COD removal in constructed wetlands treating saline wastewater: Intertidal wetland sediment as a novel inoculation. *J Environ Manage* 249, 109398. doi: 10.1016/j.jenvman.2019.109398

Wen, L. L., Li, Y., Zhu, L., and Zhao, H. P. (2020). Influence of non-dechlorinating microbes on trichloroethene reduction based on vitamin B12 synthesis in anaerobic cultures. *Environmental Pollution* 259, 113947. doi: 10.1016/j.envpol.2020.113947

Xu, H., Zhao, X., Huang, S., Li, H., Tong, N., Wen, X., et al. (2018). Evaluation of microbial p-chloroaniline degradation in bioelectrochemical reactors in the presence of easily-biodegrading cosubstrates: Degradation efficiency and bacterial community structure. *Bioresour Technol* 270, 422–429. doi: 10.1016/j.biortech.2018.09.064

Xu, Y., Teng, Y., Wang, X., Li, R., and Christie, P. (2020). Exploring bacterial community structure and function associated with polychlorinated biphenyl biodegradation in two hydrogen-amended soils. *Science of the Total Environment* 745, 140839. doi: 10.1016/j.scitotenv.2020.140839

Yang, Y., Higgins, S. A., Yan, J., Simsir, B., Chourey, K., Iyer, R., et al. (2017). Grape pomace compost harbors organohalide-respiring Dehalogenimonas species with novel reductive dehalogenase genes. *Isme Journal* 11, 2767–2780. doi: 10.1038/ismej.2017.127

Yang, Y., Zhang, Y., Capiro, N. L., and Yan, J. (2020). Genomic Characteristics Distinguish Geographically DistributedDehalococcoidia. *Front Microbiol* 11. doi: 10.3389/fmicb.2020.546063

Zhang, Y., Hu, M., Li, P., Wang, X., and Meng, Q. (2015). Analysis of trichloroethylene removal and bacterial community function based on pH-adjusted in an upflow anaerobic sludge blanket reactor. *Appl Microbiol Biotechnol* 99, 9289–9297. doi: 10.1007/s00253-015-6800-1
