# Supplementary figures and images for: Developing the script “degenerate primer 111” to enhance the coverage of universal primers for the small subunit rRNA gene on target microorganisms
Source: Front Microbiol. 2024 Sep 4;15:1394303. doi: 10.3389/fmicb.2024.1394303 (PMC11409422; doi:10.3389/fmicb.2024.1394303)

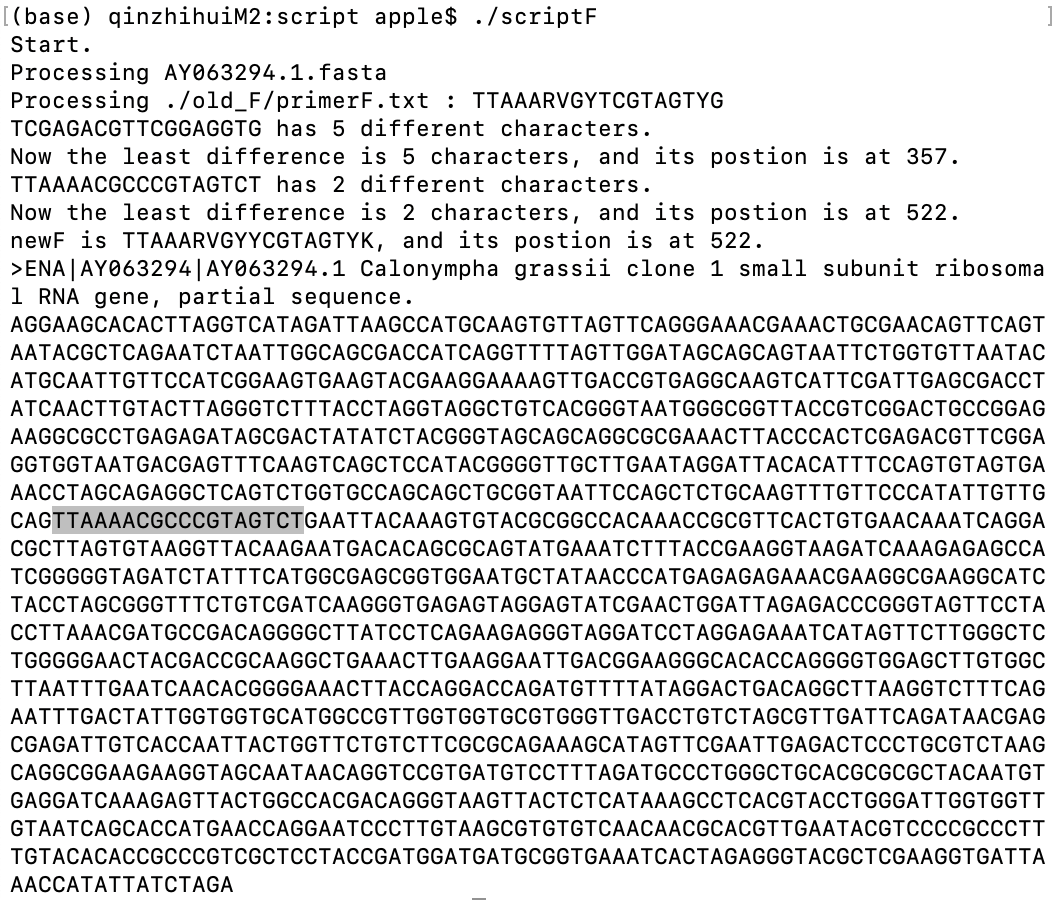

Supplement: Supplementary file 1 [file Data_Sheet_1.zip › supporting/Gene sequences and script execution results/E-616F-1132R and its improved primers/Excavata/E-616F-1132R-M2-F.png]

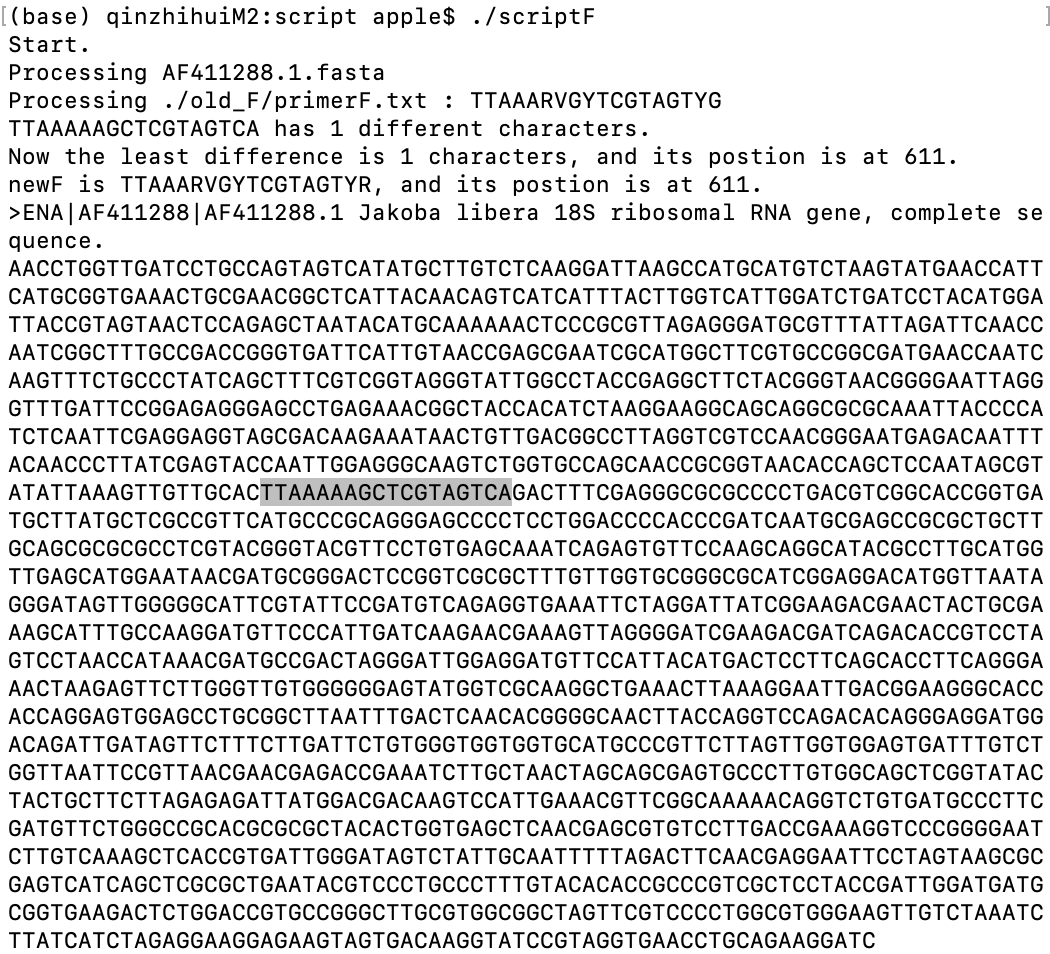

Supplement: Supplementary file 1 [file Data_Sheet_1.zip › supporting/Gene sequences and script execution results/E-616F-1132R and its improved primers/Discoba/E-616F-1132R-M1-F.png]

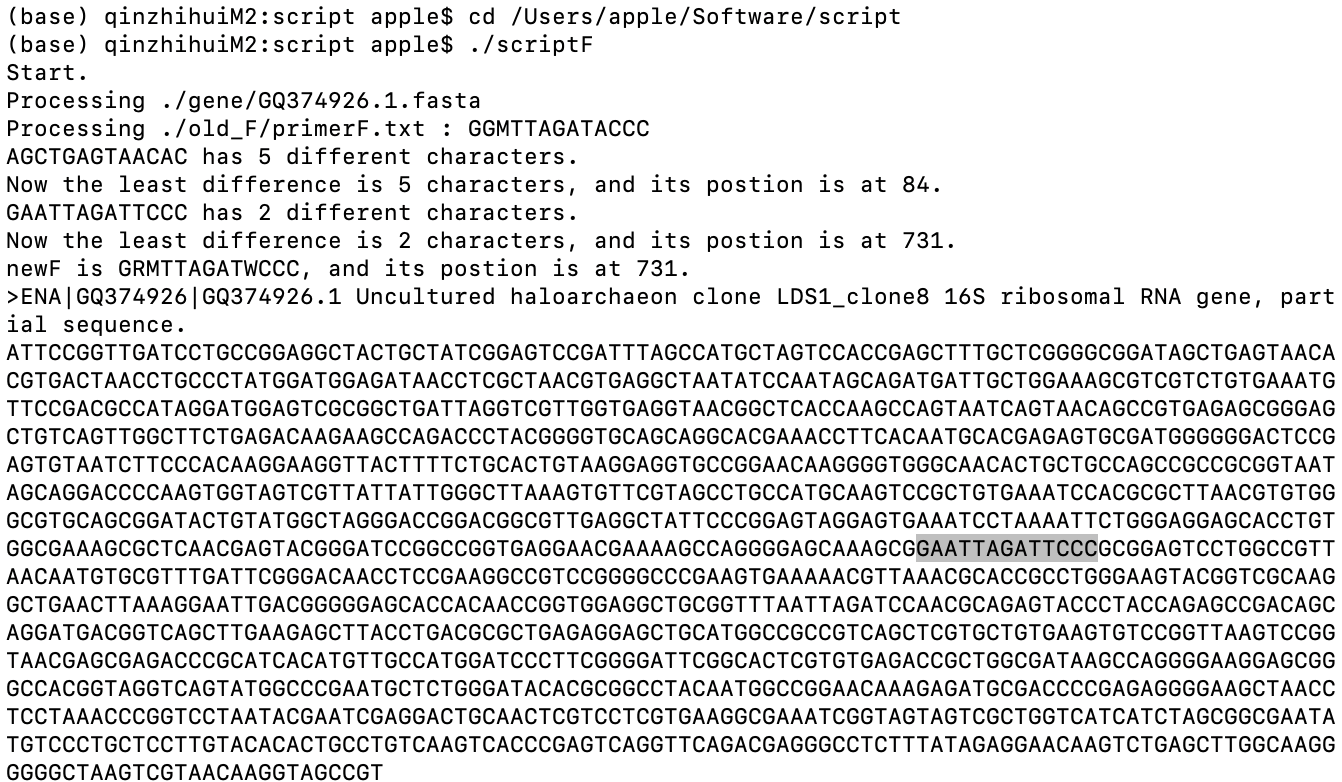

Supplement: Supplementary file 1 [file Data_Sheet_1.zip › supporting/Gene sequences and script execution results/A-784F-1059R and its improved primers/Nanohaloarchaeota/Archaea-784F-1059R-M1-F1.png]

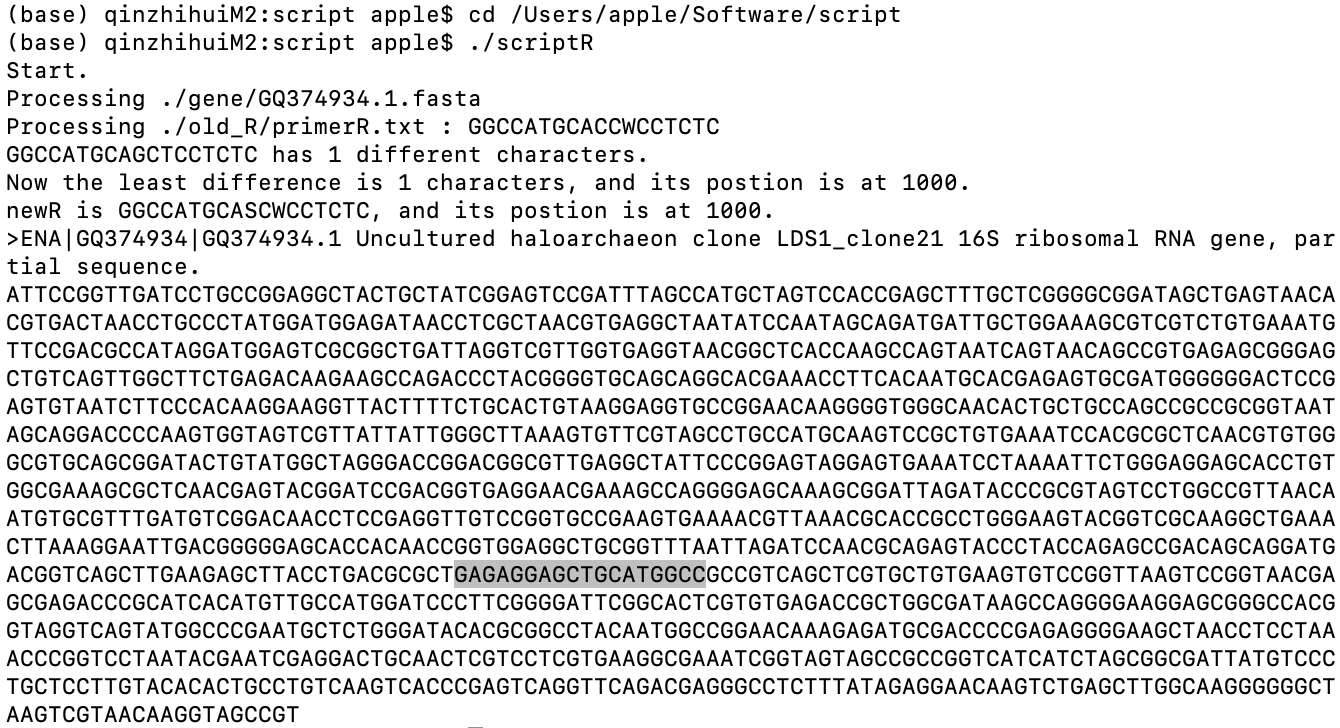

Supplement: Supplementary file 1 [file Data_Sheet_1.zip › supporting/Gene sequences and script execution results/A-784F-1059R and its improved primers/Nanohaloarchaeota/Archaea-784F-1059R-M1-R1.png]

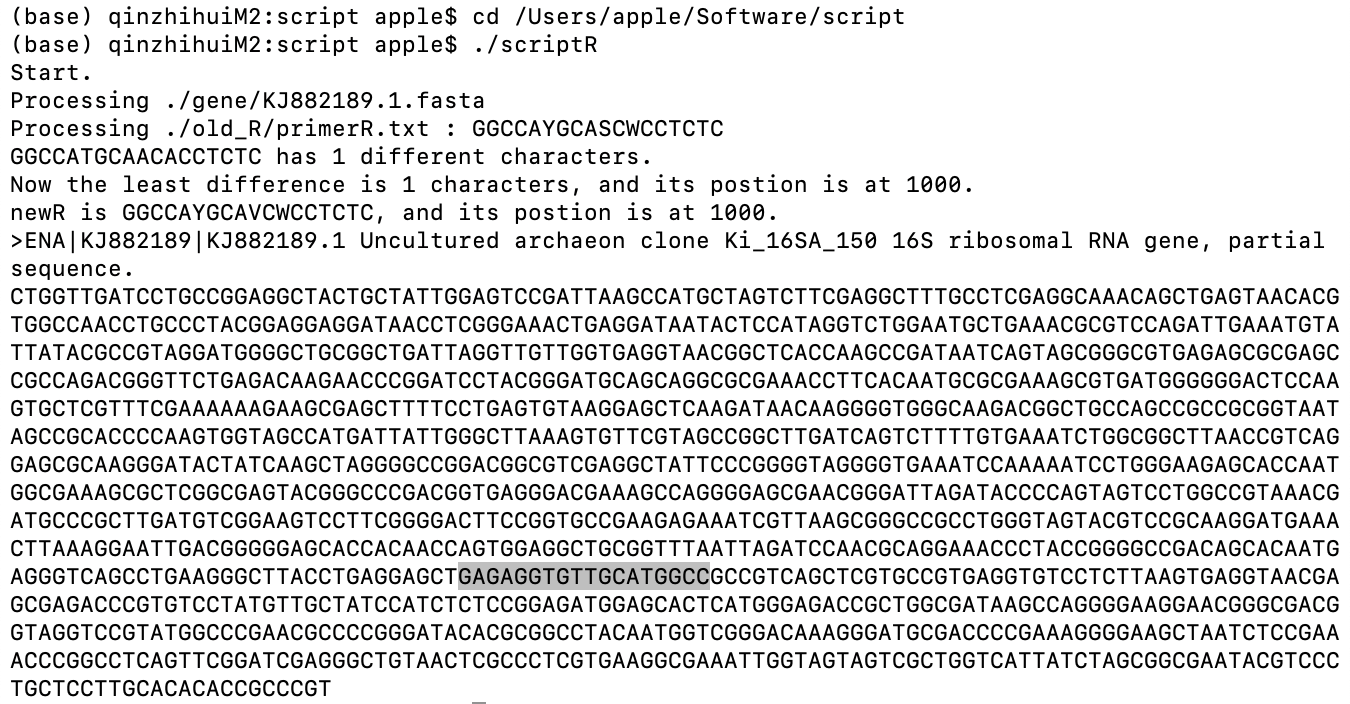

Supplement: Supplementary file 1 [file Data_Sheet_1.zip › supporting/Gene sequences and script execution results/A-784F-1059R and its improved primers/Nanohaloarchaeota/Archaea-784F-1059R-M1-R3.png]

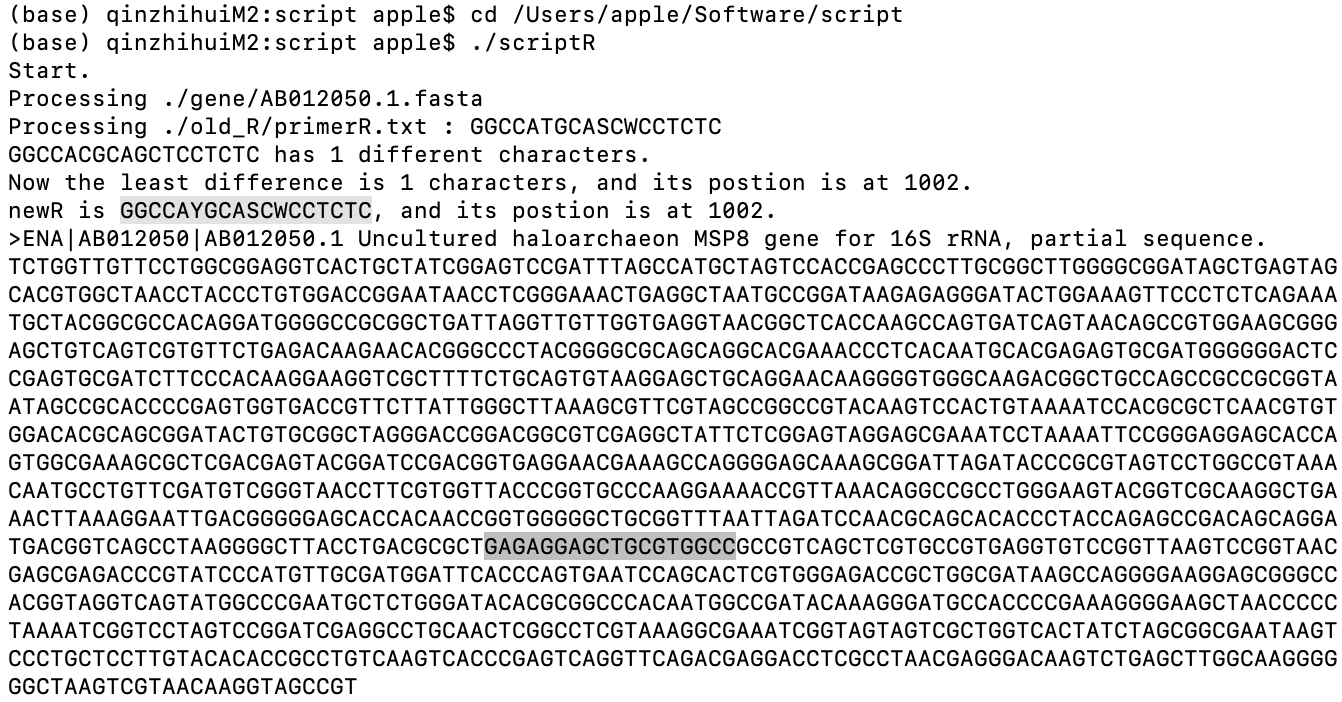

Supplement: Supplementary file 1 [file Data_Sheet_1.zip › supporting/Gene sequences and script execution results/A-784F-1059R and its improved primers/Nanohaloarchaeota/Archaea-784F-1059R-M1-R2.png]

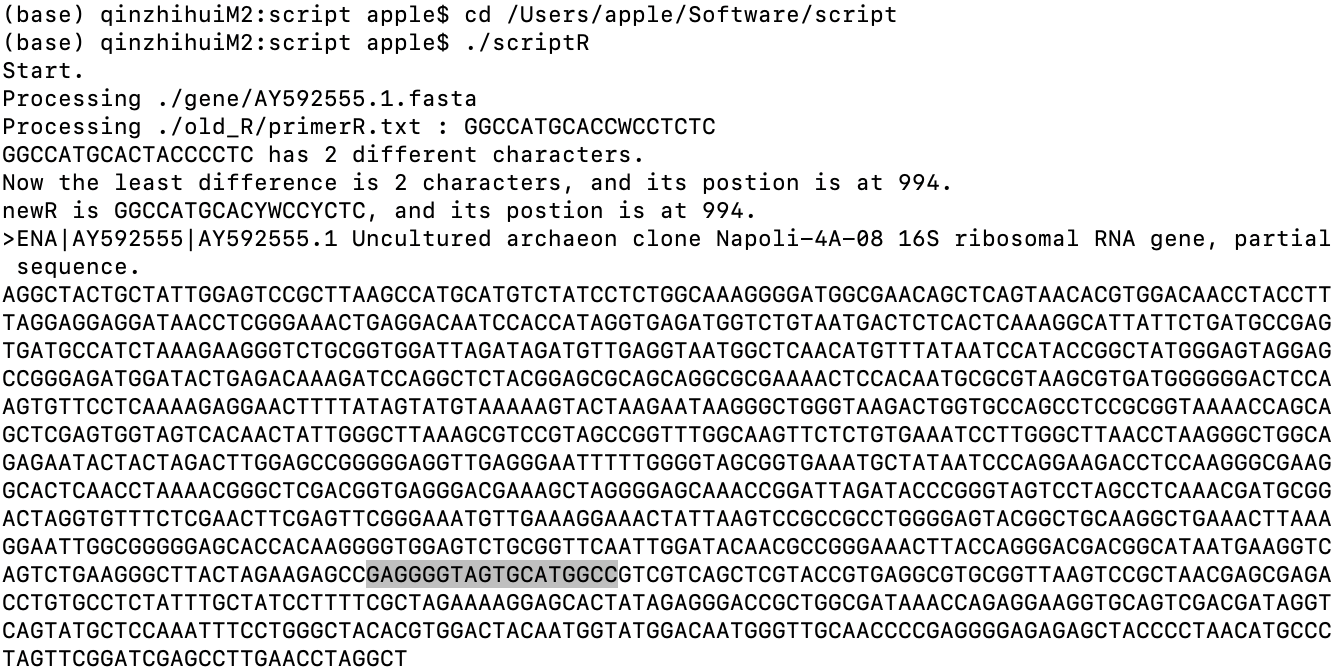

Supplement: Supplementary file 1 [file Data_Sheet_1.zip › supporting/Gene sequences and script execution results/A-784F-1059R and its improved primers/Altiarchaeota/Archaea-784F-1059R-M4-R2.png]

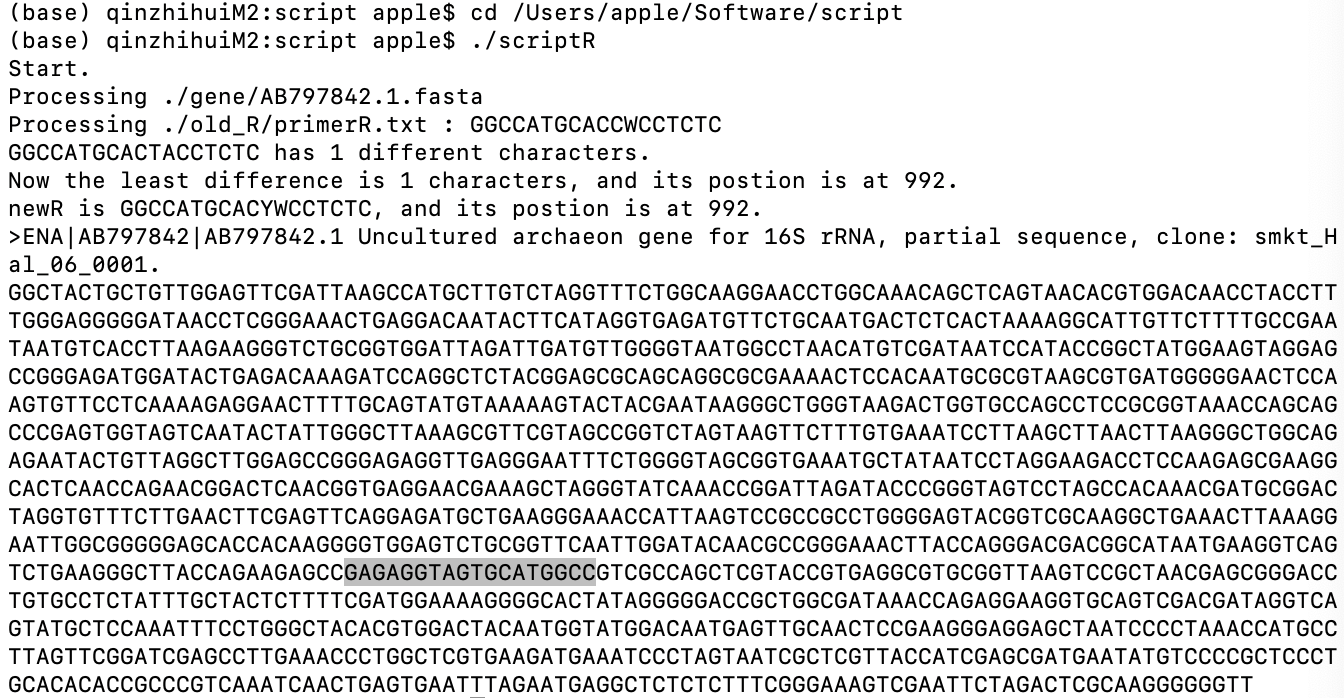

Supplement: Supplementary file 1 [file Data_Sheet_1.zip › supporting/Gene sequences and script execution results/A-784F-1059R and its improved primers/Altiarchaeota/Archaea-784F-1059R-M4-R1.png]

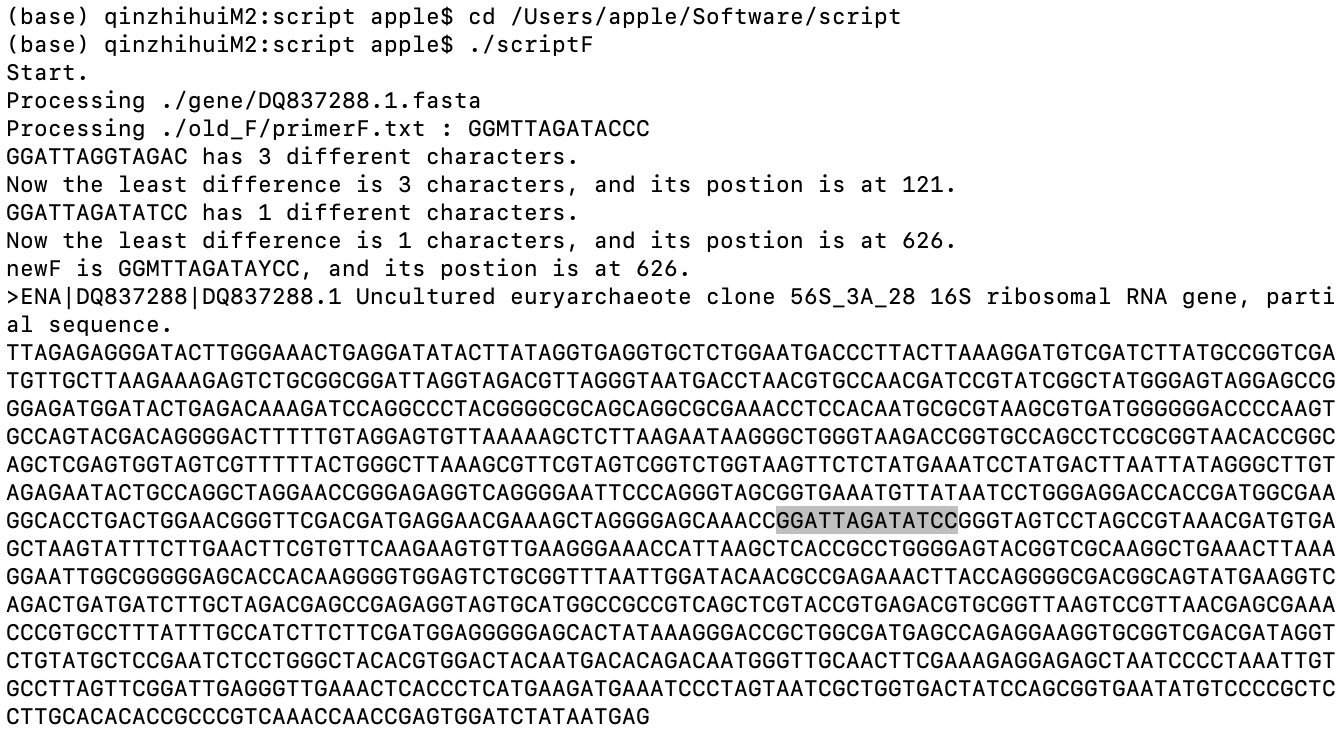

Supplement: Supplementary file 1 [file Data_Sheet_1.zip › supporting/Gene sequences and script execution results/A-784F-1059R and its improved primers/Altiarchaeota/Archaea-784F-1059R-M4-F1.png]

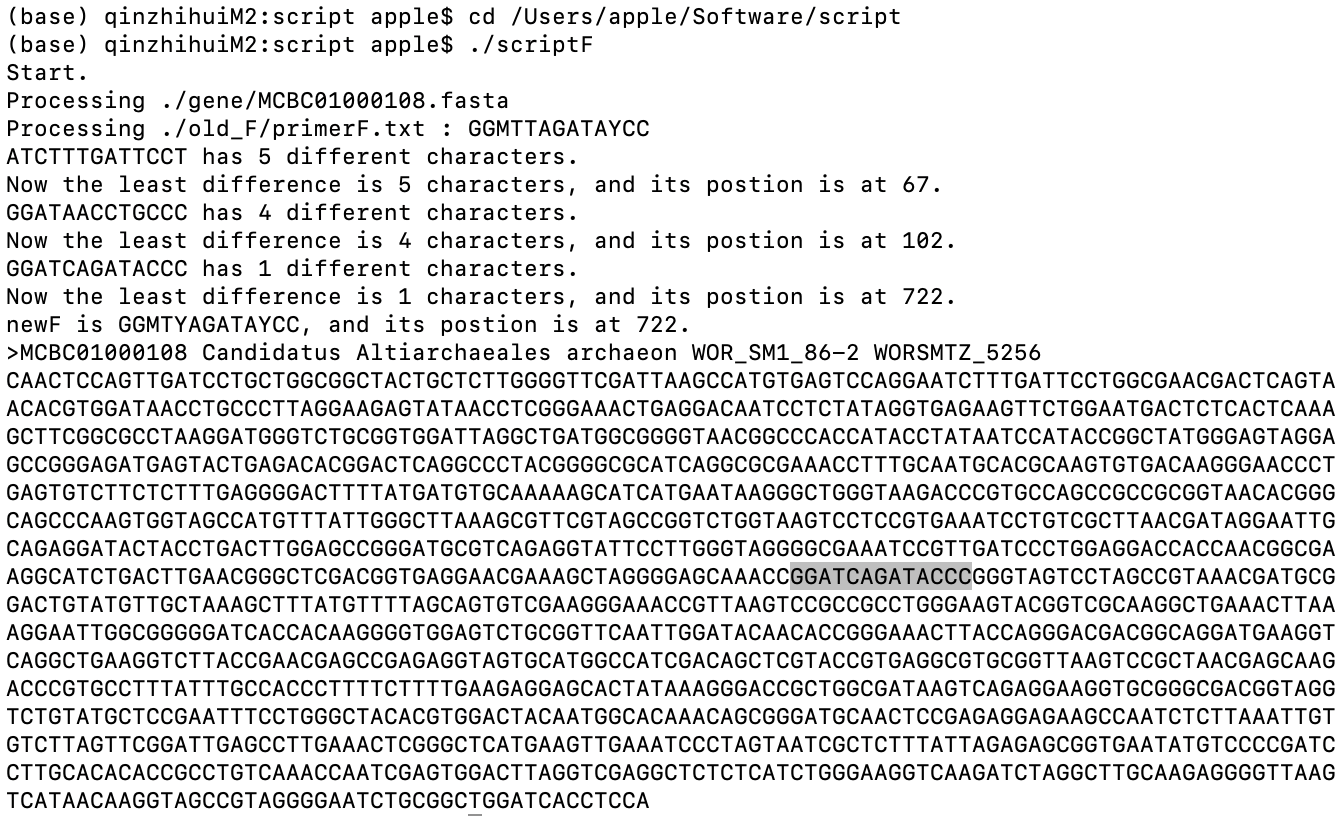

Supplement: Supplementary file 1 [file Data_Sheet_1.zip › supporting/Gene sequences and script execution results/A-784F-1059R and its improved primers/Altiarchaeota/Archaea-784F-1059R-M4-F2.png]

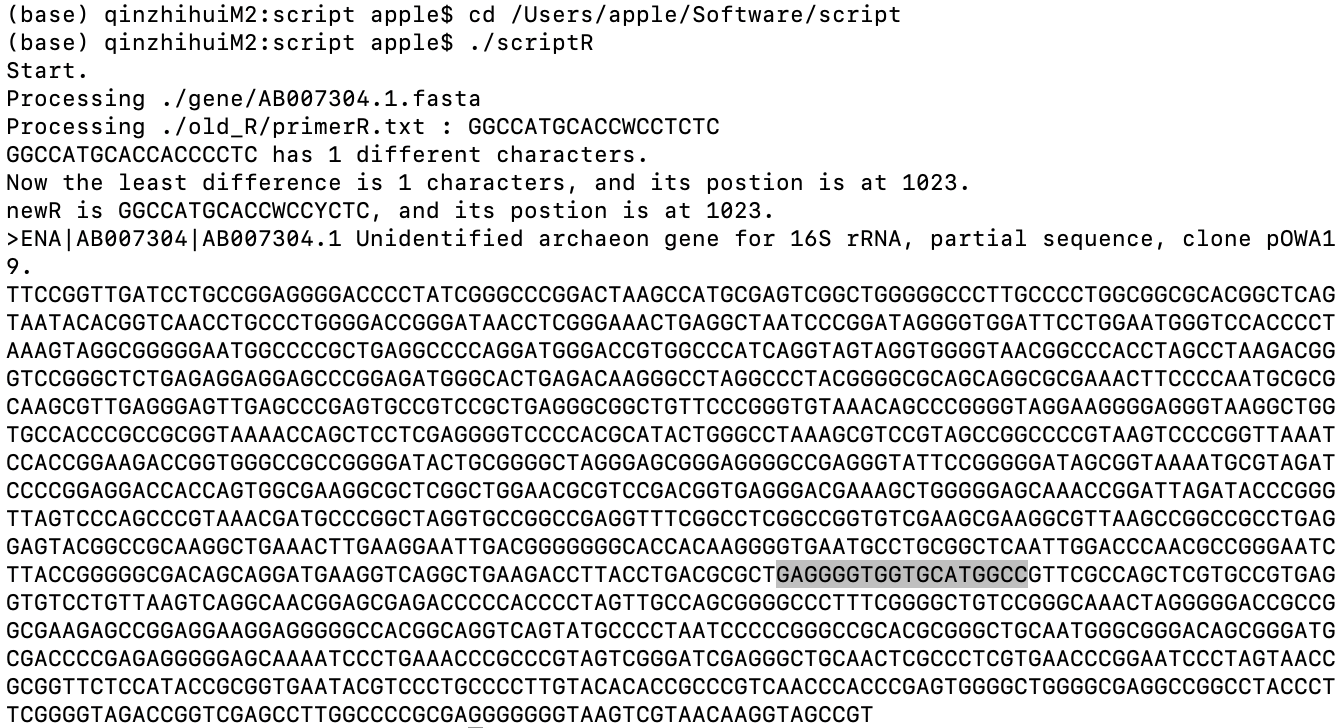

Supplement: Supplementary file 1 [file Data_Sheet_1.zip › supporting/Gene sequences and script execution results/A-784F-1059R and its improved primers/Korarchaeota/Archaea-784F-1059R-M2-R1.png]

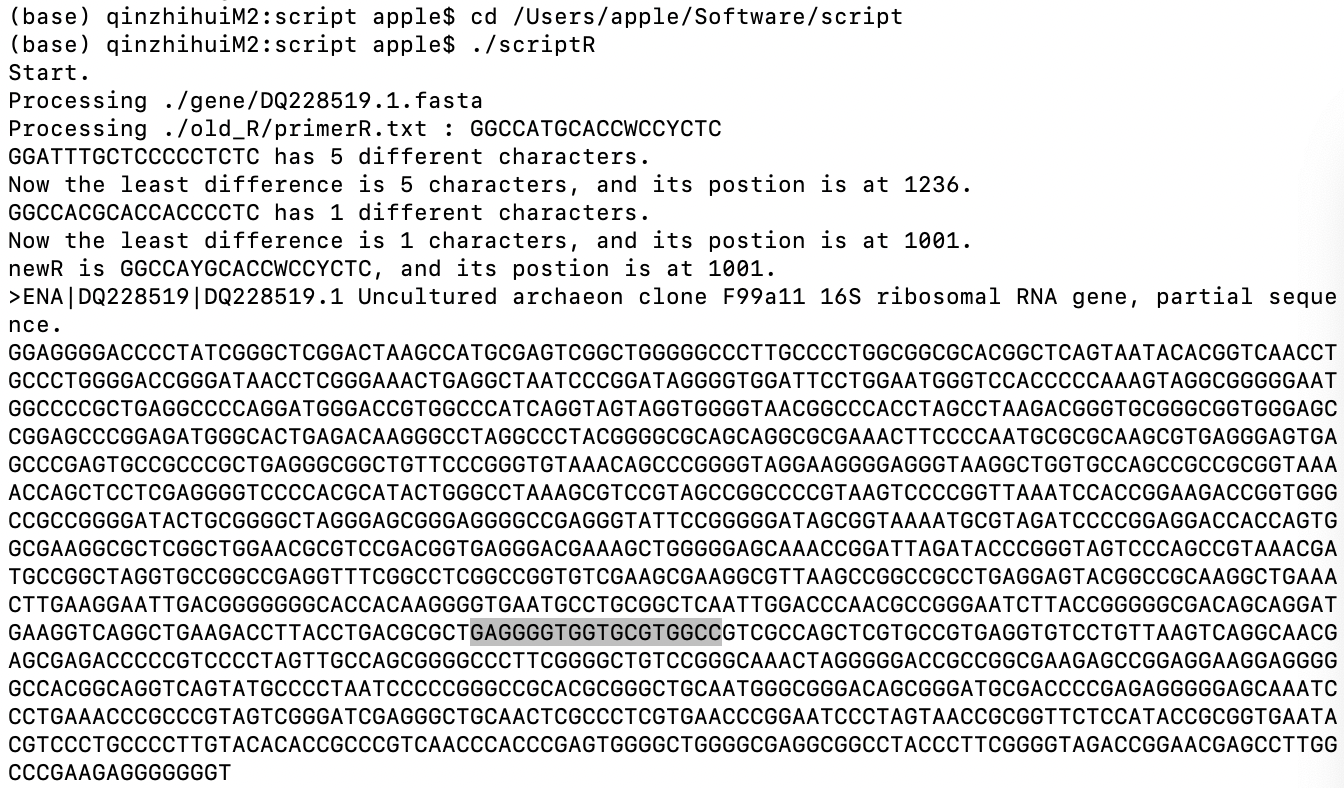

Supplement: Supplementary file 1 [file Data_Sheet_1.zip › supporting/Gene sequences and script execution results/A-784F-1059R and its improved primers/Korarchaeota/Archaea-784F-1059R-M2-R2.png]

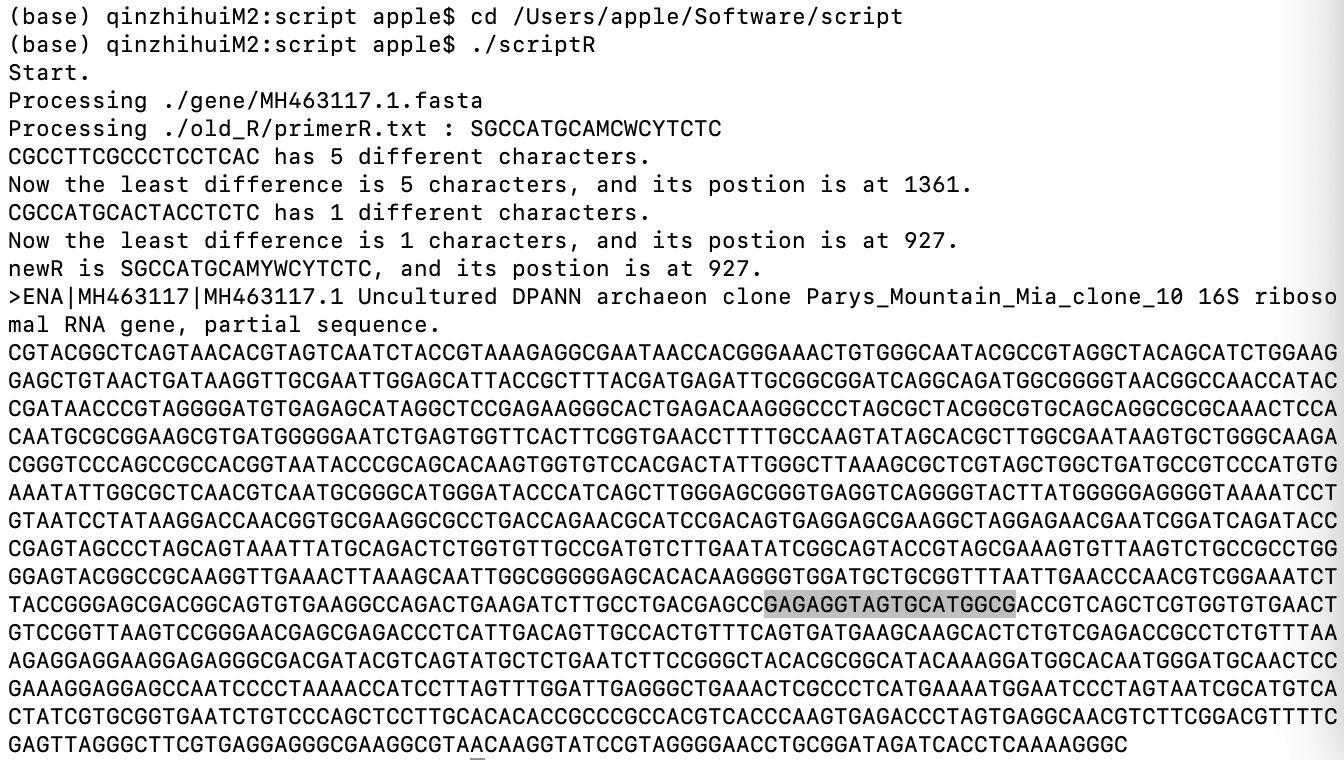

Supplement: Supplementary file 1 [file Data_Sheet_1.zip › supporting/Gene sequences and script execution results/A-784F-1059R and its improved primers/Micrarchaeota/Archaea-784F-1059R-M3-R3.png]

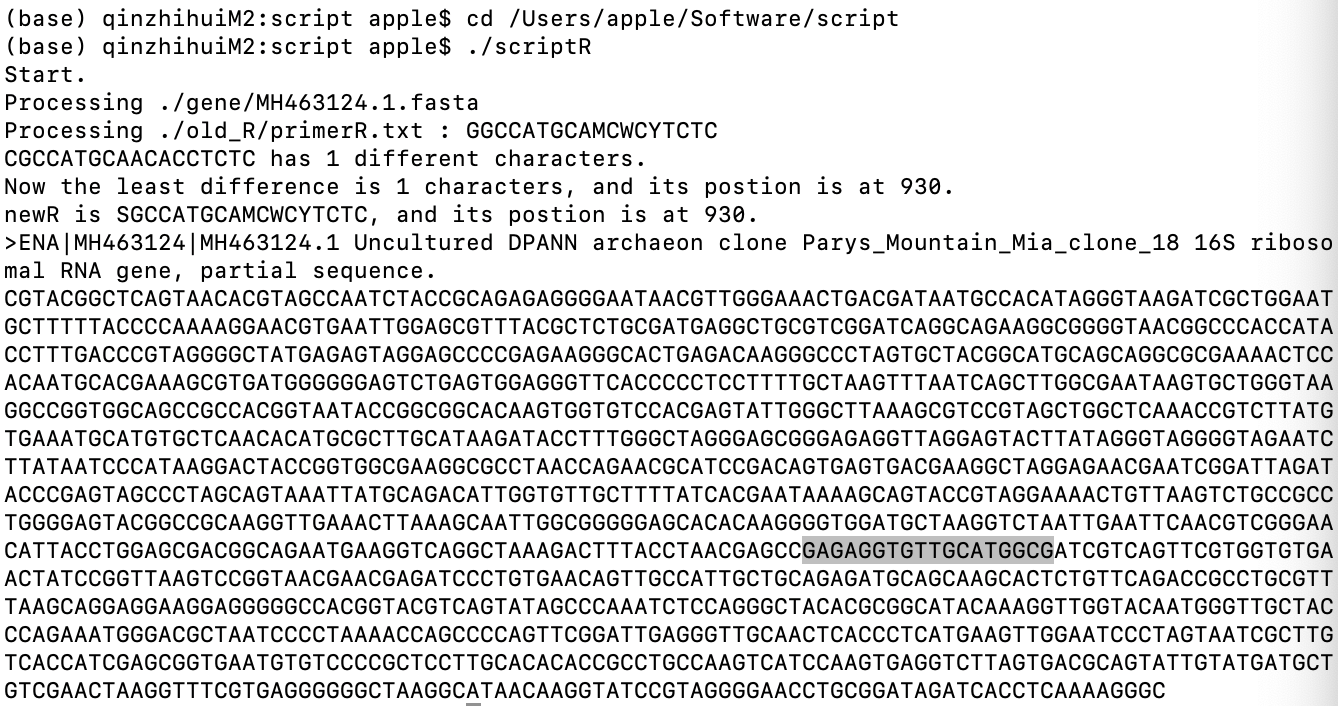

Supplement: Supplementary file 1 [file Data_Sheet_1.zip › supporting/Gene sequences and script execution results/A-784F-1059R and its improved primers/Micrarchaeota/Archaea-784F-1059R-M3-R2.png]

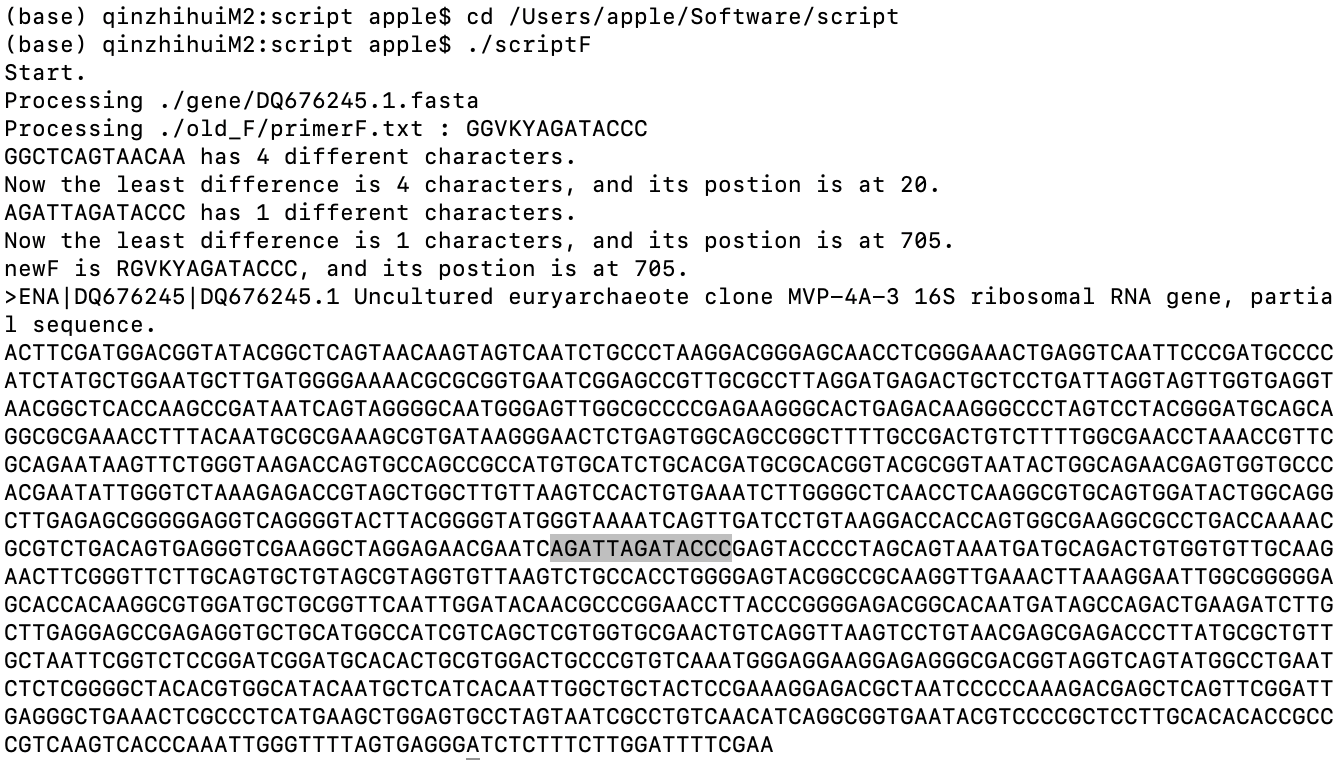

Supplement: Supplementary file 1 [file Data_Sheet_1.zip › supporting/Gene sequences and script execution results/A-784F-1059R and its improved primers/Micrarchaeota/Archaea-784F-1059R-M3-F4.png]

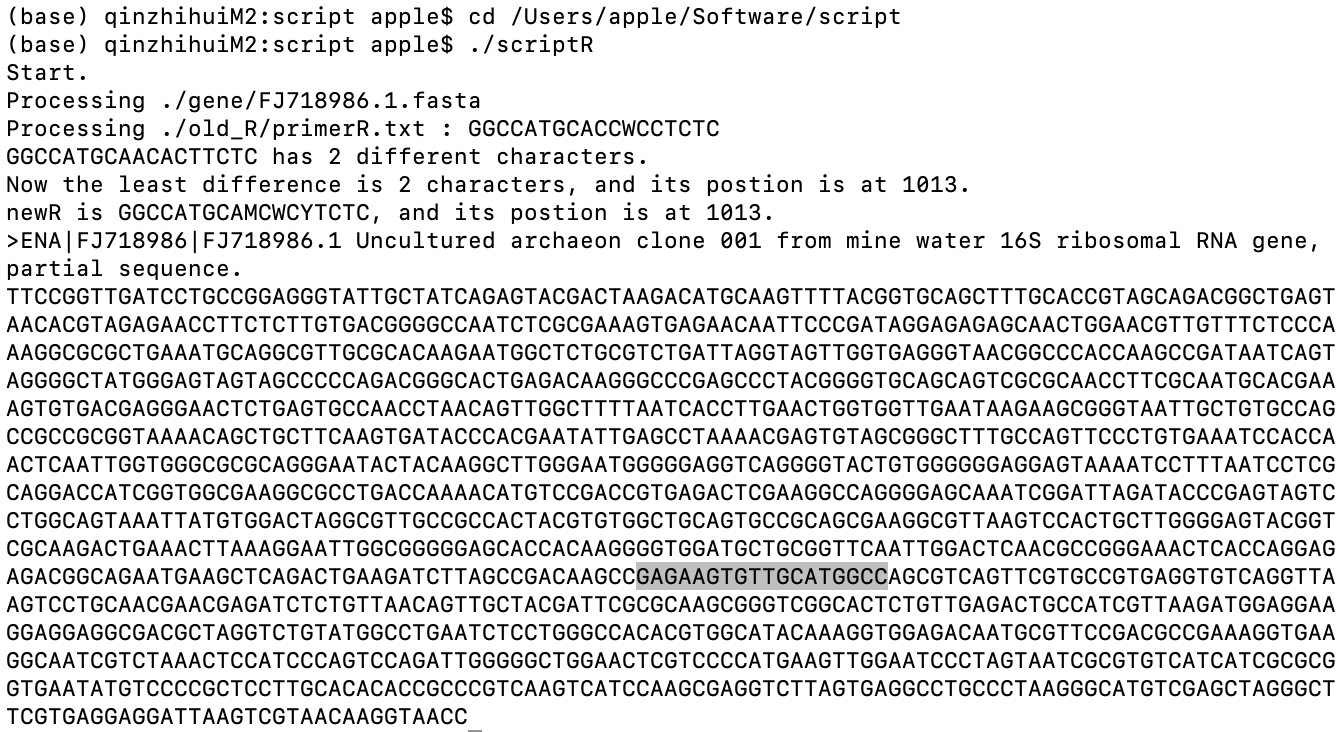

Supplement: Supplementary file 1 [file Data_Sheet_1.zip › supporting/Gene sequences and script execution results/A-784F-1059R and its improved primers/Micrarchaeota/Archaea-784F-1059R-M3-R1.png]

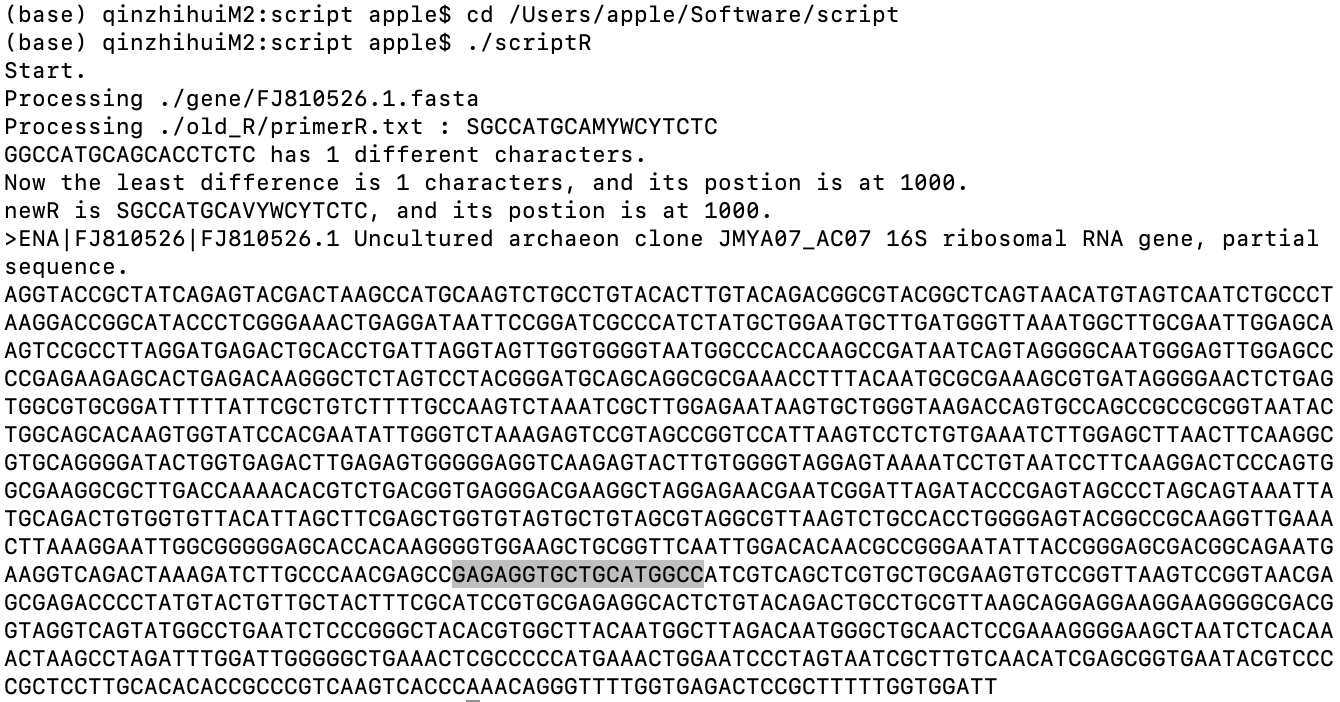

Supplement: Supplementary file 1 [file Data_Sheet_1.zip › supporting/Gene sequences and script execution results/A-784F-1059R and its improved primers/Micrarchaeota/Archaea-784F-1059R-M3-R5.png]

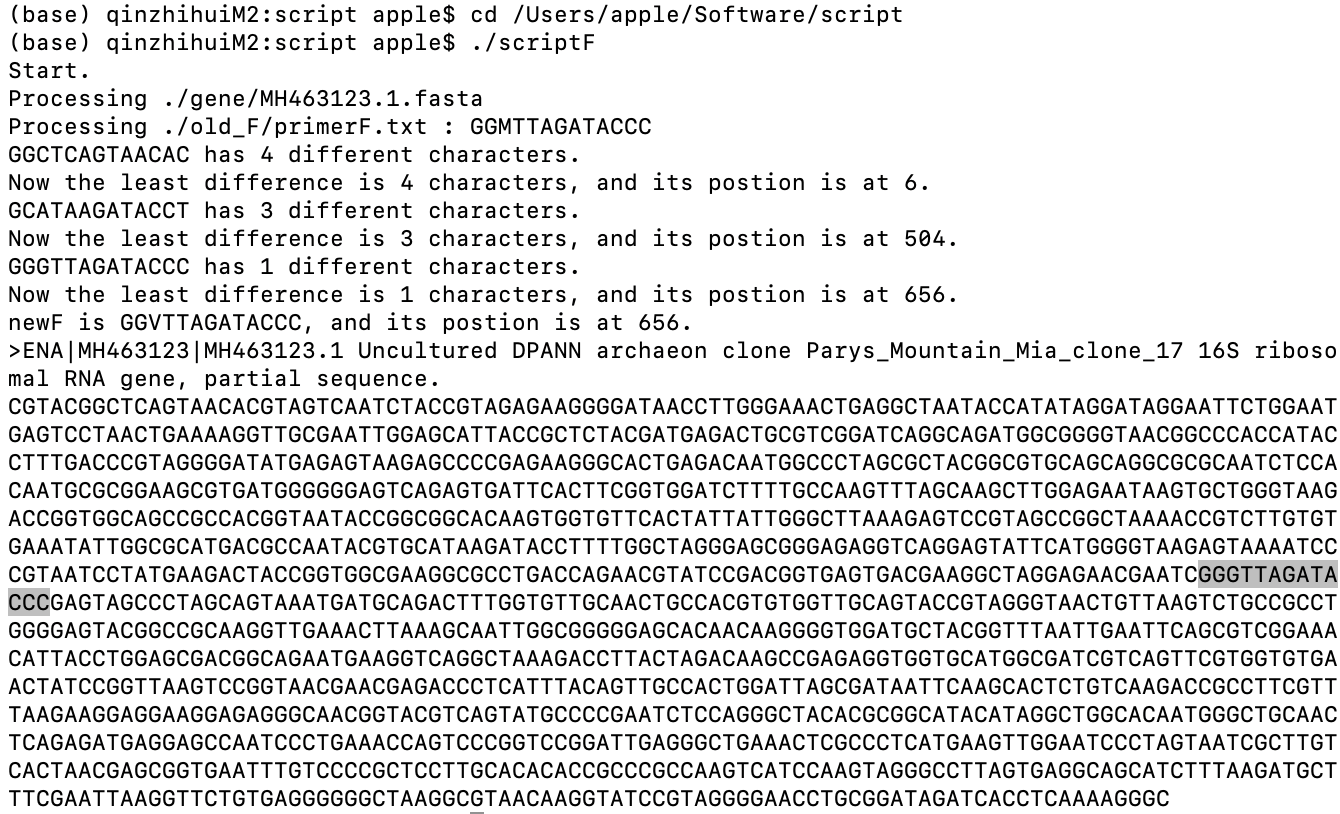

Supplement: Supplementary file 1 [file Data_Sheet_1.zip › supporting/Gene sequences and script execution results/A-784F-1059R and its improved primers/Micrarchaeota/Archaea-784F-1059R-M3-F1.png]

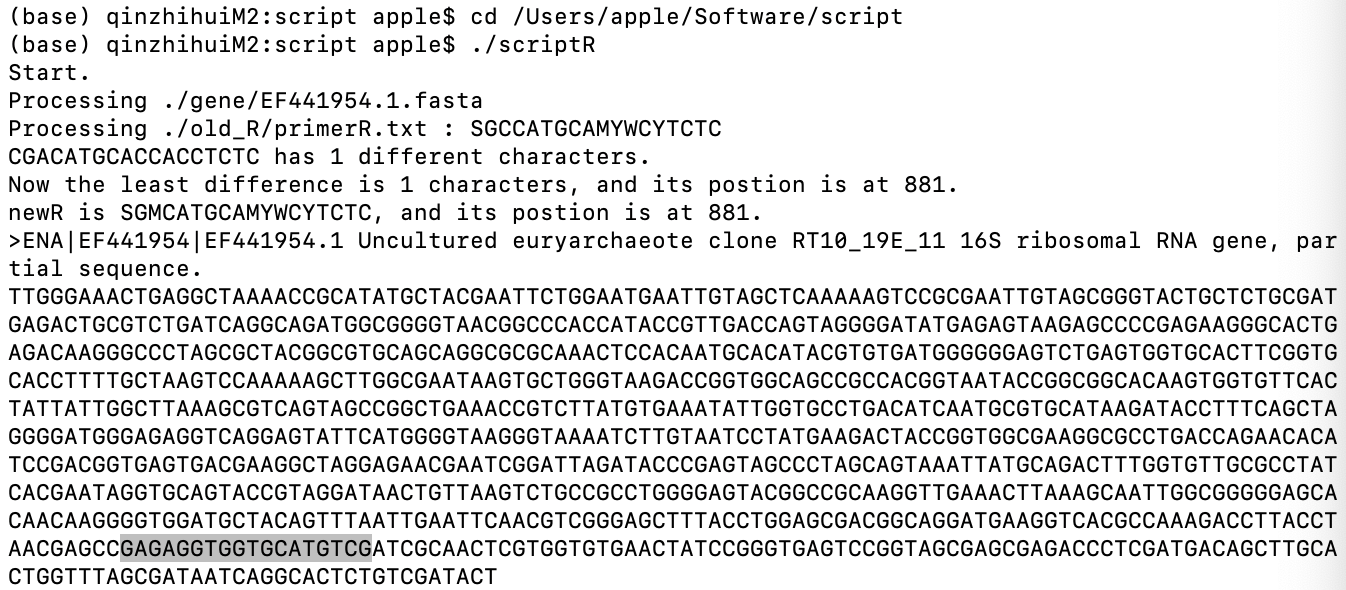

Supplement: Supplementary file 1 [file Data_Sheet_1.zip › supporting/Gene sequences and script execution results/A-784F-1059R and its improved primers/Micrarchaeota/Archaea-784F-1059R-M3-R4.png]

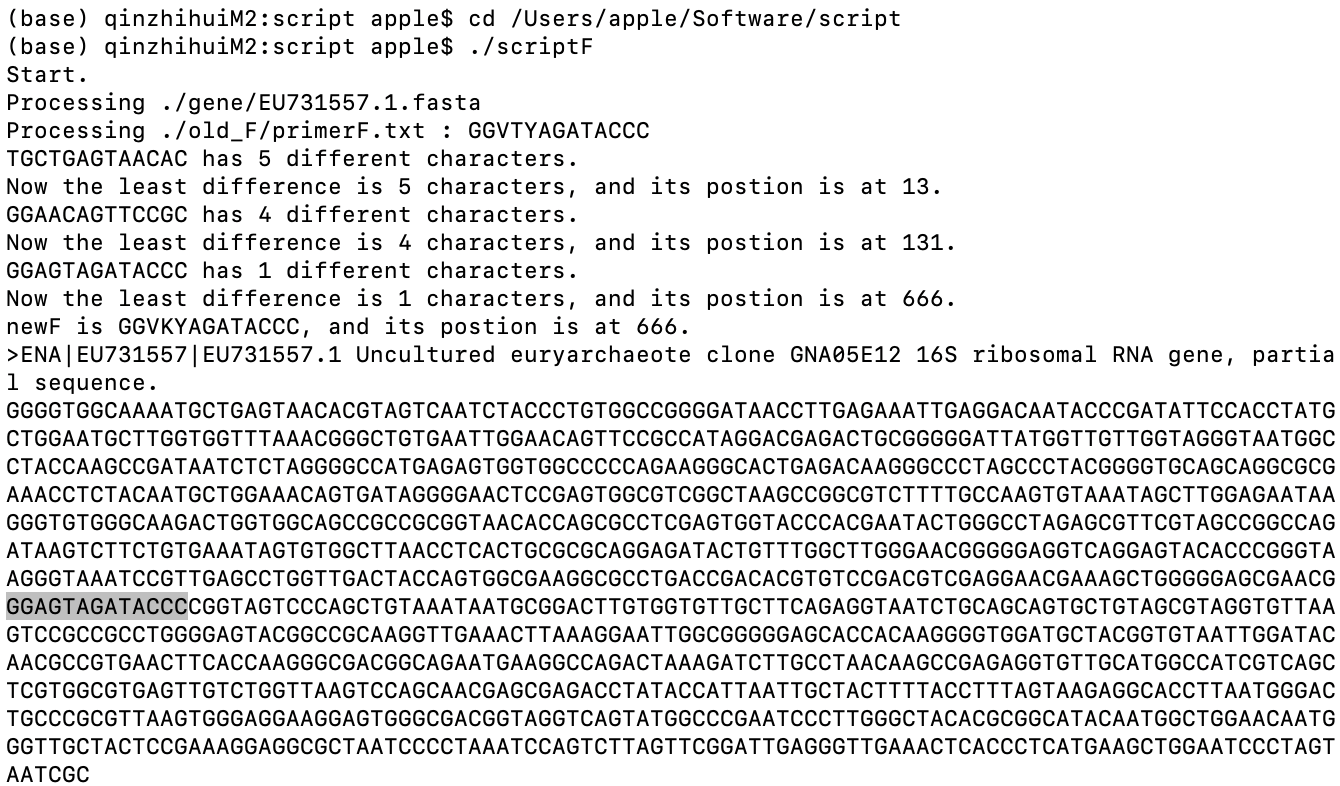

Supplement: Supplementary file 1 [file Data_Sheet_1.zip › supporting/Gene sequences and script execution results/A-784F-1059R and its improved primers/Micrarchaeota/Archaea-784F-1059R-M3-F3.png]

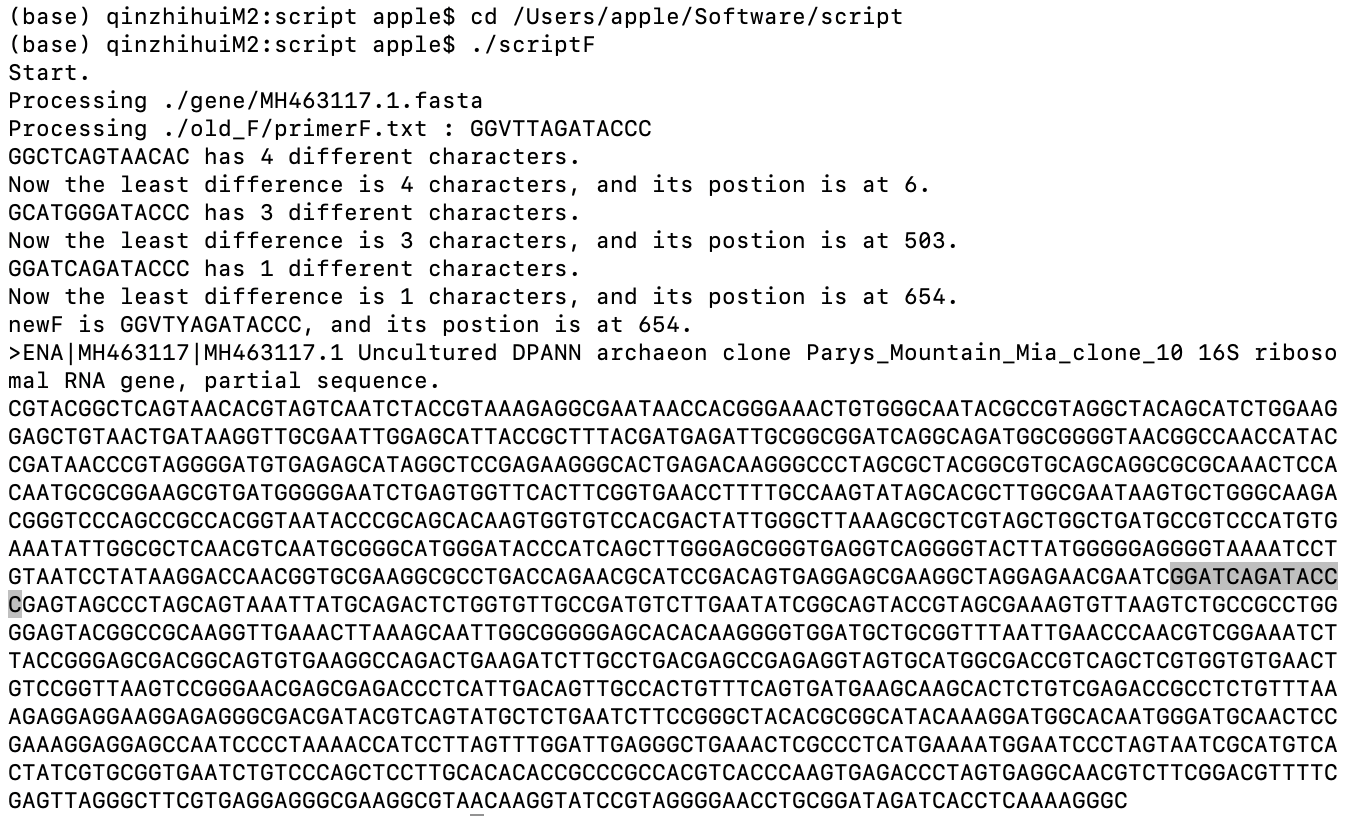

Supplement: Supplementary file 1 [file Data_Sheet_1.zip › supporting/Gene sequences and script execution results/A-784F-1059R and its improved primers/Micrarchaeota/Archaea-784F-1059R-M3-F2.png]

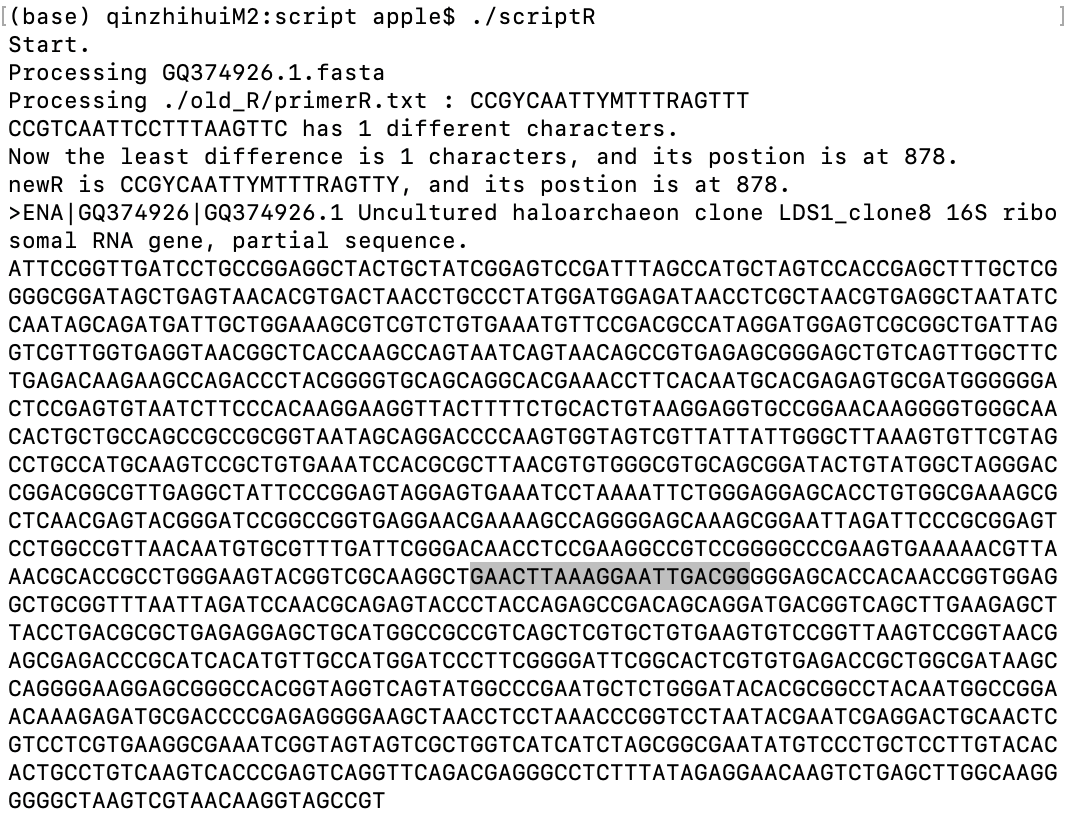

Supplement: Supplementary file 1 [file Data_Sheet_1.zip › supporting/Gene sequences and script execution results/BAE-515F-926R and its improved primers/Nanohaloarchaeota/BAE-515F-1492R-M5-R.png]

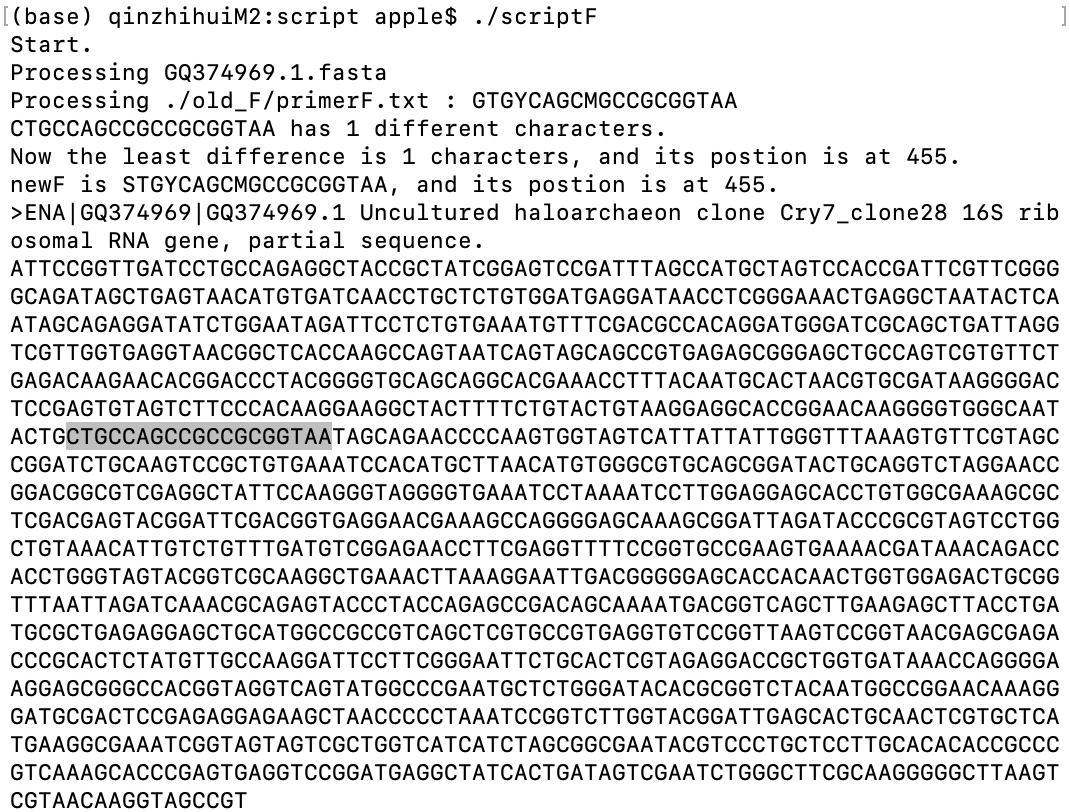

Supplement: Supplementary file 1 [file Data_Sheet_1.zip › supporting/Gene sequences and script execution results/BAE-515F-926R and its improved primers/Nanohaloarchaeota/BAE-515F-1492R-M5-F.png]

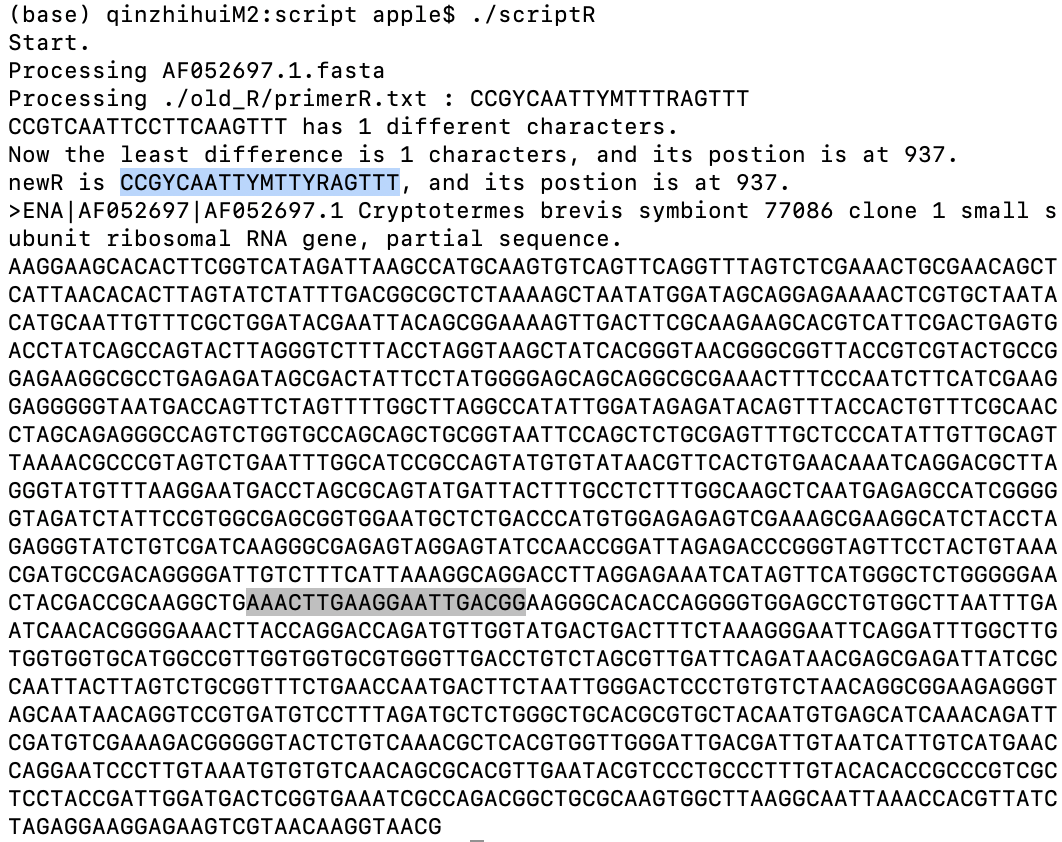

Supplement: Supplementary file 1 [file Data_Sheet_1.zip › supporting/Gene sequences and script execution results/BAE-515F-926R and its improved primers/Excavata/BAE-M1-R.png]

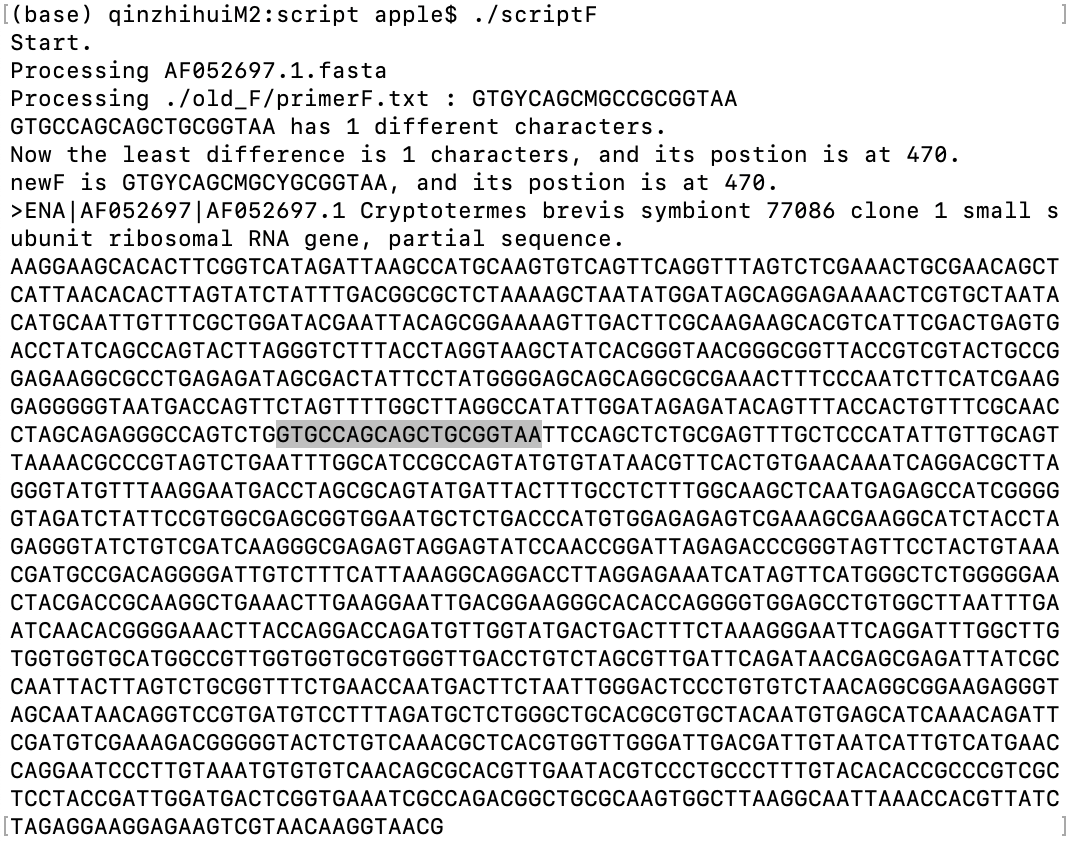

Supplement: Supplementary file 1 [file Data_Sheet_1.zip › supporting/Gene sequences and script execution results/BAE-515F-926R and its improved primers/Excavata/BAE-M1-F.png]

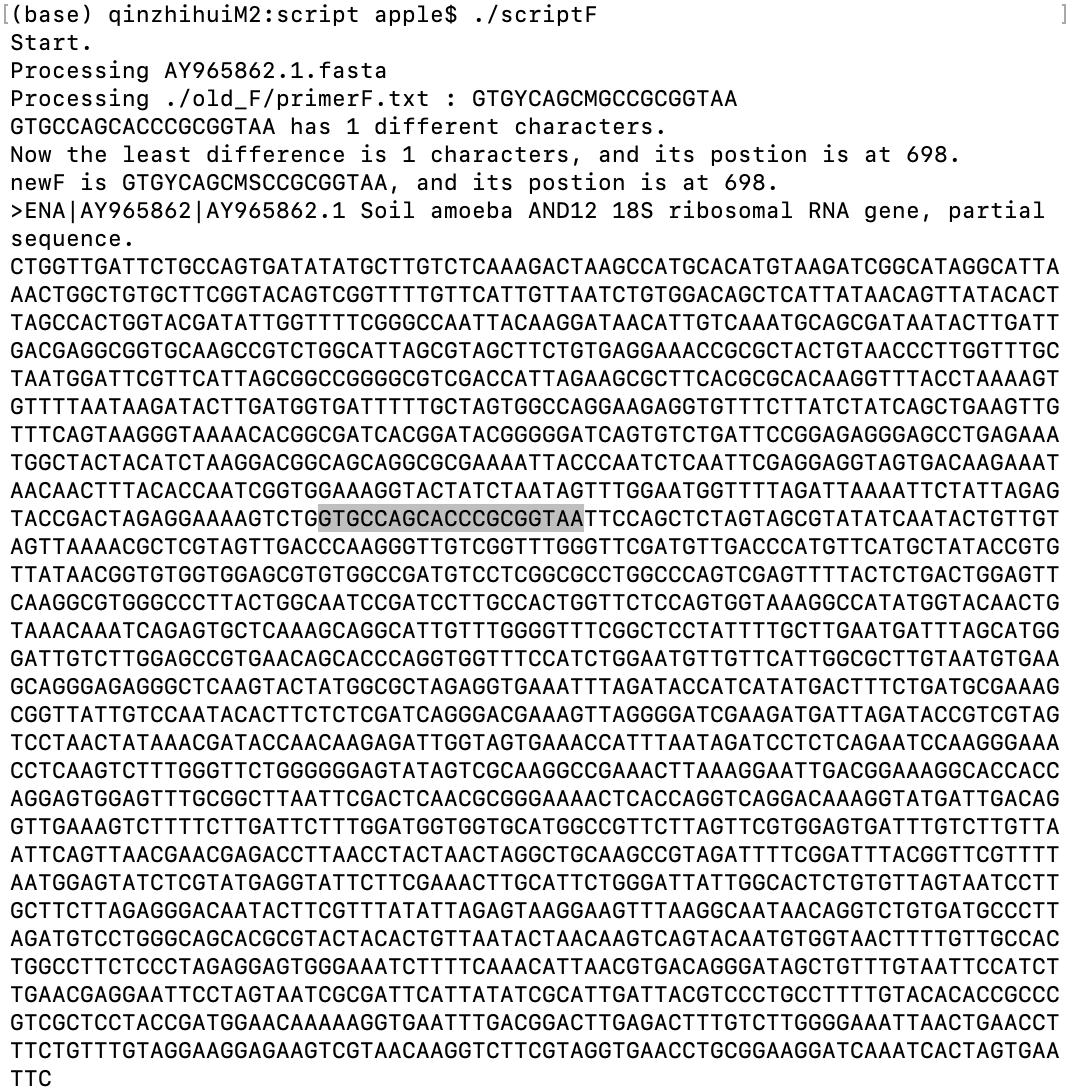

Supplement: Supplementary file 1 [file Data_Sheet_1.zip › supporting/Gene sequences and script execution results/BAE-515F-926R and its improved primers/Discoba/BAE-515F-926R-M2-F1.png]

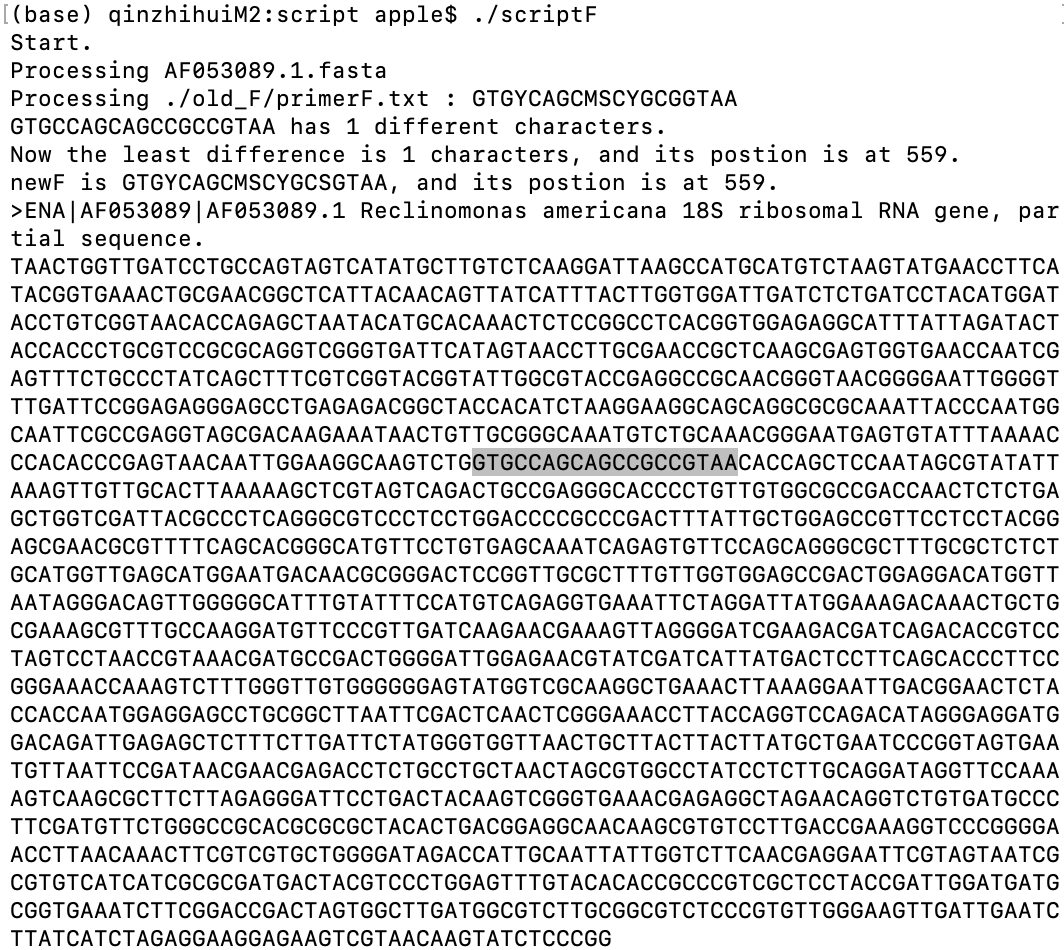

Supplement: Supplementary file 1 [file Data_Sheet_1.zip › supporting/Gene sequences and script execution results/BAE-515F-926R and its improved primers/Discoba/BAE-515F-926R-M2-F3.png]

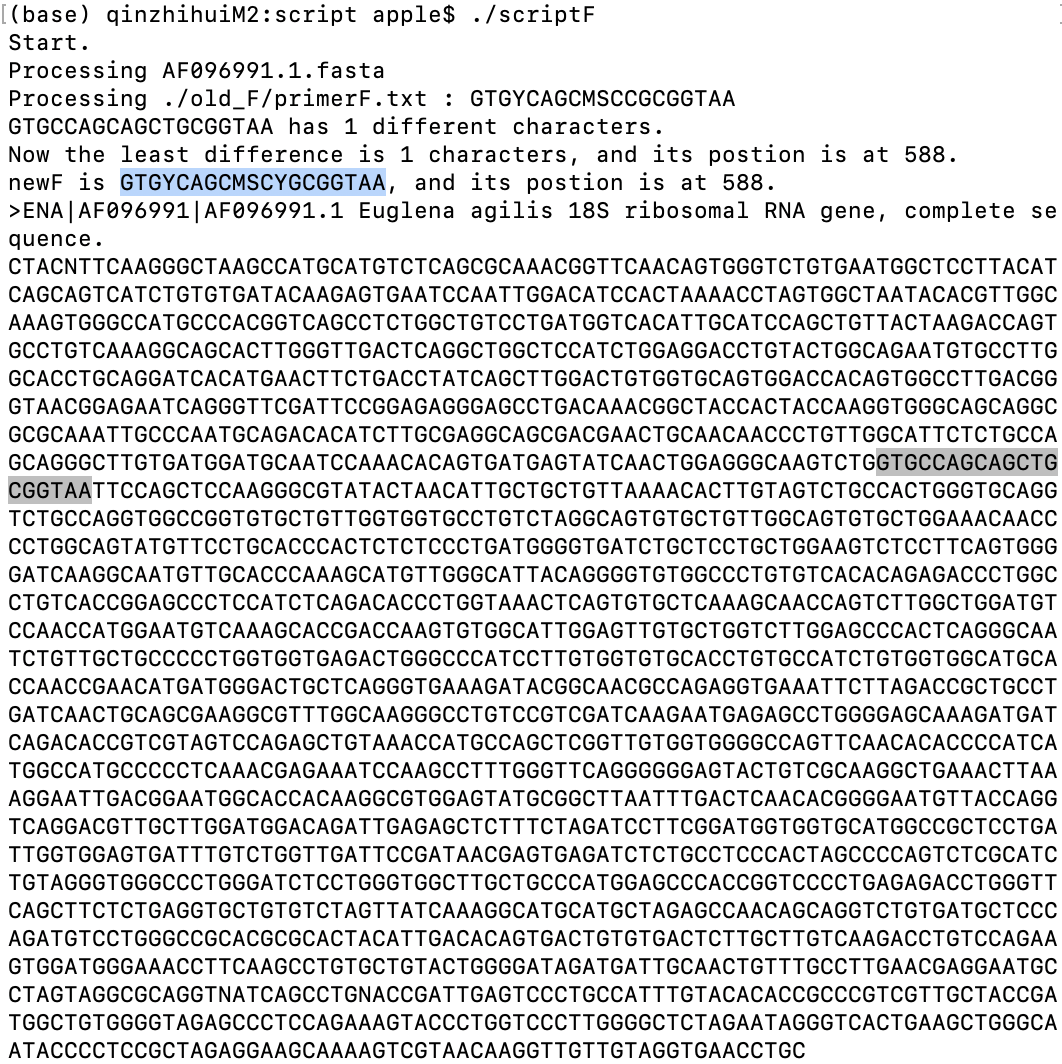

Supplement: Supplementary file 1 [file Data_Sheet_1.zip › supporting/Gene sequences and script execution results/BAE-515F-926R and its improved primers/Discoba/BAE-515F-926R-M2-F2.png]

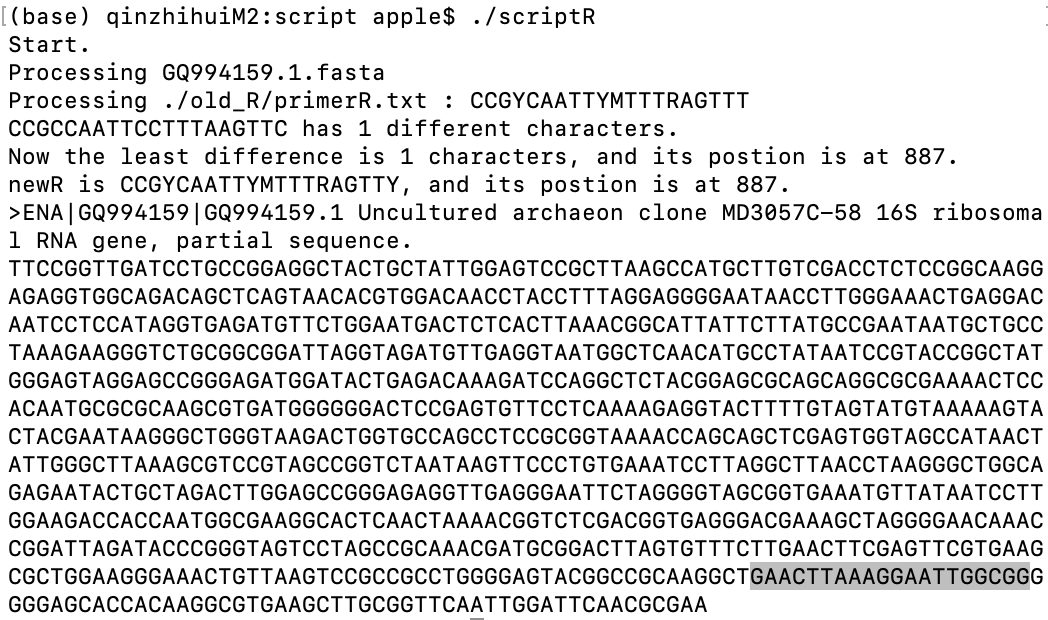

Supplement: Supplementary file 1 [file Data_Sheet_1.zip › supporting/Gene sequences and script execution results/BAE-515F-926R and its improved primers/Altiarchaeota/BAE-515F-926R-M6-R.png]

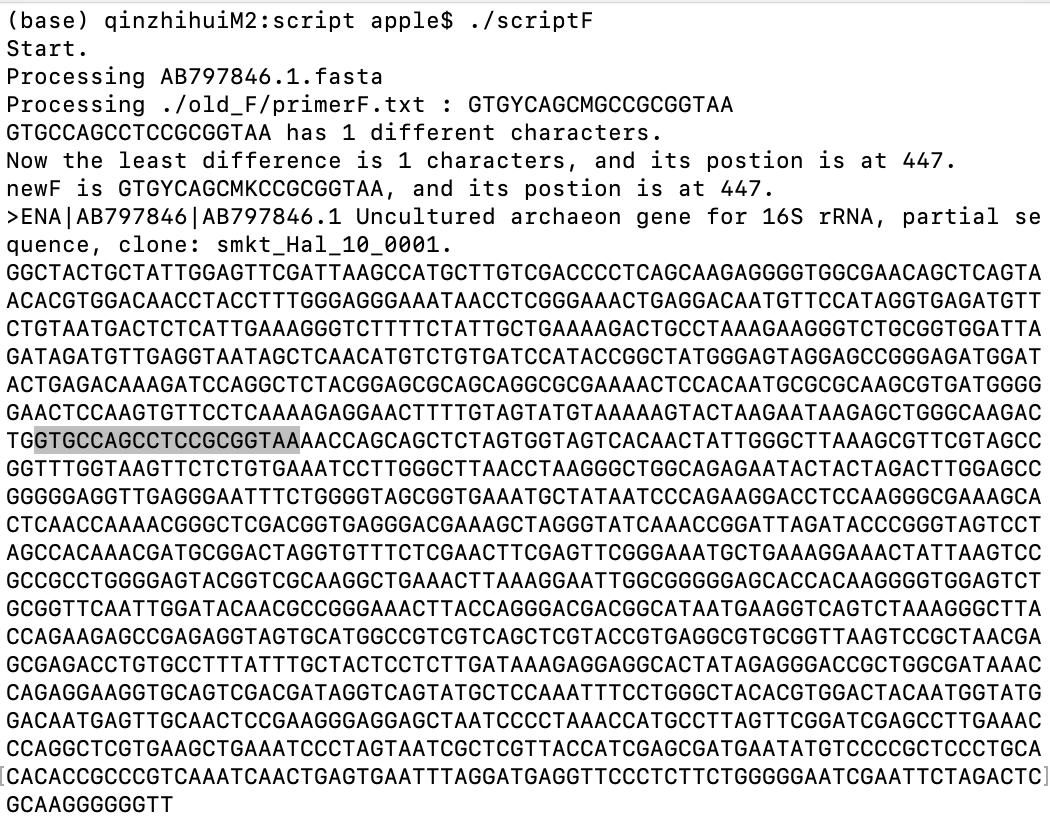

Supplement: Supplementary file 1 [file Data_Sheet_1.zip › supporting/Gene sequences and script execution results/BAE-515F-926R and its improved primers/Altiarchaeota/BAE-515F-926R-M6-F.png]

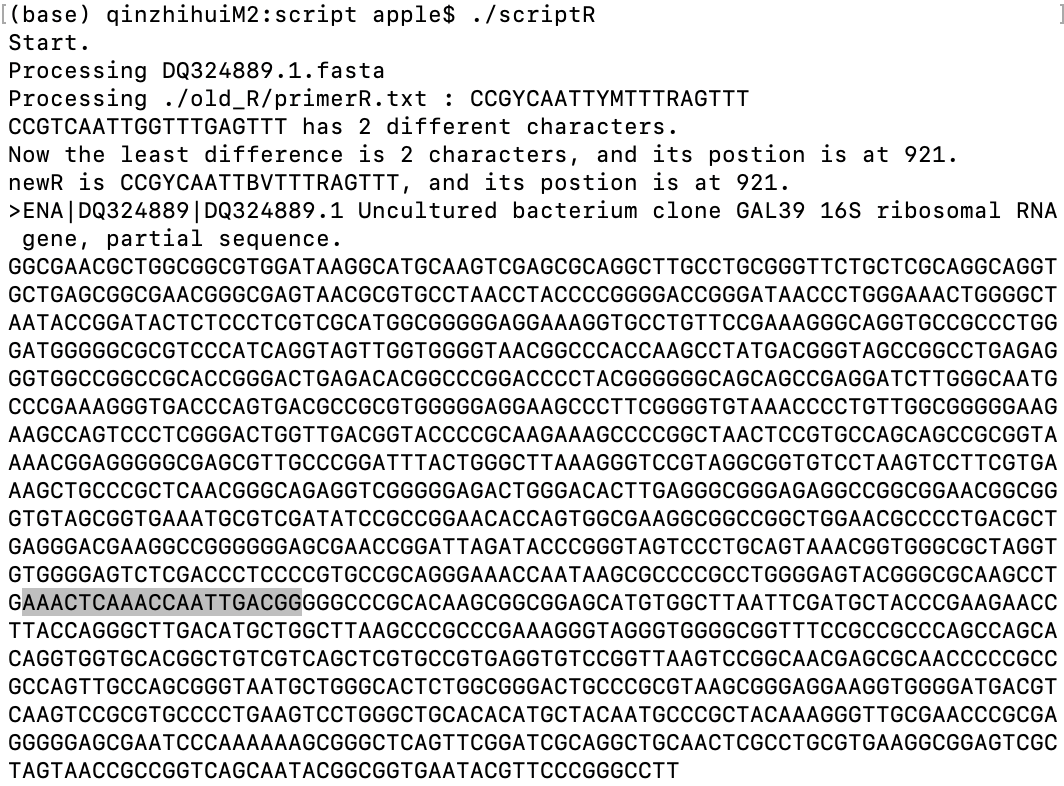

Supplement: Supplementary file 1 [file Data_Sheet_1.zip › supporting/Gene sequences and script execution results/BAE-515F-926R and its improved primers/Fervidibacteria/BAE-515F-926R-M3-F.png]

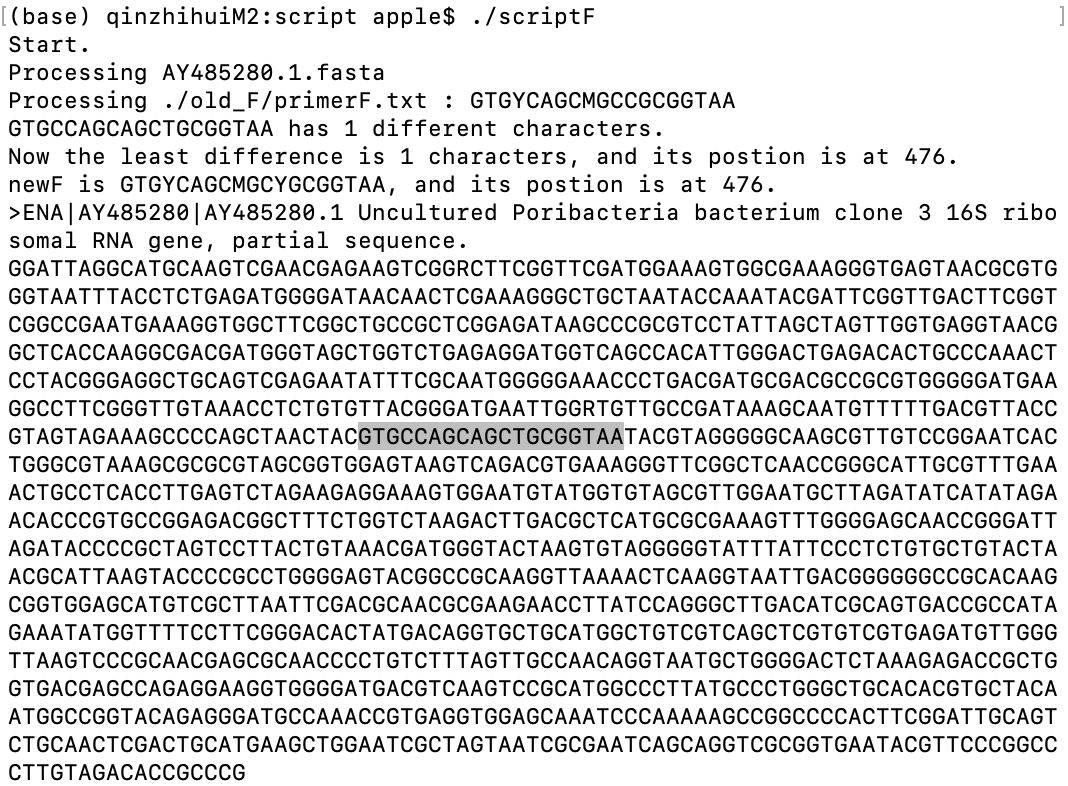

Supplement: Supplementary file 1 [file Data_Sheet_1.zip › supporting/Gene sequences and script execution results/BAE-515F-926R and its improved primers/Poribacteria/BAE-515F-926R-M4-F.png]

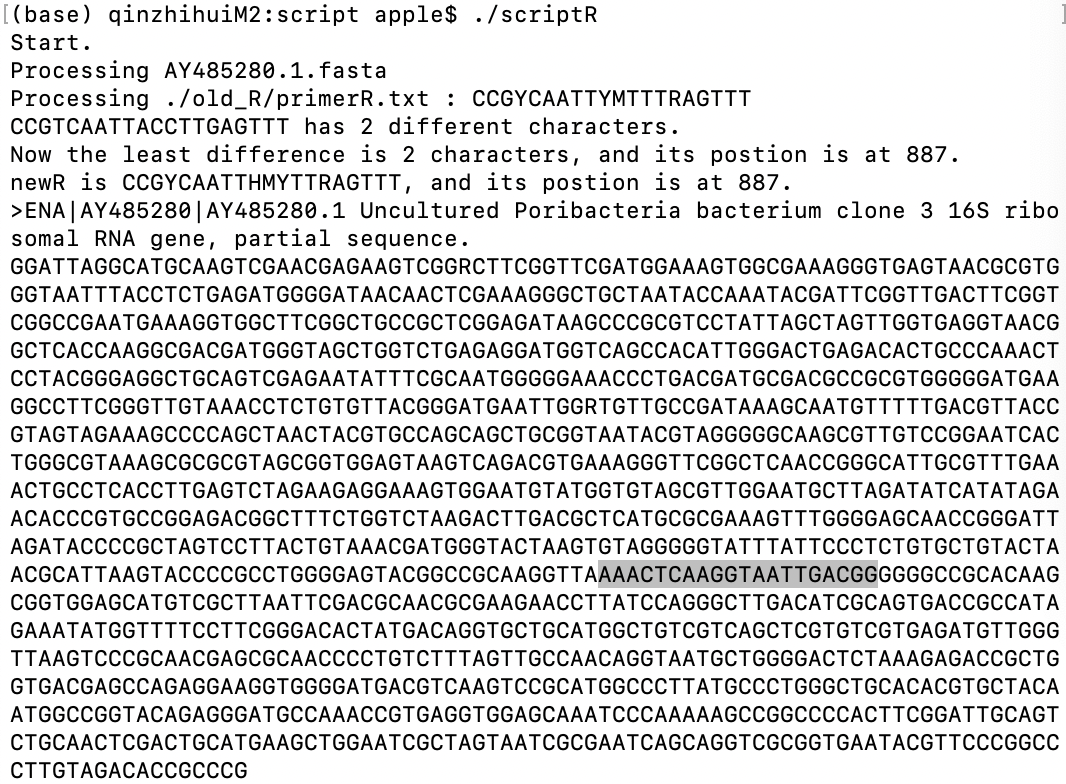

Supplement: Supplementary file 1 [file Data_Sheet_1.zip › supporting/Gene sequences and script execution results/BAE-515F-926R and its improved primers/Poribacteria/BAE-515F-926R-M4-R.png]

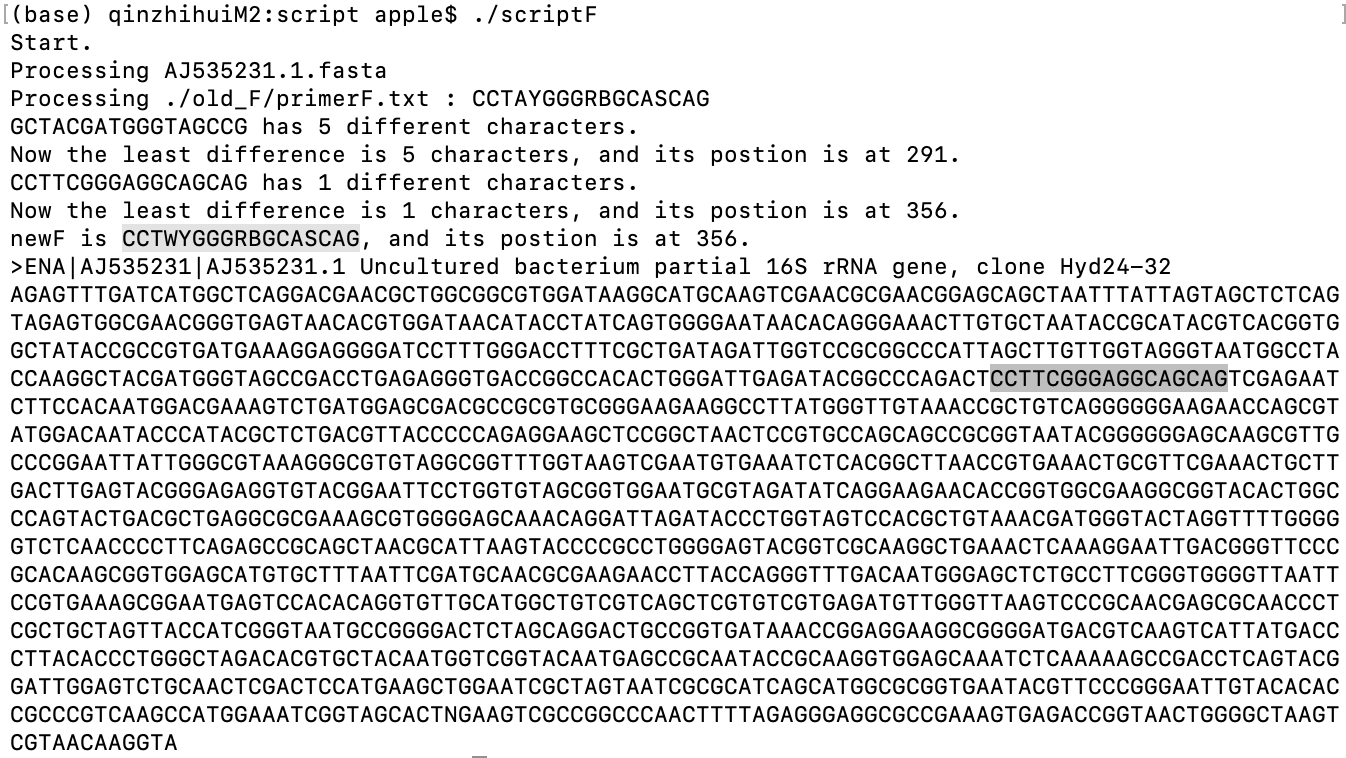

Supplement: Supplementary file 1 [file Data_Sheet_1.zip › supporting/Gene sequences and script execution results/BA-341F-806R and its improved primers/Fermentibacterota/BA-341F-806R-M2-F.png]

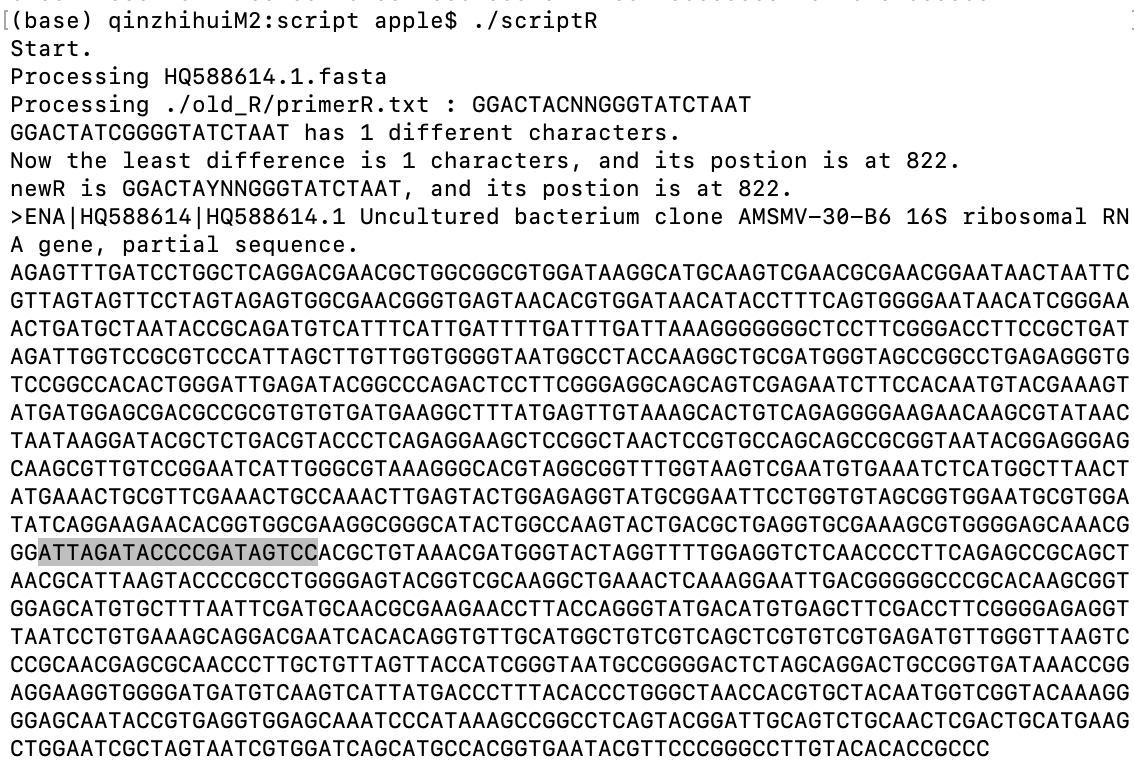

Supplement: Supplementary file 1 [file Data_Sheet_1.zip › supporting/Gene sequences and script execution results/BA-341F-806R and its improved primers/Fermentibacterota/BA-341F-806R-M2-R.png]

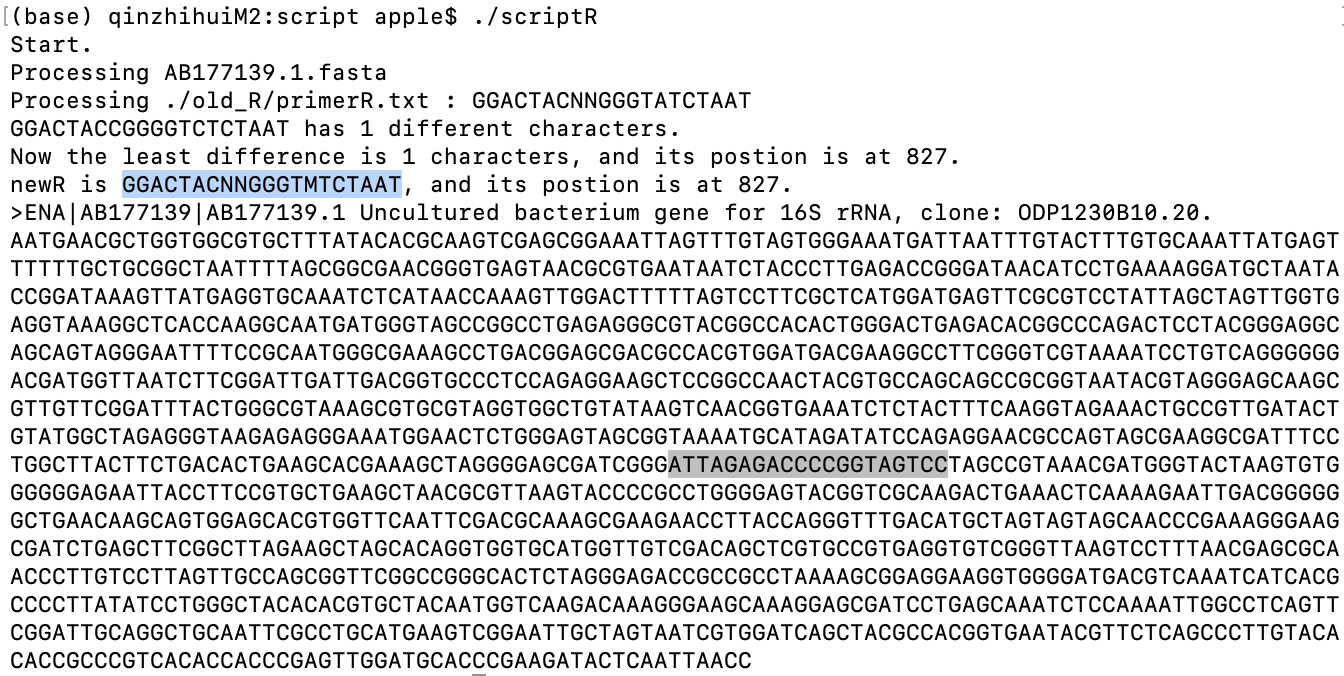

Supplement: Supplementary file 1 [file Data_Sheet_1.zip › supporting/Gene sequences and script execution results/BA-341F-806R and its improved primers/Aerophobota/BA-341F-806R-M1-R.png]

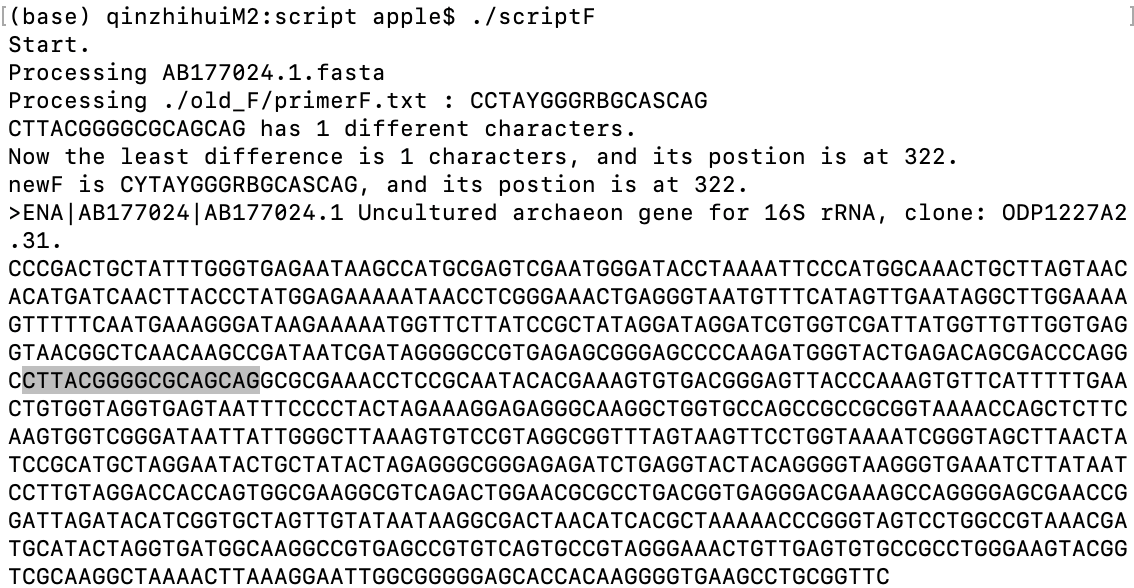

Supplement: Supplementary file 1 [file Data_Sheet_1.zip › supporting/Gene sequences and script execution results/BA-341F-806R and its improved primers/Asgardarchaeota/BArchaea-341F-806R-M3-F1.png]

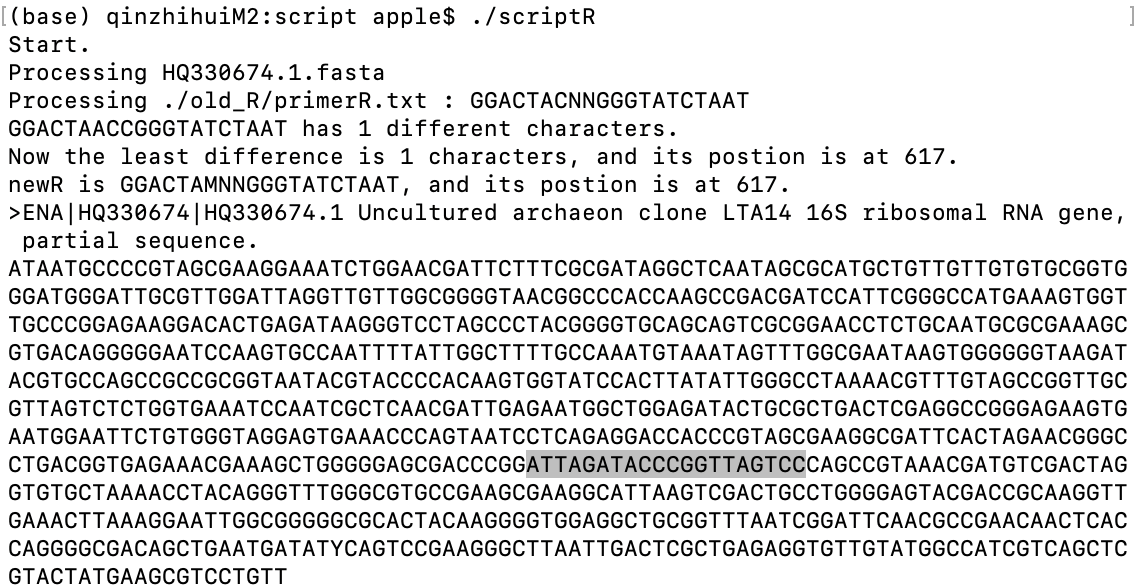

Supplement: Supplementary file 1 [file Data_Sheet_1.zip › supporting/Gene sequences and script execution results/BA-341F-806R and its improved primers/Iainarchaeota/BA-341F-806R-M4-R1.png]

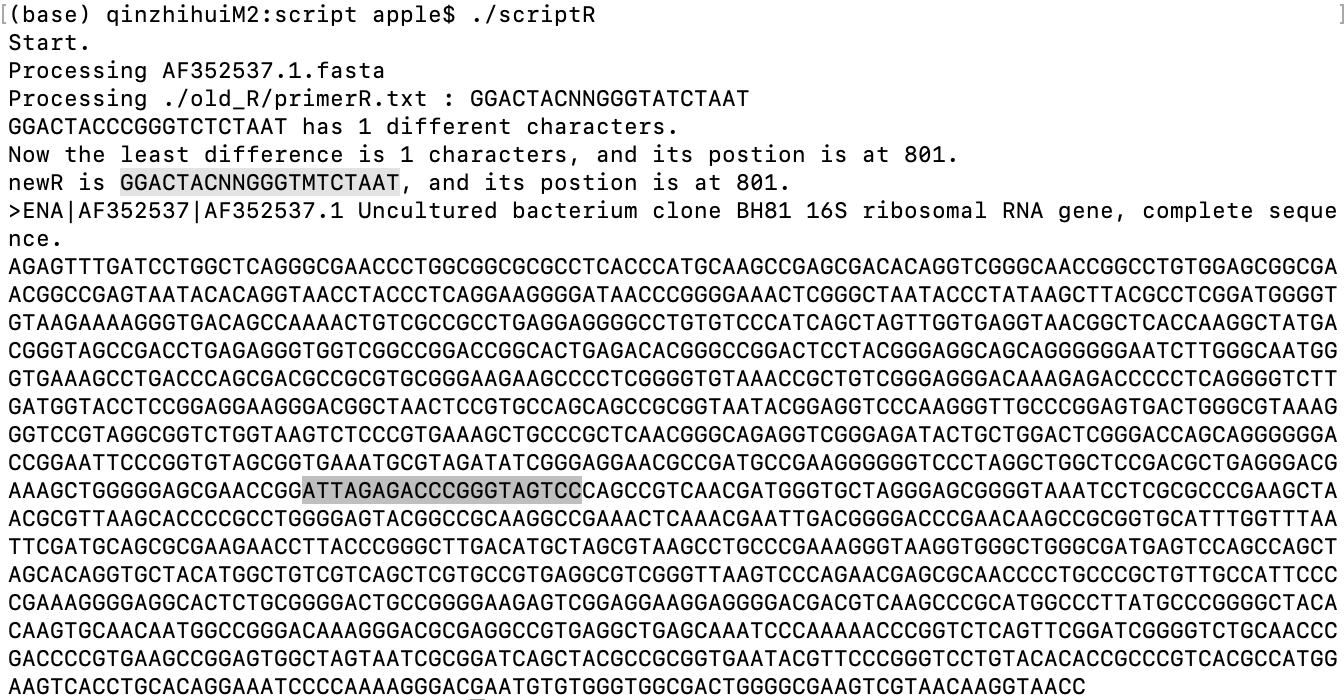

Supplement: Supplementary file 1 [file Data_Sheet_1.zip › supporting/Gene sequences and script execution results/BA-341F-806R and its improved primers/Calescamantes/BA-341F-806R-M1-R.png]

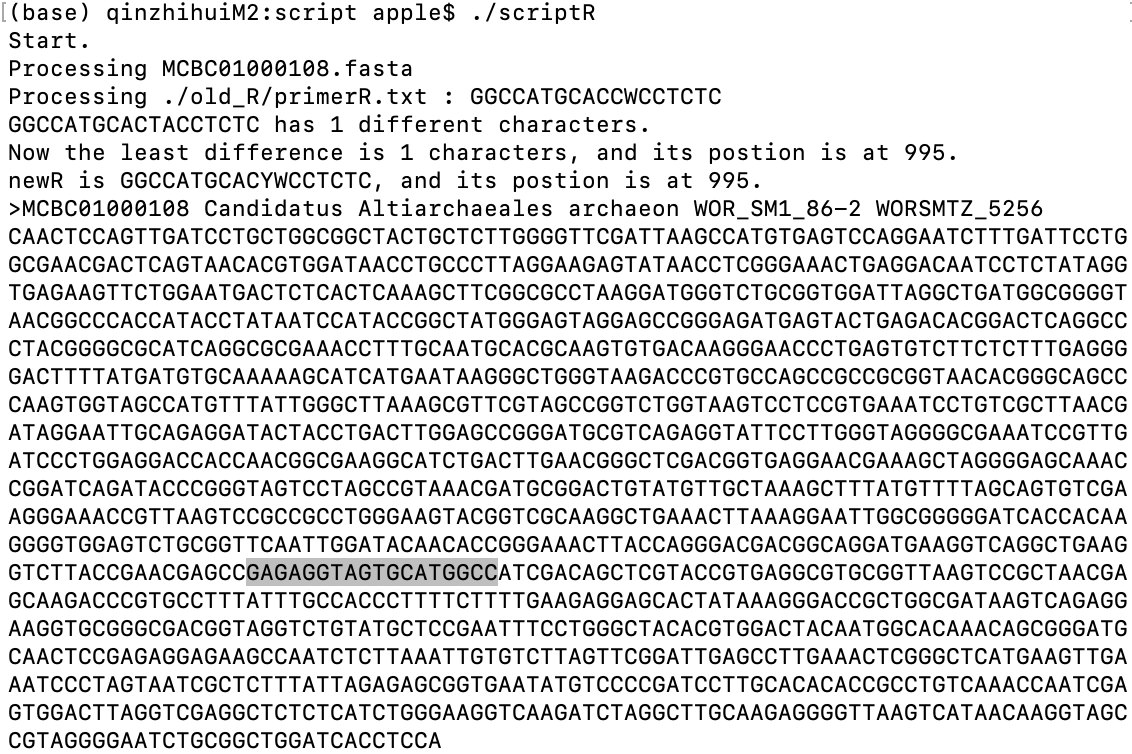

Supplement: Supplementary file 1 [file Data_Sheet_1.zip › supporting/Gene sequences and script execution results/A-341F-1049R and its improved primers/Altiarchaeota/Archaea-341F-1049R-M4-R1.png]

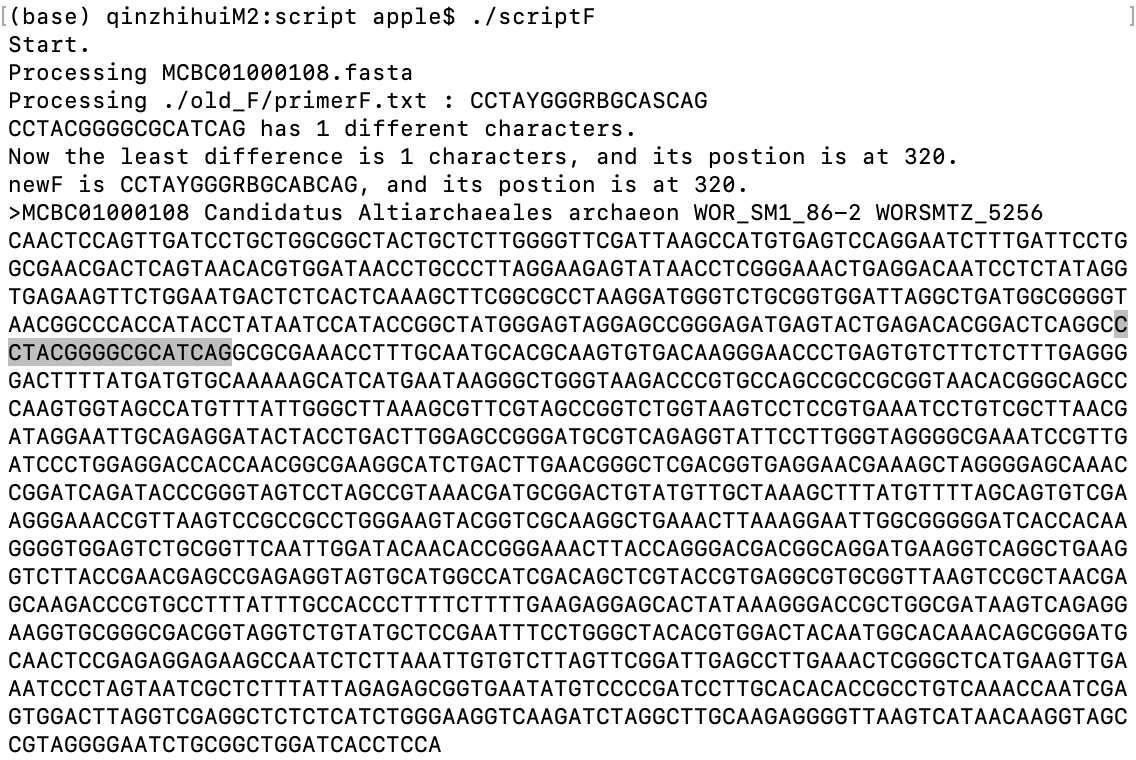

Supplement: Supplementary file 1 [file Data_Sheet_1.zip › supporting/Gene sequences and script execution results/A-341F-1049R and its improved primers/Altiarchaeota/Archaea-341F-1049R-M4-F1.png]

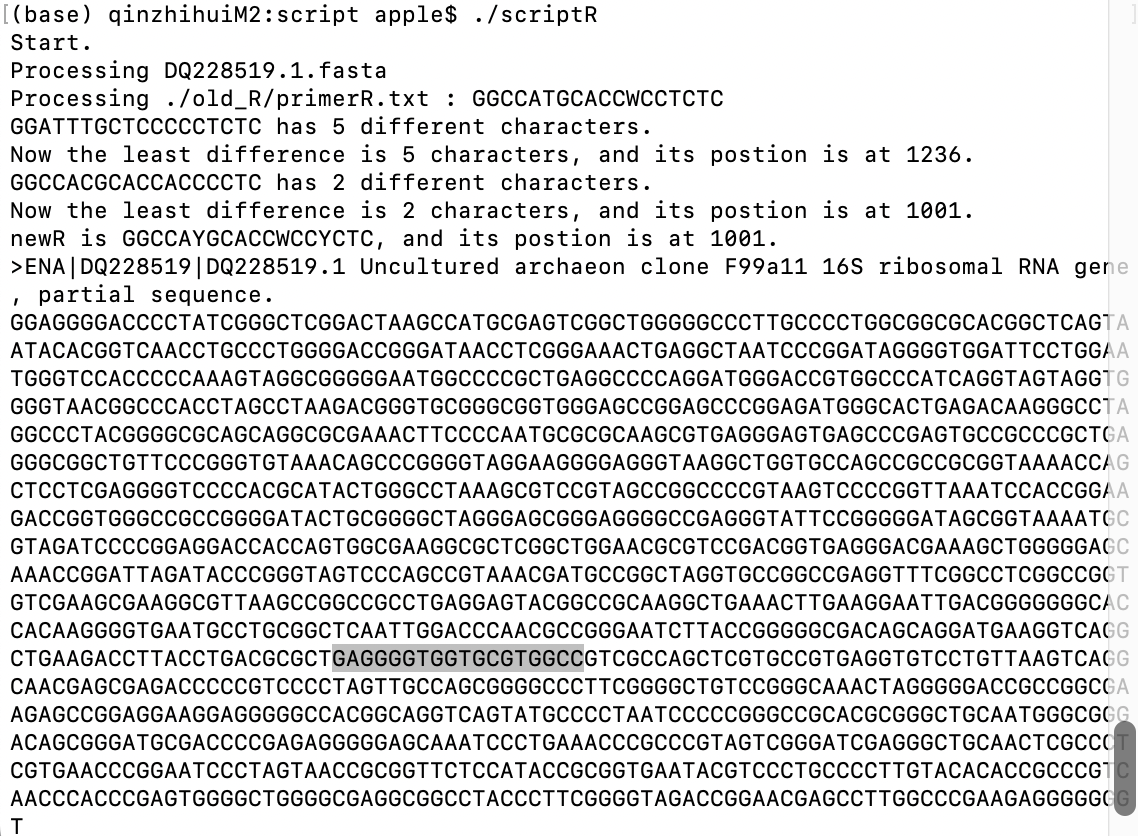

Supplement: Supplementary file 1 [file Data_Sheet_1.zip › supporting/Gene sequences and script execution results/A-341F-1049R and its improved primers/Korarchaeota/Archaea-341F-1059R-M2-R.png]

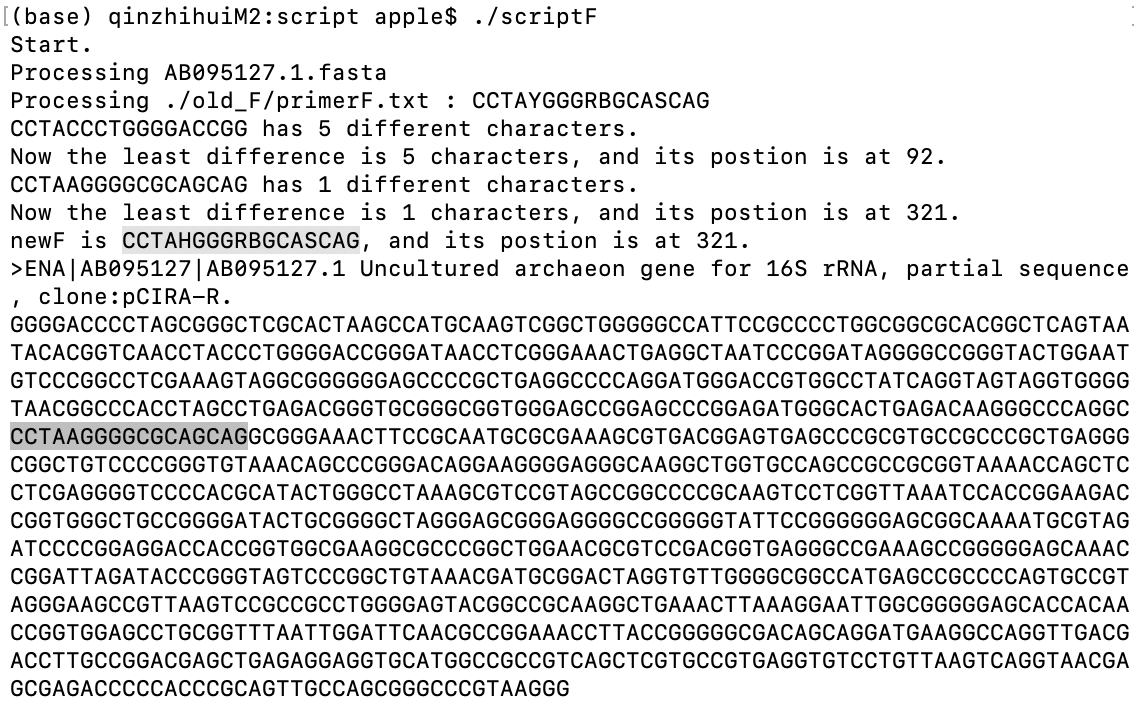

Supplement: Supplementary file 1 [file Data_Sheet_1.zip › supporting/Gene sequences and script execution results/A-341F-1049R and its improved primers/Korarchaeota/Archaea-341F-1059R-M2-F.png]

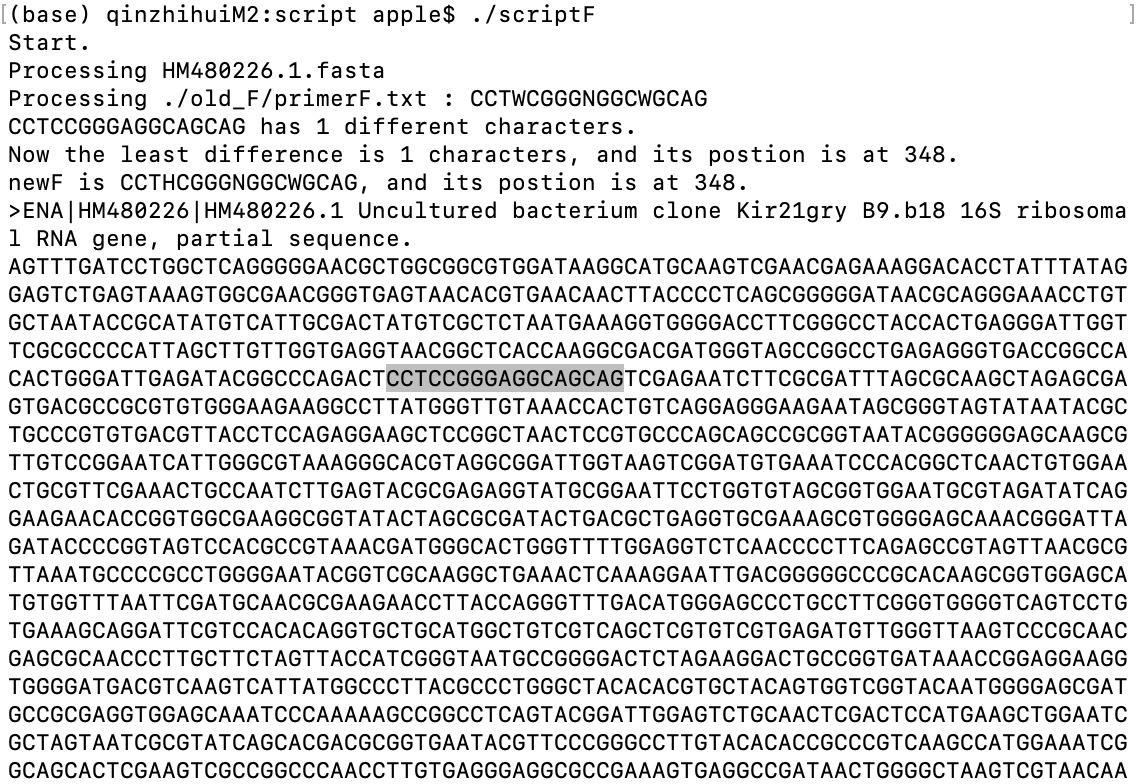

Supplement: Supplementary file 1 [file Data_Sheet_1.zip › supporting/Gene sequences and script execution results/B-341F-806R and its improved primers/Fermentibacterota/B-341F-806R-M3-F2.png]

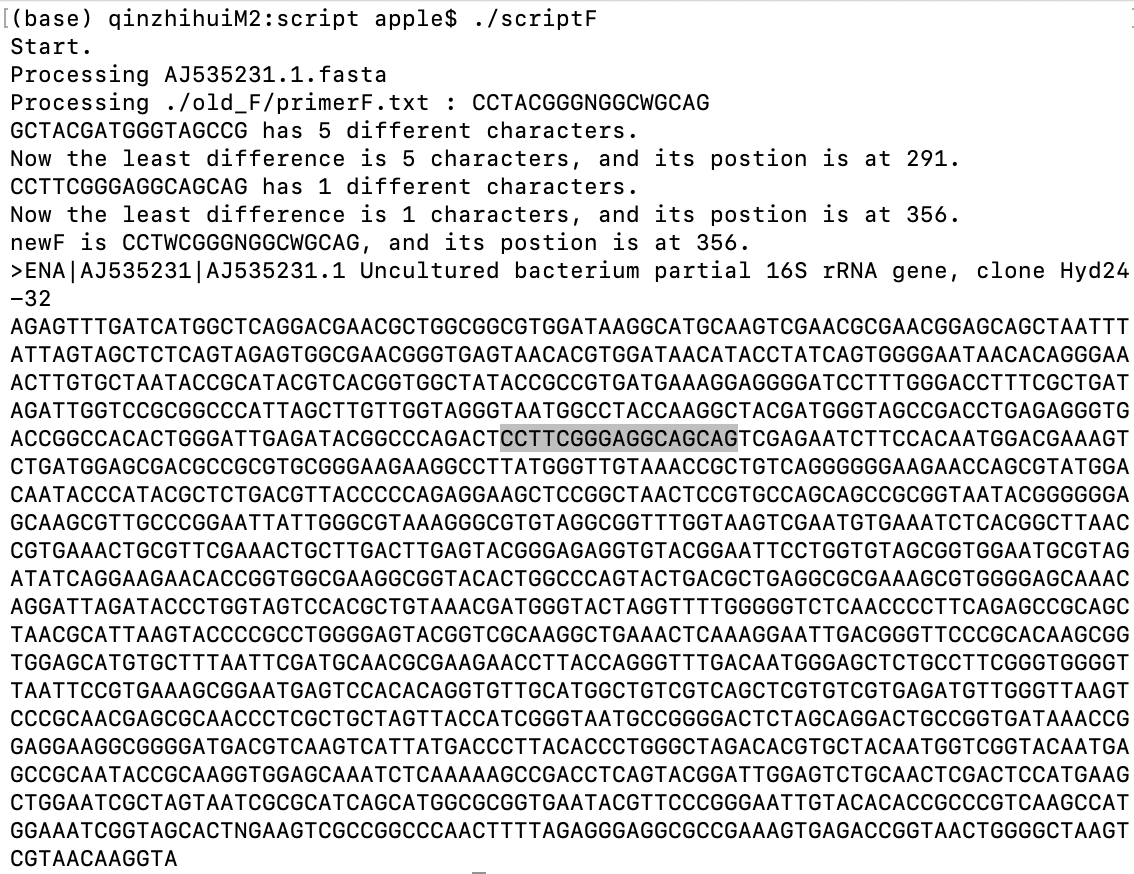

Supplement: Supplementary file 1 [file Data_Sheet_1.zip › supporting/Gene sequences and script execution results/B-341F-806R and its improved primers/Fermentibacterota/B-341F-806R-M3-F1.png]

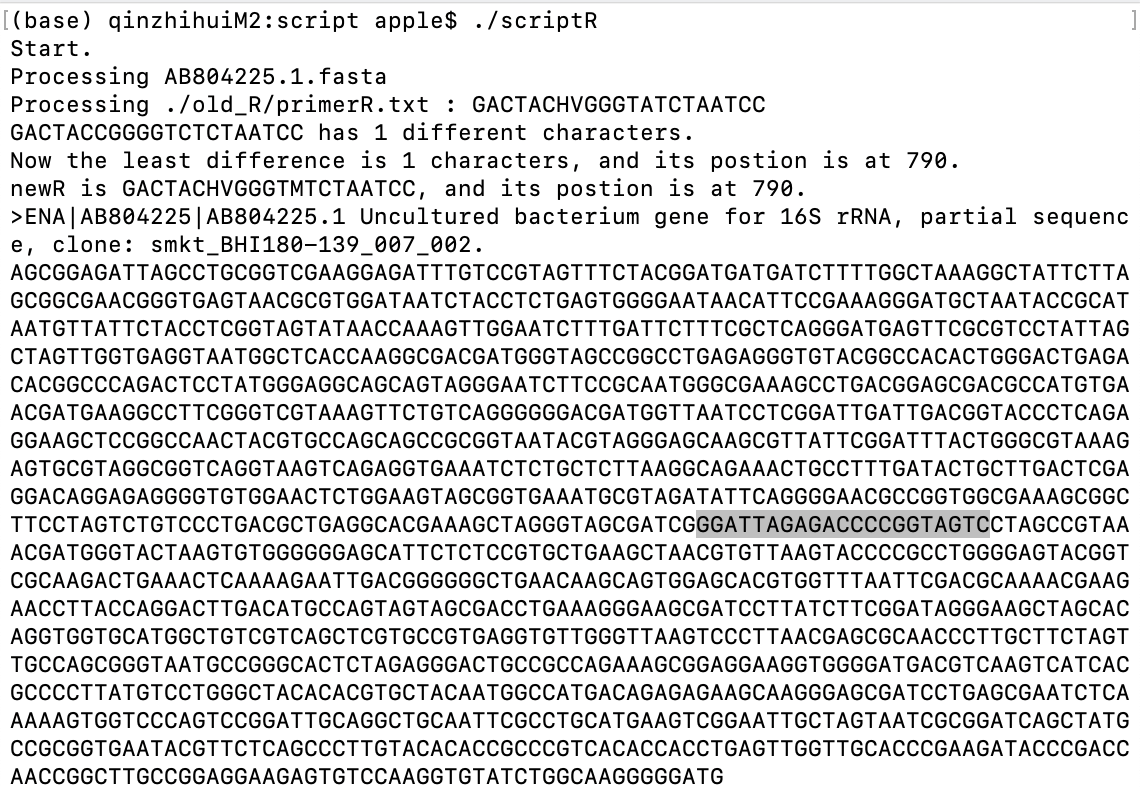

Supplement: Supplementary file 1 [file Data_Sheet_1.zip › supporting/Gene sequences and script execution results/B-341F-806R and its improved primers/Aerophobota/B-341F-806R-M1-R.png]

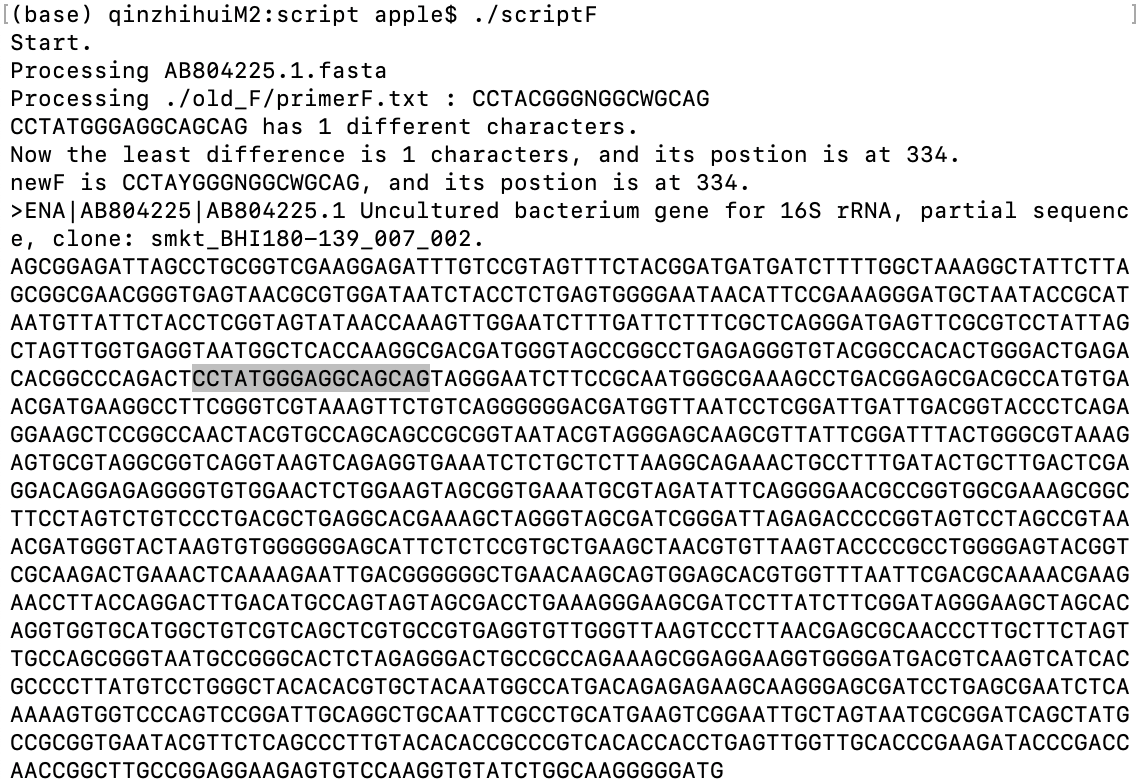

Supplement: Supplementary file 1 [file Data_Sheet_1.zip › supporting/Gene sequences and script execution results/B-341F-806R and its improved primers/Aerophobota/B-341F-806R-M1-F.png]

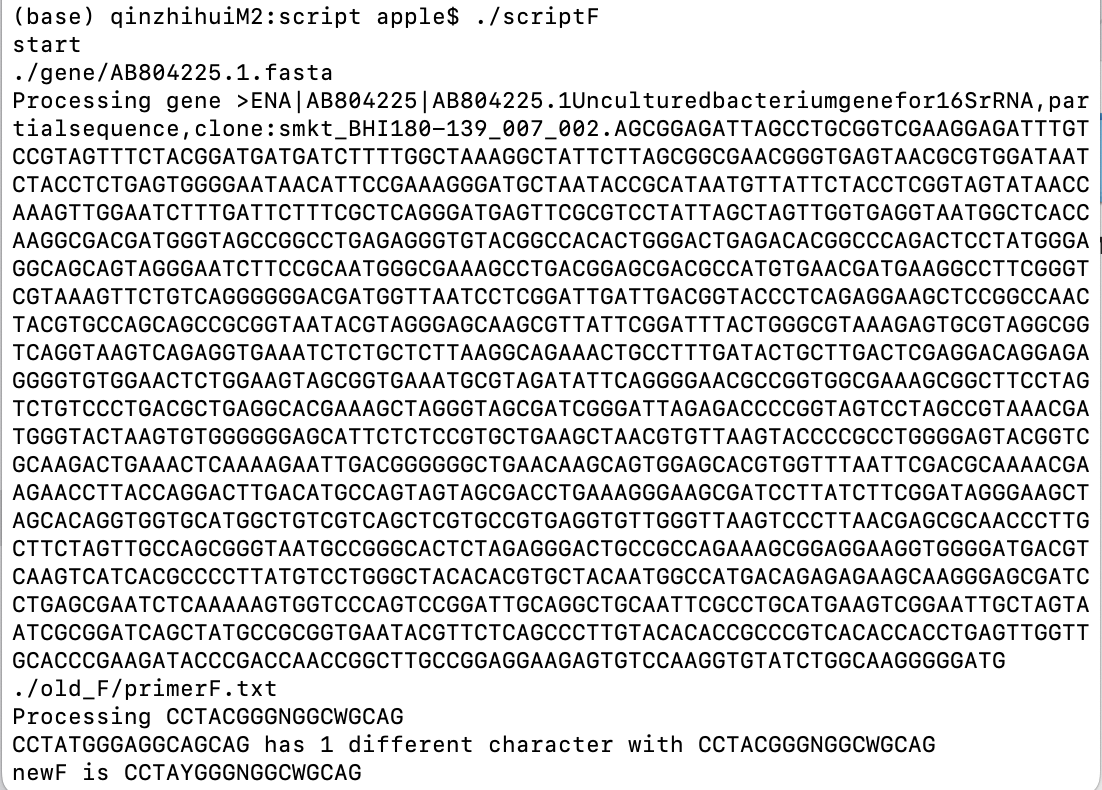

Supplement: Supplementary file 1 [file Data_Sheet_1.zip › supporting/Gene sequences and script execution results/B-341F-806R and its improved primers/Aerophobota/341F-785R-M2F.png]

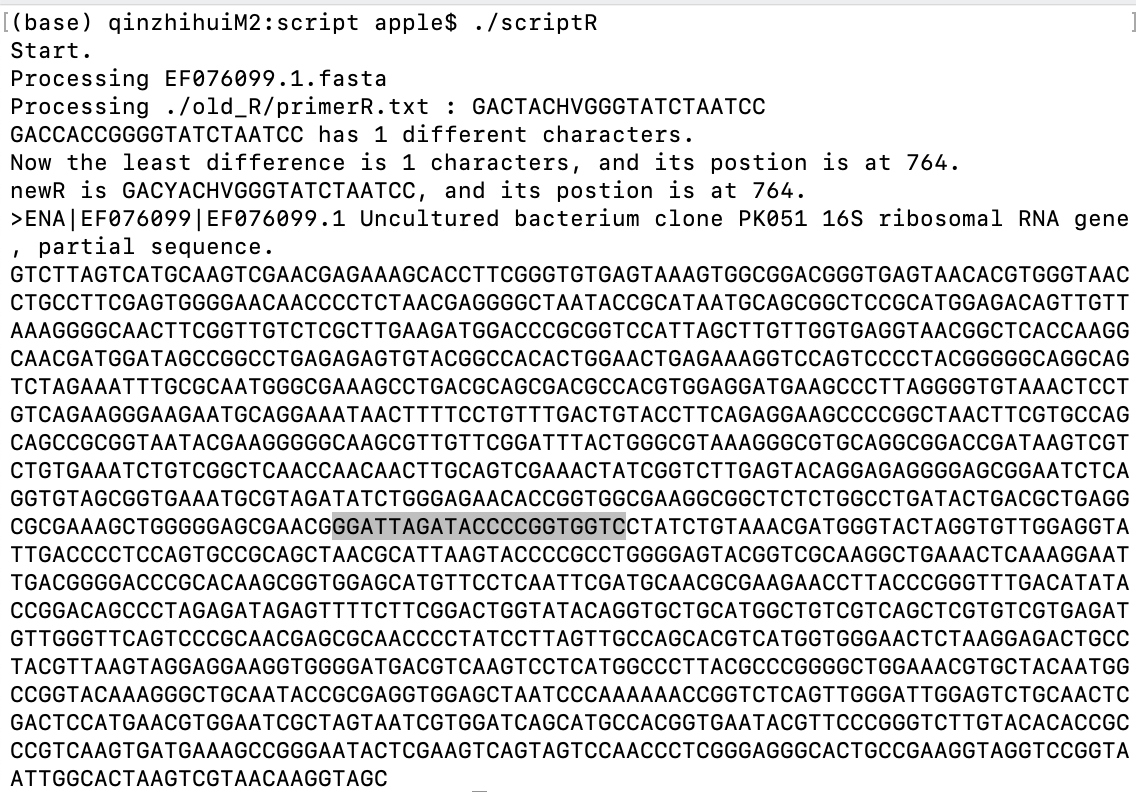

Supplement: Supplementary file 1 [file Data_Sheet_1.zip › supporting/Gene sequences and script execution results/B-341F-806R and its improved primers/PAUC34f/B-341F-806R-M2-R.png]

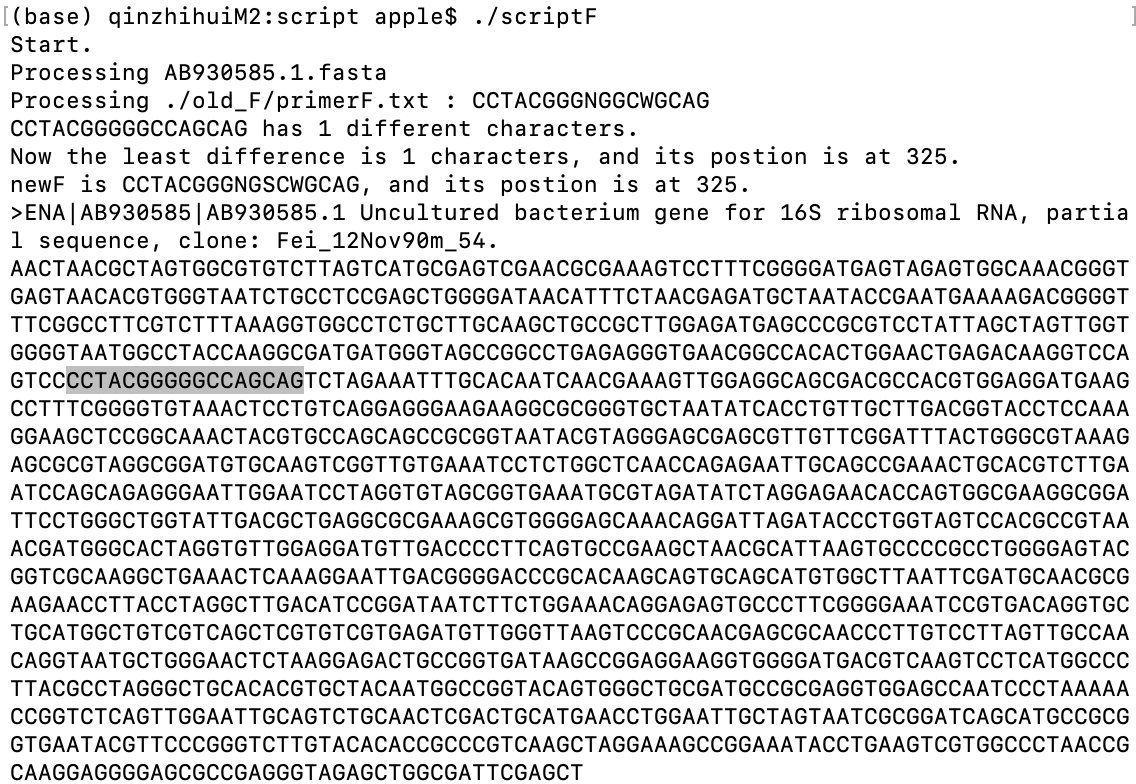

Supplement: Supplementary file 1 [file Data_Sheet_1.zip › supporting/Gene sequences and script execution results/B-341F-806R and its improved primers/PAUC34f/B-341F-806R-M2-F.png]

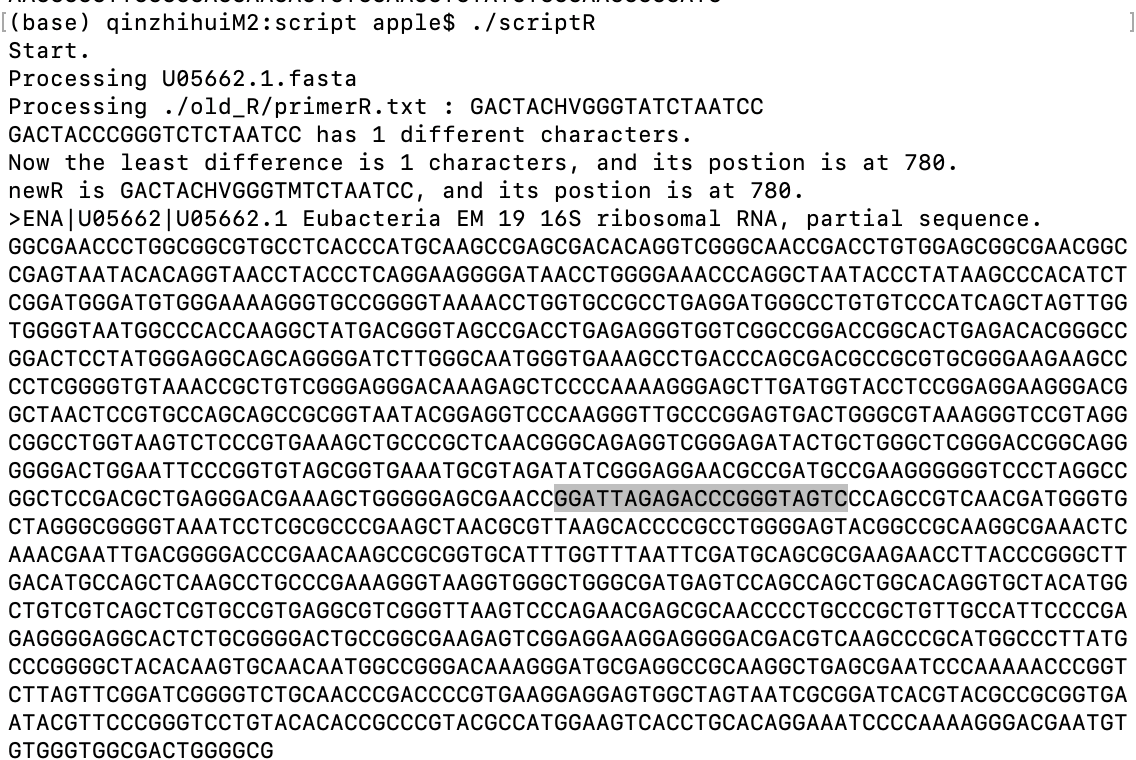

Supplement: Supplementary file 1 [file Data_Sheet_1.zip › supporting/Gene sequences and script execution results/B-341F-806R and its improved primers/Calescamantes/B-341F-806R-M1-R.png]

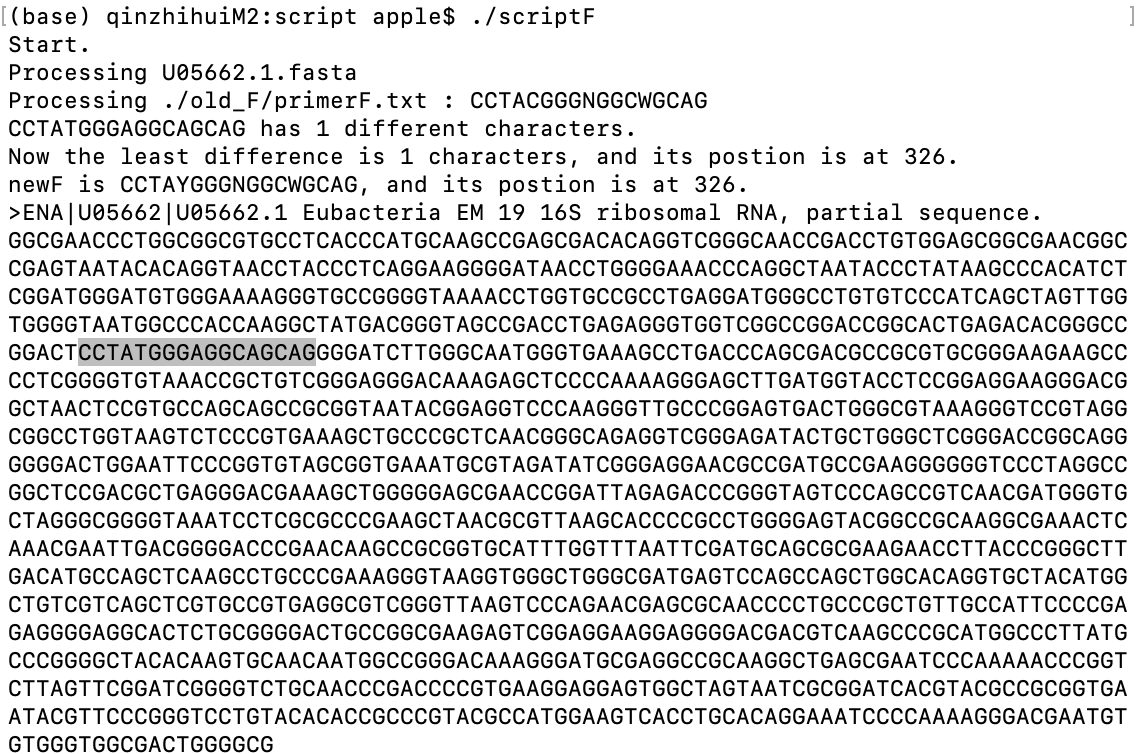

Supplement: Supplementary file 1 [file Data_Sheet_1.zip › supporting/Gene sequences and script execution results/B-341F-806R and its improved primers/Calescamantes/B-341F-806R-M1-F.png]

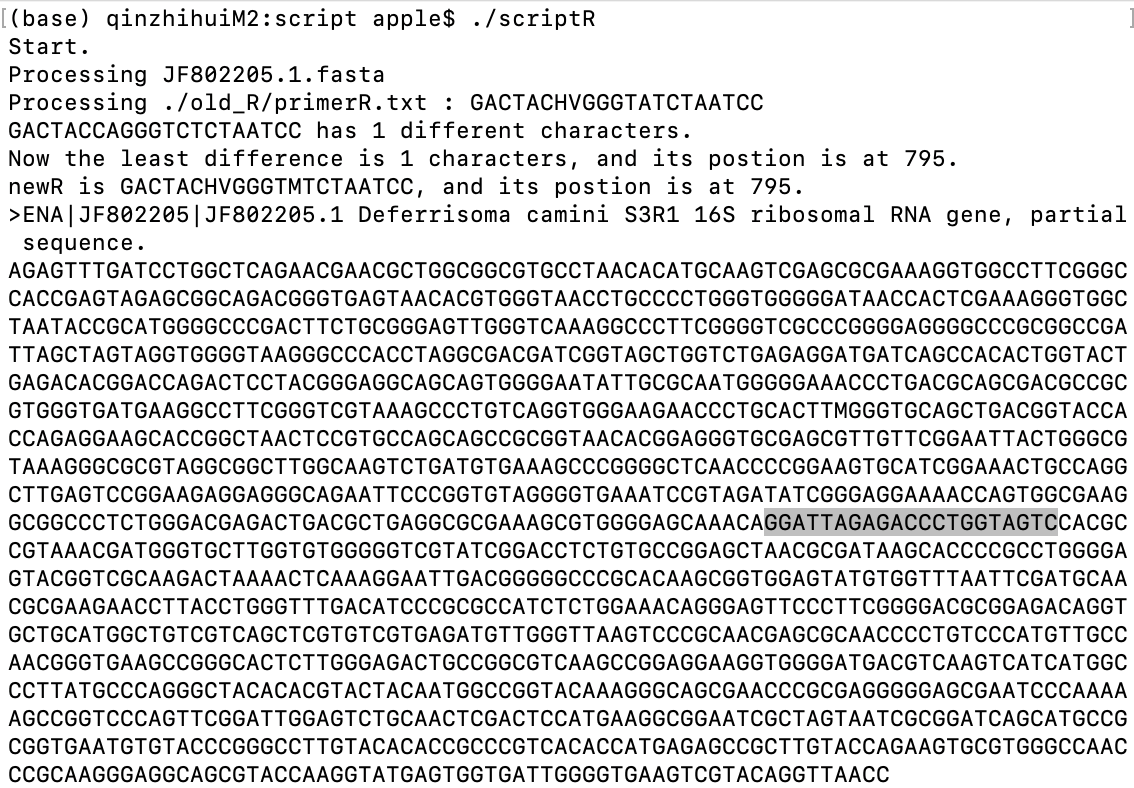

Supplement: Supplementary file 1 [file Data_Sheet_1.zip › supporting/Gene sequences and script execution results/B-341F-806R and its improved primers/Deferrisomatota/B-341F-806R-M1-R.png]

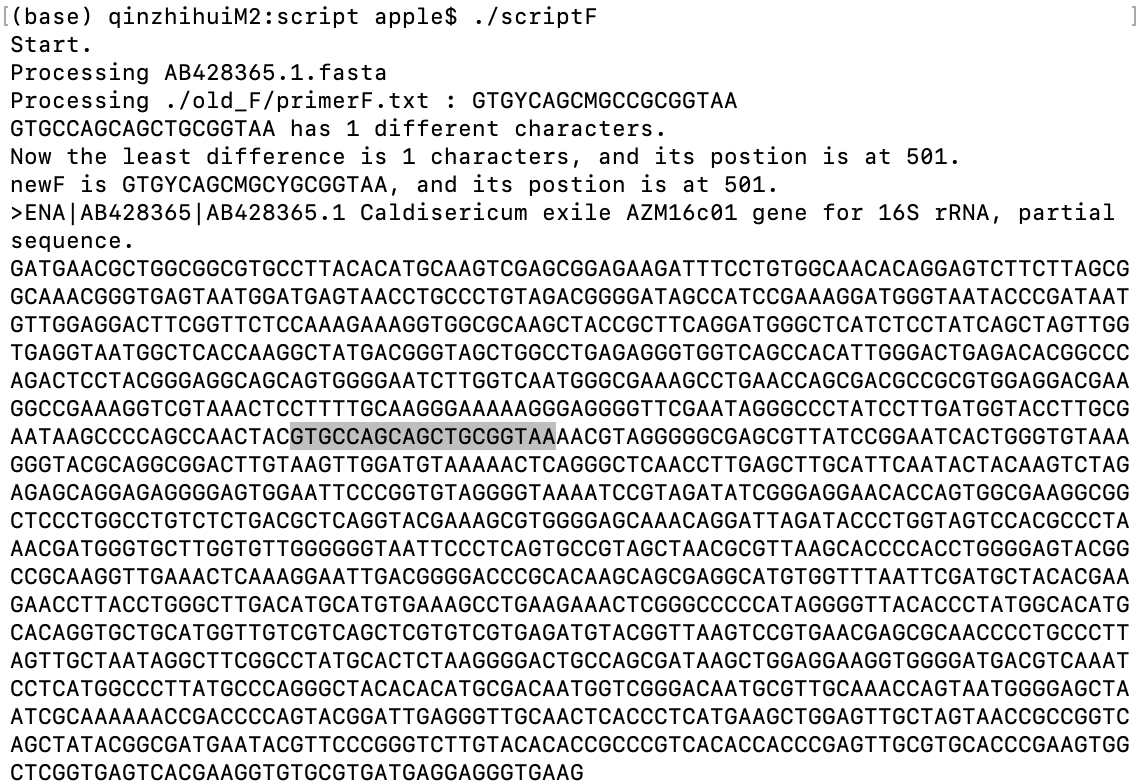

Supplement: Supplementary file 1 [file Data_Sheet_1.zip › supporting/Gene sequences and script execution results/BA-515F-806R and its improved primers/Caldisericaceae/BA-515F-806R-M3-F.png]

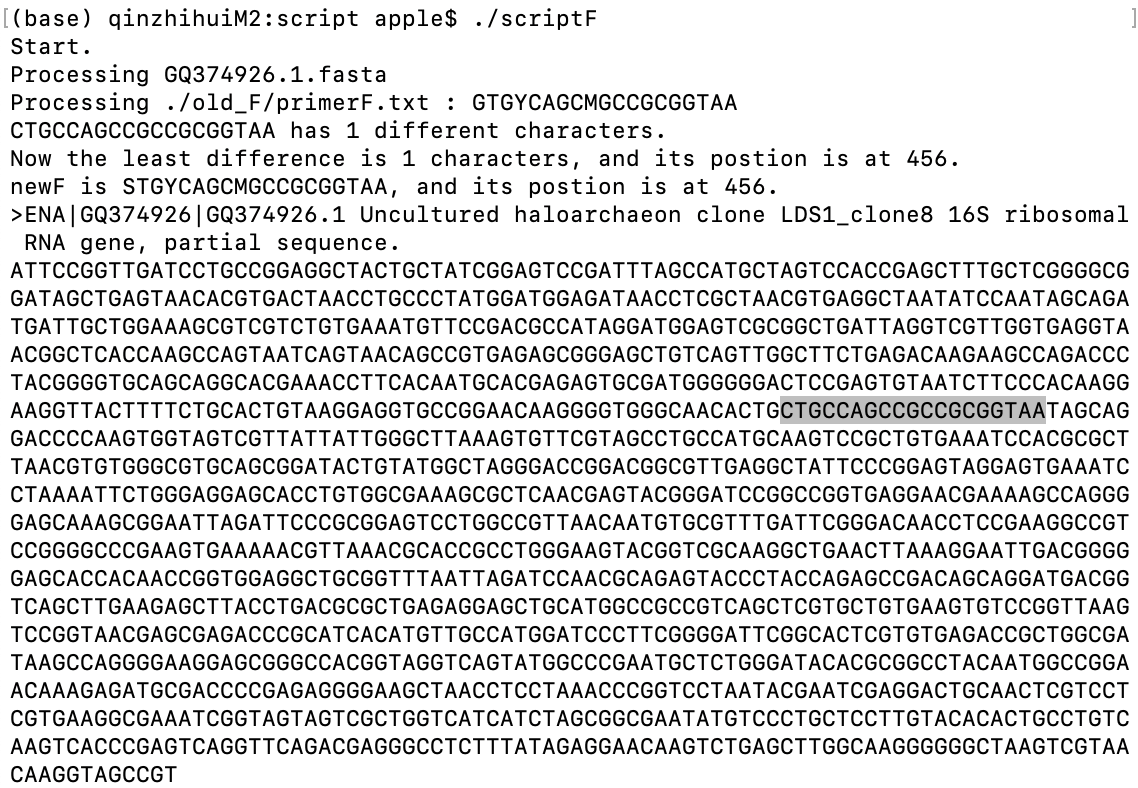

Supplement: Supplementary file 1 [file Data_Sheet_1.zip › supporting/Gene sequences and script execution results/BA-515F-806R and its improved primers/Nanohaloarchaeota/BA-515F-806R-M5-F.png]

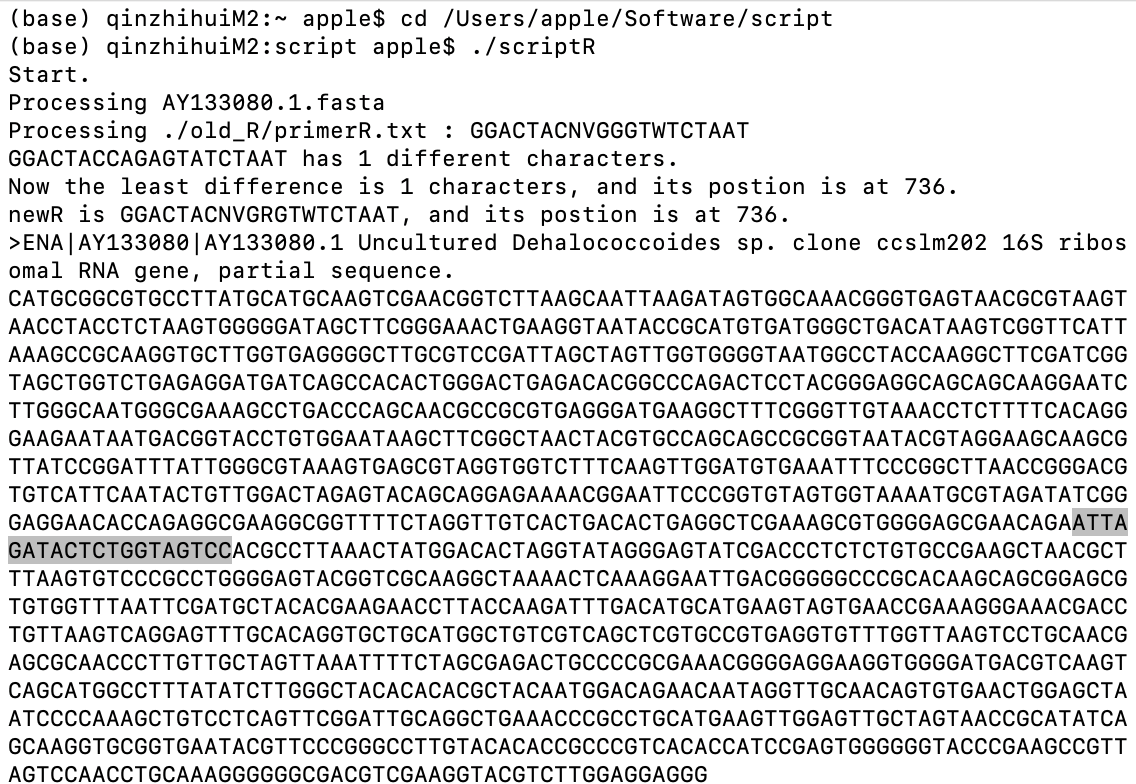

Supplement: Supplementary file 1 [file Data_Sheet_1.zip › supporting/Gene sequences and script execution results/BA-515F-806R and its improved primers/Dehalococcoides/BA-515F-806R-M1.png]

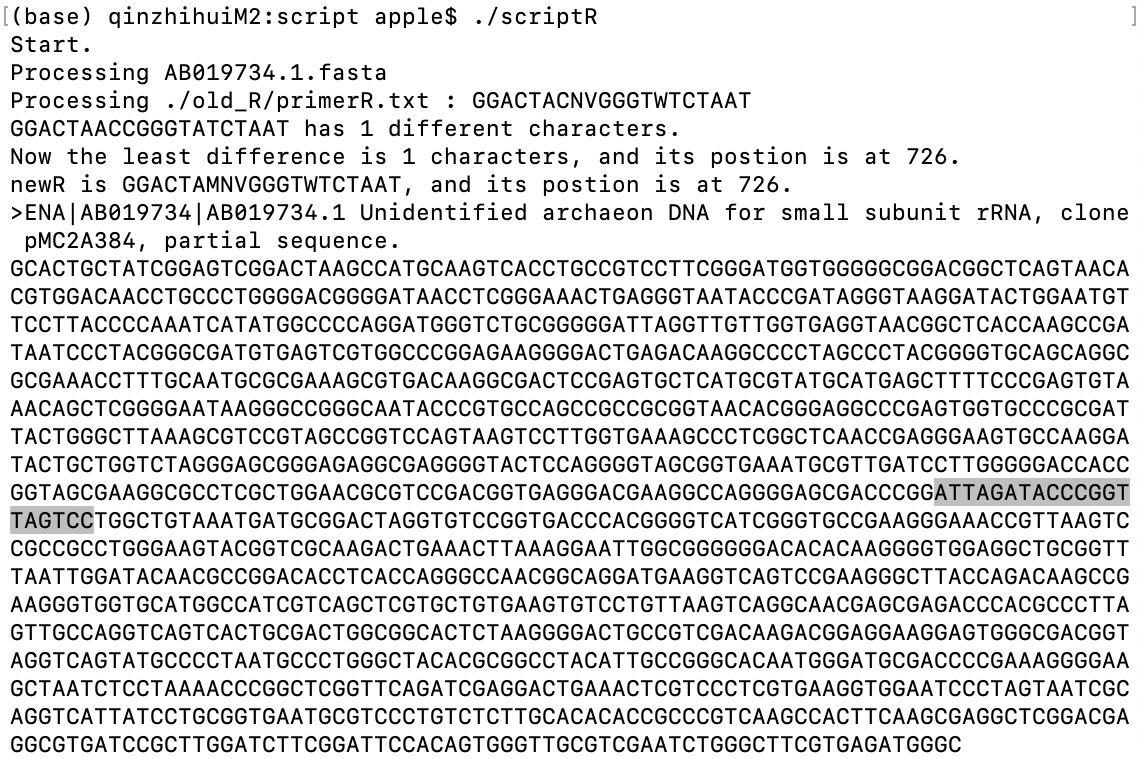

Supplement: Supplementary file 1 [file Data_Sheet_1.zip › supporting/Gene sequences and script execution results/BA-515F-806R and its improved primers/Iainarchaeota/BA-515F-806R-M4-R.png]

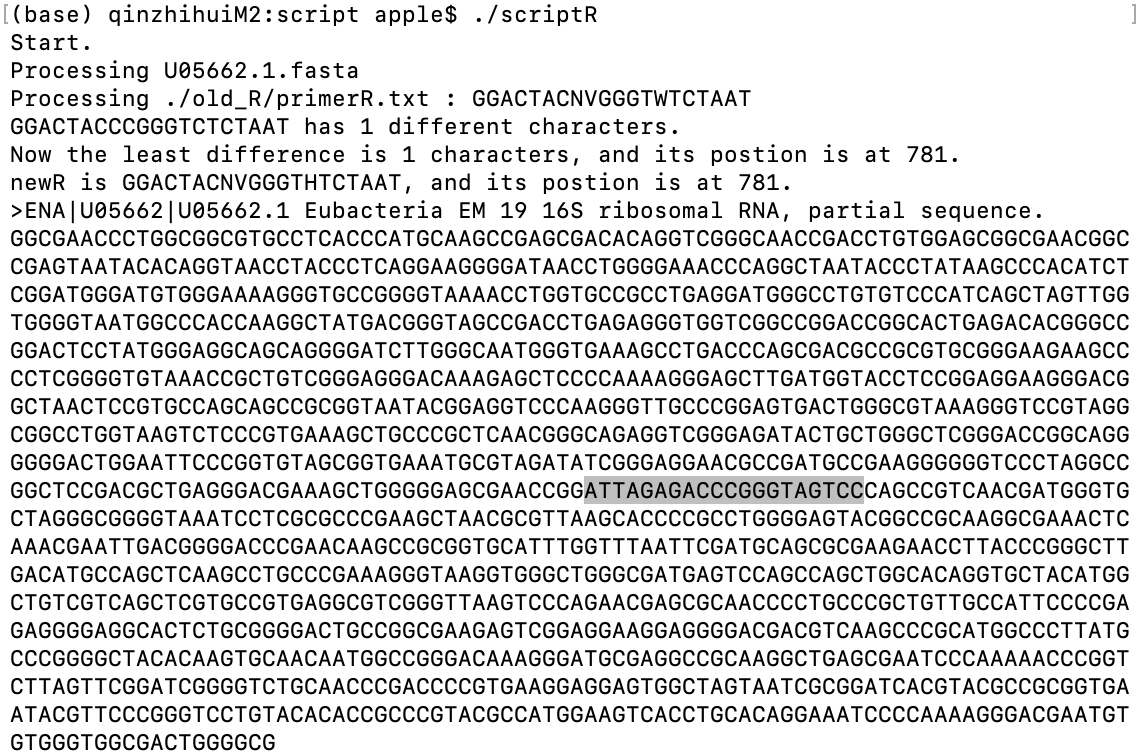

Supplement: Supplementary file 1 [file Data_Sheet_1.zip › supporting/Gene sequences and script execution results/BA-515F-806R and its improved primers/Calescamantes/BA-515F-806R-M2-R.png]

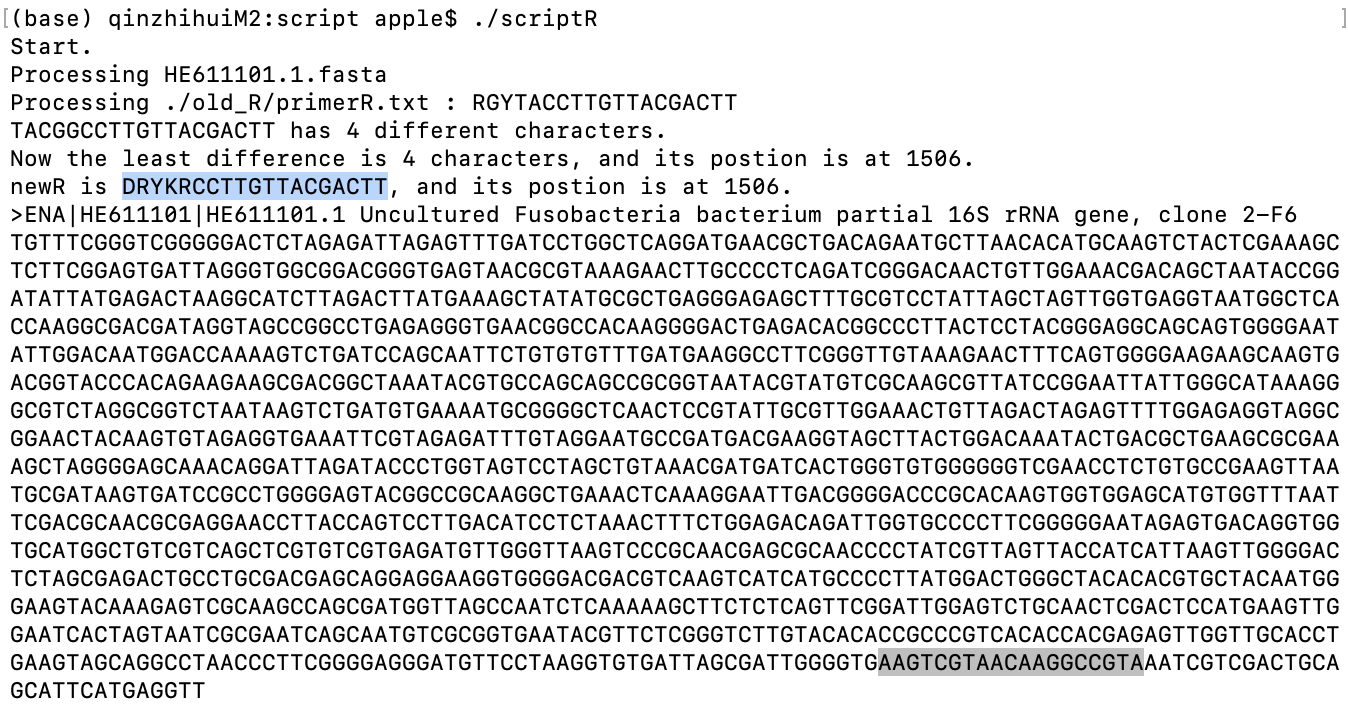

Supplement: Supplementary file 1 [file Data_Sheet_1.zip › supporting/Gene sequences and script execution results/B-27F-1492R and its improved primers/Fusobacteriota/27F-1492R-M-R1.png]
